# Supplementary material for: Longitudinal single-subject neuroimaging study reveals effects of daily environmental, physiological, and lifestyle factors on functional brain connectivity
Source: PLoS Biol. 2024 Oct 8;22(10):e3002797. doi: 10.1371/journal.pbio.3002797 (PMC11460715; doi:10.1371/journal.pbio.3002797)
Supplement: S1 Text — This includes description of the pilot studies and information about additional analysis and quality controls for the full dataset. (DOCX) [file pbio.3002797.s001.docx]

**Longitudinal single-subject neuroimaging study reveals effects of daily environmental, physiological and lifestyle factors on functional brain connectivity**

In this supplementary information document, we provide a description of the pilot studies conducted to assess the feasibility of the project and obtain data for statistical power computations. We also provide information about additional analysis and quality controls for the full dataset.

**Pilot Study Data**

We ran three different pilot studies to assess the feasibility of the study, how well the data collection systems worked, and the ability of the subject to endure the protocol. The first pilot study consisted of behavioral data only. Its aim was to test the PVT task and the feasibility of the data collection using smartphones and wearables. In the second pilot study, the subject performed the n-back task to test possible learning effects. The third pilot study gathered MRI and behavioral data. In this pilot study, we wanted to test the MRI sequences, assess the data quality of fMRI data, and test the subject’s reactions to the protocol. Here we will present the data and results from these three pilot studies. Data and code are available according to the Data availability and Code availability sections of the manuscript.

**Pilot study I: behavioral data**

The first pilot study started on July 6th, 2020 and lasted 15 days. The subject (AMT) collected data from external factors using the smartring and the smartphone app. She also performed the PVT task (see Methods) every night after 8pm but before she went to bed. The task is depicted in S-1 Fig A. The test was performed on a Windows laptop at home, in the same room every night. The subject was instructed to continue her normal life routine. Pilot I data and code are openly available in the Zenodo release^3^ <https://doi.org/10.5281/zenodo.13208496>.

*Data analysis*

All data were processed according to the preprocessing pipeline established in this document (see Analysis Plan, Behavioral preprocessing). To visually assess learning processes, we plotted the scores from the PVT as a function of time. To confirm whether learning processes occurred, we employed the Augmented Dickey Fuller (ADF) test to check for non-stationarity (i.e. presence of a trend in the time series). If results from the ADF test suggest a PVT score has a trend, we also fitted a simple linear regression model to assess the sign of the resulting slope (β). This sign will inform us whether the subject is answering faster or slower as the task is rehearsed over days. To evaluate the feasibility of the data collection using smartphones and wearables, we analyze how many data points are missing during the pilot study I period.

*PVT learning effects*

To determine if there was a learning process, we plotted the PVT scores[^1^](https://paperpile.com/c/gQLl7E/XSlHL) against time (see S-2 Fig) and observed no noticeable trend. Results from the ADF test confirm that there is no trend in the mean 1/RT (p<0.05), number of lapses (p<0.01), lapse probability (p<0.01), and performance time series (p<0.01). However, the results also suggested non-stationarity for median RT (p=0.99), slow 1/RT (p=0.17), and fastest 10% RT (p=0.18). Consequently, we also applied a linear regression model for these scores. We found no important trend for the median RT (β=0.8, R^2^=0.18), a small trend for slow 1/RT (β = -0.02, R^2^=0.43), and a trend for fastest 10% RT (β=1.5, R^2^=0.42). Nevertheless, these results imply slower reactions as the task is rehearsed daily. Given there are no noticeable trends in most of the PVT scores and the found trends do not imply better performance, we conclude there are no significant learning effects for the PVT task.

*Sensor data*

Our sensor data can be classified into three categories: (i) daily-sampled, (ii) frequently-sampled, and (iii) event-triggered. Daily sampled data include~~s~~ variables measured by the smartring and smartwatch, where a web-service processes the data and we retrieve the daily aggregated values. Consequently, we should have one datapoint per day. Frequently-sampled data refers to those smartphone sensors where data are regularly sampled within a time window (tw<10 min). Thus, we know we should have at least one data point every 10 minutes. For this type of sensor, we can employ binning strategies to aggregate the data. Finally, event-triggered sampled data refers to those smartphone sensors where data are only collected once an event happens, e.g. when a call is received.

Because of the nature of the event-related sensors, we will not use them to assess missing data. In contrast, for the daily-sampled and frequently-sampled sensors, we plotted one variable from each sensor (e.g. steps from the smartring) highlighting those times where data are missing in red (see S-3 Fig). In addition, we computed the ratio of missing data points against the total number of data points we know we should have. Results in the S-3 Fig show that most of the sensors have good data quality with less than 10% missingness, providing a good proof-of-concept for the feasibility of the data collection proposed for this work.

**Pilot study II: n-back data**

The second pilot study started on September 2nd, 2020 and lasted 14 days. The subject (AMT) performed the n-back cognitive tasks^2^ (see Methods) every night after 8pm but before she went to sleep. The task is depicted in S-1 Fig B. The test was performed on a Windows laptop at home, in the same room every night without any intervention or changes in her normal routine. Pilot II data and code is openly available in the Zenodo release^3^ <https://doi.org/10.5281/zenodo.13208496>.

*Data analysis*

Data from the n-back task were processed according to the preprocessing pipeline established in this document (see Analysis Plan, Behavioral preprocessing). To visually examine if learning processes occurred, we plotted the number of correct, wrong, and missing answers from the n-back tasks as a function of time. For each score, we also employed an ADF test to assess the presence of a trend in the time series.

*N-back learning processes*

According to the ADF test, the number of missing answers is non-stationary for 1-back (p=0.49) and 2-back (p=0.34) tasks. Similarly, the number of correct answers is found non-stationary for 1-back (p=0.67) and 2-back (p=0.60) tasks. These results are seen in S-4 Fig, where the number of correct and missing answers have high variation at the beginning, and after a few days, the variation slowly stabilizes. As expected, this variability is higher and takes longer to stabilize in the 2-back task, as the memory load is higher compared to the 1-back task. Conversely, we found the number of incorrect answers to be stationary for both, 1-back (p<0.001) and 2-back (p<0.01). These results are also shown when analyzing the task difficulty threshold d’, which is a robust indicator widely used in psychophysics.

Based on the plotted data (S-4 Fig), the learning process was not linear. Moreover, our subject has developed a comfortable strategy to solve the tasks after seven days for the 1-back, and ten days for the 2-back. Consequently, to avoid capturing any learning effects in the MRI data, the subject must rehearse the tasks for a minimum of ten days prior to the first scan. The task difficulty threshold also shows the learning process and stability of the task difficulty over time. For changes in visual gratings, we see a stability in d’ after three days for the 1-back task, and seven days for the 2-back task. Similarly, we observe that d’ stabilizes after five days for 1-back tasks.

**Pilot study III: MRI and behavioral data**

In the final pilot study, we collected MRI and behavioral data simulating part of the protocol for one and a half weeks. This part of the protocol included MRI sessions (see Methods, Brain imaging data acquisition), smartphone and smartring data collection. Unlike the full protocol, no cognitive tasks were performed in between the scanner sessions and no smartwatch was used. The pilot aimed to test the MRI sequences, assess MRI and sensor data quality, and test the subject’s reactions to the protocol, in particular her endurance of the scanning time. The pilot study was also useful to select (i) the MRI preprocessing strategy, (ii) the sleep and activity features we will use in the analyses, (iii) and the imputation method for missing data.

The subject attended three MRI scanning sessions in August 2021 (on the 12th, 16th, and 18th). She also collected behavioral data from August 2, 2021 to September 7, 2021. Similar to pilot study I and pilot study II, the subject was instructed to continue her normal life routine.

MRI data from this pilot study were also employed to compute the sample size needed for the PVT and n-back tasks using neuropower[^4^](https://paperpile.com/c/gQLl7E/hjebr). In addition to the three aforementioned MRI sessions, we included a fourth one (December 18th, 2021) to produce better sample size estimates.

Behavioral data from Pilot III and code is openly available in the Zenodo release^3^ <https://doi.org/10.5281/zenodo.13208496>. MRI data is available on request (see Data availability section).

*Data analysis*

MRI data were preprocessed according to the pipeline established in the Analysis Plan (see Analysis Plan, MRI preprocessing). To evaluate our denoising strategy, we plotted R^2^ maps, carpet plots, and voxel time series comparing the fmriprep^14,15^ output files and the denoised files. We also plotted R^2^ maps between the global signal and the denoised files to motivate our choice of not regressing the global signal. Further, to assess the MRI data quality, we calculated the temporal signal-to-noise ratio (tSNR) for each task, in each session, and plotted the corresponding maps. Finally, to appraise the fMRI data quality, we plotted the framewise displacement (FD) yielded by fmriprep^14,15^ for each task in each session; peaks over 0.5 (FD>0.5) are labeled as poor quality data.

Behavioral data collected with sensors were processed according to the pipeline established in this document (see Analysis Plan, Behavioral preprocessing). Similar to pilot study I, to evaluate the sensor data quality, we plotted one feature per sensor and calculated the ratio of missing data points; we labeled a sensor as ‘low quality’ when more than 40% of the data were missing at random.

Pilot study III data also allowed us to select the measured variables we will use from the external sensors. These features were selected based on their correlations with other features (correlation heatmaps), their use in previously published research, and their ease of interpretation (e.g. sleep staging has not yet been validated for wearables and therefore will not be used).

Further, pilot study III data allowed us to run two additional analyses. With this data, we could analyze the imputation method we will employ for missing behavioral data. In this case, we created artificial missingness in our time series and assigned new values using eight different imputation methods. Then, for each imputation method, we calculated the mean squared error (MSE) of the imputed values vs. the real values and selected the method that yielded the lowest median MSE with the narrowest distribution. Secondly, we ran inter-subject correlation (ISC) analysis using the ISCToolbox[^5^](https://paperpile.com/c/gQLl7E/qGbbQ) on the pilot study data and compared it to the results from part 2 of the Budapest dataset[^6^](https://paperpile.com/c/gQLl7E/VQPjD).

Finally, we used MRI data from pilot study III and an additional MRI session (December 18th, 2021) to compute power analysis for the PVT and n-back tasks using neuropower[^4^](https://paperpile.com/c/gQLl7E/hjebr). We computed second-level GLM for both tasks based on the four sessions. For the PVT task, we employed the RT for each trial in our design matrix and computed the activation maps for those trials where the subject pressed the button in less than 500 ms. For the n-back task, we computed the difference between the 2-back and 1-back conditions based on the reaction time answers to each trial on each block. With this, we aim to ensure the number of samples we need to detect a significant event with our two tasks.

*MRI data preprocessing*

The preprocessing stage is divided into minimal preprocessing and denoising. Minimal preprocessing is performed by fmriprep^14,15^, while denoising includes detrending, regression of confounds, and filtering. We will focus on the impacts of the denoising choices since that is the most flexible part of the preprocessing. To achieve this, we generated a series of plots that allow us to understand the impact of our denoising strategy spatially and temporally (see S-5 Fig). The aim is to have a preprocessing strategy that removes noise, but that is not so harsh that it removes the signal of interest. In that sense, we aim for a moderate effect on the voxels in the cortex. Here, we have chosen to regress the twenty-four head movement parameters, signals from the cerebrospinal fluid (CSF), white matter (WM), their derivatives and powers, heart and respiratory rate, framewise displacement (FD), and spikes as detected by fmriprep^14,15^.

From S-5 Fig A, we see that denoising affects all parts of the brain. Brain plots were generated with nilearn^8^. The correlation coefficients also show that there are few harsh effects (ρ>0.85), an observation that is confirmed by the distribution of the correlation values (S-5 Fig B). Moreover, in terms of individual voxels, we see that our denoising strategy removes some noise from individual voxel time series (S-5 Fig D) and the global signal (S-5 Fig C), where denoising has cleaned the dropout signal at the beginning of the time series. S-5 Fig E and F provide information about the global signal and its relationship with individual voxels. In S-5 Fig E, we can see the areas of the brain where the global signal is highly correlated with voxels time series. Most of these correlations are in the occipital and parietal regions, which are areas of interest. On the other hand, S-5 Fig F compares the global signal with the time series of a voxel chosen at random. In this case, the correlation is low. Since the global signal appears to be highly correlated with parietal areas, we decided not to regress it in the primary analysis. However, because the global signal is a contentious topic in the field, we will also run the analysis with a denoising strategy that includes global signal regression. Finally, we compared the carpet plots of the BOLD signal before and after denoising (S-5 Fig G). Compared to the carpet plot before denoising (top), the carpet plot after denoising (bottom) was less noisy, with fewer evident stripes, and better resembled white noise, a characteristic that is desirable[^7^](https://paperpile.com/c/gQLl7E/xVq9w).

Based on the previous analysis, these results suggest that our denoising strategy suits our aims, as it effectively removes most of the noise (S-5 Fig G), in areas of interest (S-5 Fig A) while preserving signal characteristics (S-5 Fig C and D).

*MRI data quality*

Here, fMRI quality was measured by two factors, tSNR (S-6 Fig) and framewise displacement (S-7 Fig). In general, a good fMRI signal has a strong tSNR that is similar across runs (e.g. tasks and sessions). At the same time, tSNR is expected to vary across brain areas due to artifacts, coils, and the subject’s arousal levels[^9^](https://paperpile.com/c/gQLl7E/hqIXJ). S-6 Fig shows very similar tSNR values across tasks and sessions with higher tSNR in the occipital regions and lower tSNR in areas closer to air-tissue borders. Lower tSNR was also observed close to the prefrontal areas due to the number of coils used in the scanner (<https://www.aalto.fi/sites/g/files/flghsv161/files/2020-03/ami_equipment_modified_30_ch_coil.pdf>). Despite the variations, the dataset tSNR is still high (mean+-std) and comparable to other datasets[^6^](https://paperpile.com/c/gQLl7E/VQPjD).

Similar to the tSNR, the dataset showed good data quality in terms of framewise displacement (S-7 Fig). In all sessions and for all the tasks, the subject could lay still without moving considerably (no FD peaks bigger than 0.5). Moreover, during 99.2% of the time, the FD was lower than 0.2 (i.e. data with little movement), suggesting good tolerance of the subject to the time in the scanner. In this particular head coil, two channels have been removed from the front to avoid restrictions to the visual field. Consequently, some drop-out to the signal at the prefrontal area is expected.

*Sensor data quality*

Similar to our analysis of the sensor data for pilot study I, we plotted one variable from each sensor and highlighted those times where data were missing in red (see S-8 Fig). The ratio of missing data points was also computed. Results in the S-8 Fig show that most of the sensors have good data quality with less than 10% missingness. Unlike pilot study I, two sensors from pilot study III showed more than 15% missing data. This is expected as the pilot study III duration was longer. On the other hand, the sensors that had most missing data points do not require the subject’s input, suggesting that the missingness is due to a technical problem, rather than subject’s tolerance to the protocol. This is further confirmed by the fact that the battery sensor had less than 2% missingness.

*Selection of features from the smartring*

The smartring web service provides fifty-five features based on the device measurements. Although informative, there are too many to be considered in the main model. Some of the features are scores developed by the manufacturer whose calculations are unknown to us. Some features are highly correlated with each other, which is an undesirable characteristic in the data. Finally, some features have not been frequently used in the literature and may be difficult to interpret. Therefore, we analyzed the pilot study III smartring data to select the features that we will include in the main analysis. To achieve this, we plotted correlation matrices between all smartring features (S-9 Fig A).

Based on the aforementioned reasons, we discarded those features that (i) are scores computed by the manufacturer, (ii) are related to sleep stages, and (iii) are highly correlated with other scores. S-9 Fig B and C show the selected measurements after discarding features based on (i) and (ii) for sleep and activity data, respectively. Even after discarding, we noticed some highly correlated values remain. For sleep data (S-9 Fig B), total bedtime, sleep timing, and awake time were highly correlated with other features. Consequently, we will not include them in the main analysis. For activity data (S-9 Fig C), activity burn, total burn, daily movement, low activity time, medium activity time, rest time, and average MET were highly correlated with at least two other features. Therefore, we will discard those activity features. Due to interpretability, we will only choose steps and inactive time.

*Selection of imputation method*

As previously demonstrated, missingness in sensor data is likely to happen despite the quality controls (see Pilot study data, sensor quality control). Therefore, we need a procedure to handle missingness and avoid discarding observations with missing data. To select a proper method, we created artificial missingness on the collected data by deleting random values. Those deleted values accounted for 20% of the data, which is higher than previously observed. After deleting the values, we employed eight different imputers and compared their yielded value with the true value we deleted. We repeated this procedure 10000 times and plotted their distributions (see S-10 Fig). For sleep data (S-10 Fig A), the lowest median effects were yielded with mean, linear, and mice[^10^](https://paperpile.com/c/gQLl7E/AzQJi) imputers. However, the linear and mice imputers had more outliers than the mean imputer. Consequently, we will employ the mean imputer to fill in missing values for sleep data. For ESM data (S-10 Fig B), we observed a similar performance of most of the imputers in terms of median and standard deviation. Since there is no noticeable difference and to be consistent, we will also employ the mean imputer to fill in missing values for ESM data.

*ISC analysis*

The subject watched part 2 from “The Grand Budapest Hotel” in the three sessions from Pilot study III. We used these runs to compute the intersubject correlation between different viewings. We later compared these results with the ISC computed over the same part from the openly available Budapest dataset[^6^](https://paperpile.com/c/gQLl7E/VQPjD). Our aim was to check that we obtain similar activations in our data compared to the Budapest dataset (see S-11 Fig). In general, we saw activations in similar areas, but with stronger correlation values. This may be due to the low number of sessions, but the fact that we used data from the same subject may also play a role, as between-subject variability is generally higher than within-subject variability in fMRI data[^11^](https://paperpile.com/c/gQLl7E/DLlUm). Moreover, when examining ISC between different sessions, we observed little difference between them. For example, ISC between the first and second sessions was similar to the ISC between the first and third sessions (see S-12 Fig). Although limited by the number of datapoints, we observed that the repetition of the movie does not significantly decrease ISCs, suggesting that the engagement of the subject remained equal across the three movie sessions.

**Data quality: Main dataset**

Following the protocol, we collected 133 days of data from a single subject from three distinct sources of data: MRI and MRI-related, smartphones and wearable devices, and cognitive tests. For each data source, we computed the usability ratio by dividing the number of functional files by the expected total file count. For highly-sampled data (e.g., from smartphones), we aggregated the data into 1-minute bins before computing the usability ratio.

S-13 Fig displays the overall usability results, showing that per day, we collected at least 60% of the expected data points. MRI data collection was consistent during the thirty sessions: we only rescheduled one session due to technical problems and two sessions due to Easter holidays. fMRI data shows low movement, with average FD between 0.07 and 0.17 (see S-14 Fig A and S-15 Fig). Higher movement tended to be observed in the latter runs of the session, especially during the n-back paradigm.

BIOPAC data was successfully collected for all sessions and tasks, except for the movie-watching run in the last session. Conversely, the eye-tracking data proved to be more challenging to collect. S-13 Fig shows that eye-tracker data was corrupted for at least one task in five sessions. The data corruption was notably more frequent late in the session (see S-14 Fig B). While the eye-tracker was well-calibrated and gathered data effectively in the early runs, its performance declined in the final runs (i.e. movie and n-back). This issue appears to be linked to our protocol, which included short breaks for the subject to adjust her legs and arms, with instructions to keep her head still. These movements may have inadvertently shifted her pupil alignment relative to the eye-tracker camera, negatively impacting its ability to collect quality data in the later runs.

Despite the reported difficulties on collecting passive smartphone data[^12^](https://paperpile.com/c/gQLl7E/GKUR3), we estimate that 60% of the possible daily data points were collected in our study. Note that this estimate is based on the recorded battery level which is a frequently-sampled sensor, and other sensors that are event-based (e.g. screen status) may have a lower quality and need to be assessed independently. For example, GPS tracking exhibited 18% data losses over the 133 days. Similar missingness rates were obtained for active smartphone data (i.e. questionnaires), with the subject missing answering morning questions at a higher rate than the evening questions. Based on the subjects’ data, this may be related to her circadian rhythm.

Heart rate variability and respiration rate exhibited the highest percentage of missing data, with available data only ranging from 20% to 40%. This reduced performance is linked to the difficulties in computing heart rate variability and respiration rate from PPG. These measurements typically require conditions with minimal light and movement and, as a result, the reliability of these signals is primarily during sleep periods.

**Regressors**

Initially, we chose a set of variables from external sensors for the regression models, based on pilot data (see Supplementary Information, Selection of features from the smartring). However, subsequent analysis revealed significant multicollinearity among these variables, undermining the reliability of statistical inferences in our models. To address this, we revised our selection criteria, focusing on variables with a Variance Inflation Factor (VIF) below 5 and correlation coefficients below 0.7. Our process was structured as this: first, we computed regression models as outlined in Hypotheses 1, 2, and 3 using all potential variables. Then we systematically eliminated the variable with the highest VIF. We repeated these steps until all remaining variables had a VIF under 5. Finally, we further excluded any remaining variables that demonstrated high mutual correlation (detailed in S-16 Fig). It is important to note that since this problem specifically affects hypotheses H1, H2, and H3, we used only thirty data points for computing the correlations. These thirty data points refer to the behavioral, physiological, and lifestyle variables from the day before the scanner session as outlined in the hypotheses. After completing this procedure, we identified seventeen variables, which are detailed in S-3 Table.

**H1: supplementary results**

Here, we list the main supplementary material for H1. Complete tables with all the models and uncorrected values are available in the GIT^16^, in the *results/H1* folder. Unprocessed study data can be found in the Zenodo dataset release[^13^](https://paperpile.com/c/gQLl7E/G4ba). S-4 Table shows statistically significant results for the main analysis. S-5 Table and S-17 Fig show results for analysis using a second parcellation. S-6 Table and S-18 Fig show results when we regressed the global signal from the data. Finally, S-7 Table shows significant results for the global efficiency for different network thresholds.


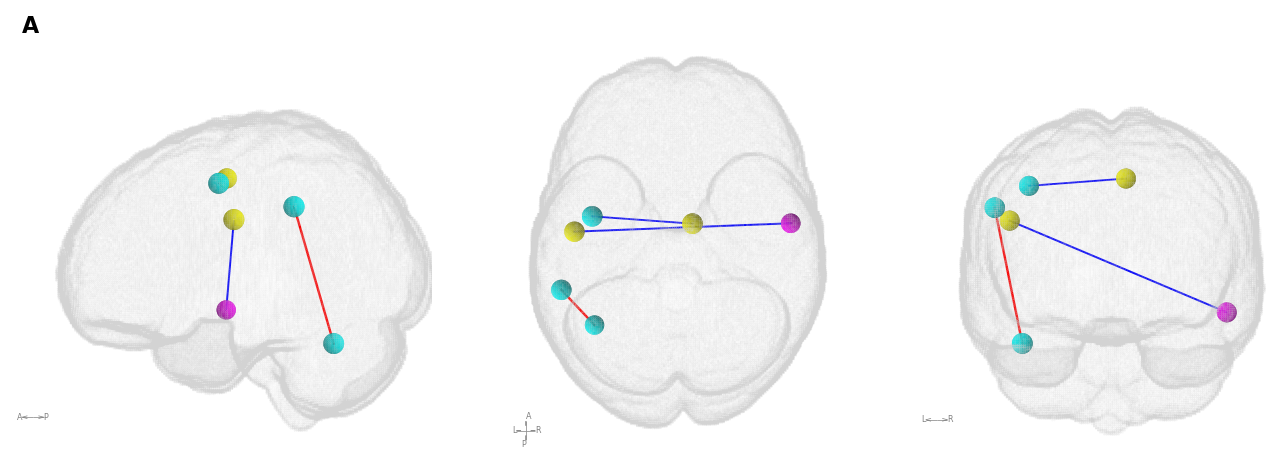


**H2: supplementary results**

Here, we list the main supplementary material for H2. Complete tables with all the models and uncorrected values are available in the GIT^16^, in the *results/H2* folder. Unprocessed study data can be found in the Zenodo dataset release[^13^](https://paperpile.com/c/gQLl7E/G4ba). S-8 Table shows numerical results for the main analysis. S-9 Table and S-19 Fig show results for analysis using a second parcellation. S-10 Table and S-20 Fig show results when we regressed the global signal from the data. Finally, S-11 Table shows significant numerical results for participation coefficient for different parcellations and thresholds.

**H3: supplementary results**

Here, we list the main supplementary material for H3. Complete tables with all the models and uncorrected values are available in the GIT^16^, in the *results/H3* folder. Unprocessed study data can be found in the Zenodo dataset release[^13^](https://paperpile.com/c/gQLl7E/G4ba). S-12 Table shows numerical results for the main analysis. S-13 Table and S-21 Fig show results for analysis using a second parcellation. S-14 Table and S-22 Fig show results when we regressed the global signal from the data. Finally, S-15 Table shows significant numerical results for participation coefficient for different parcellations, denoise strategies, and thresholds.

**H4: supplementary results**

Here, we list the main supplementary material for H4. Uncorrected p-values are in the GIT^16^ *results/H4* folder. Unprocessed study data can be found in the Zenodo dataset release[^13^](https://paperpile.com/c/gQLl7E/G4ba). S-16 Table shows significant results from Mantel tests using different models, parcellations, and denoise strategies. S-23 Fig, S-24 Fig, and S-25 Fig show the movie quality data and the impact of applying different scrubbing percentages to the quality and number of available fMRI data points.

**H5: supplementary results**

Here, we list the main supplementary material for H5. Complete tables with all the models and uncorrected values are available in the GIT^16^, in the *results/H5* folder. Unprocessed study data can be found in the Zenodo dataset release[^13^](https://paperpile.com/c/gQLl7E/G4ba). S-17 Table and S-18 Table shows statistical significant results for the main analysis. S-26 Fig and S-27 Fig show results for analysis using other network thresholds. S-19 Table and S-28 Fig show the statistical significant results when analyzing the data with a second parcellation. Finally, S-20 Table and S-29 Fig show results when we regressed the global signal from the data.

**H6: supplementary results**

Here, we list the main supplementary material for H6. Complete tables with all the models and uncorrected values are available in the GIT^16^, in the *results/H6* folder. Unprocessed study data can be found in the Zenodo dataset release[^13^](https://paperpile.com/c/gQLl7E/G4ba). S-21 Table and S-22 Table shows statistical significant results for the main analysis. S-30 Fig and S-31 Fig show results for analysis using other network thresholds. S-23 Table and S-32 Fig show the statistical significant results when analyzing the data with a second parcellation. Finally, S-24 Table and S-33 Fig show results when we regressed the global signal from the data.

**H7: supplementary analysis**

Here, we list the main supplementary material for H7. Complete tables with all the models and uncorrected values are available in the GIT^16^, in the *results/H7* folder. Unprocessed study data can be found in the Zenodo dataset release[^13^](https://paperpile.com/c/gQLl7E/G4ba). S-25 Table and S-26 Table shows statistical significant results for the main analysis. S-34 Fig and S-35 Fig show results for analysis using other network thresholds. S-27 Table and S-36 Fig show the statistically significant results when analyzing the data with a second parcellation. Finally, S-28 Table and S-37 Fig show results when we regressed the global signal from the data.

**H8: supplementary analysis**

Here, we list the main supplementary material for H8. Complete tables with all the models and uncorrected values are available in the GIT^16^, in the *results/H8* folder. Unprocessed study data can be found in the Zenodo dataset release[^13^](https://paperpile.com/c/gQLl7E/G4ba). S-29 Table and S-38 Fig show the results when the data is analyzed using a sliding window with 2TRs. S-30 Table and S-39 Fig show the results when the data is analyzed using a sliding window with 4TRs. S-31 Table and S-40 Fig show results when we regressed the global signal from the data. Finally, S-41 Fig show the comparison between the LOO and 20% leave-one-out methods in the classification accuracy for three different regions of interest.

**Additional analysis: supplementary results**

Complete tables with all the models and uncorrected values are available in the GIT^16^, in the *results/supplementary* folder. Unprocessed study data can be found in the Zenodo dataset release[^13^](https://paperpile.com/c/gQLl7E/G4ba). S-42 Fig displays the brain region showing a significant linear association between sleep duration and activations in the PVT task-fMRI.

# **References**

1. [Basner, M., and Dinges, D.F. (2011). Maximizing sensitivity of the psychomotor vigilance test (PVT) to sleep loss. Sleep *34*, 581–591.](http://paperpile.com/b/gQLl7E/XSlHL)

2. [Salmela, V., Salo, E., Salmi, J., and Alho, K. (2018). Spatiotemporal Dynamics of Attention Networks Revealed by Representational Similarity Analysis of EEG and fMRI. Cereb. Cortex *28*, 549–560.](http://paperpile.com/b/gQLl7E/XijCS)

3. Triana, A., & Glerean, E. (2024). AnaTomomi/protocol: stage1_v3 (stage1_v3). Zenodo. <https://doi.org/10.5281/zenodo.13208496>

4. Durnez, J., Degryse, J., Moerkerke, B., Seurinck, R., Sochat, V., Poldrack, R. A., & Nichols, T. E. (2024). “Neuropower” Software. <https://github.com/neuropower/neuropower-core>

5. [Kauppi, J.-P., Pajula, J., and Tohka, J. (2014). A versatile software package for inter-subject correlation based analyses of fMRI. Front. Neuroinform. *8*, 2.](http://paperpile.com/b/gQLl7E/qGbbQ)

6. Visconti di Oleggio Castello, M., Chauhan, V., Jiahui, G., & Gobbini, M. I. (2020). An fMRI dataset in response to “The Grand Budapest Hotel”, a socially-rich, naturalistic movie. Scientific Data, 7(1), 383.

7. [Power, J.D. (2017). A simple but useful way to assess fMRI scan qualities. Neuroimage *154*, 150–158.](http://paperpile.com/b/gQLl7E/xVq9w)

8. Nilearn contributors, Chamma, A., Frau-Pascual, A., Rothberg, A., Abadie, A., Abraham, A., Gramfort, A., Savio, A., Cionca, A., Thual, A., Kodibagkar, A., Kanaan, A., Pinho, A. L., Idrobo, A. H., Kieslinger, A.-S., Rokem, A., Mensch, A., Vijayan, A., Duran, A., … Nájera, Ó. (2024). nilearn (0.10.4). Zenodo. https://doi.org/10.5281/zenodo.10948303.

9. [Murphy, K., Bodurka, J., and Bandettini, P.A. (2007). How long to scan? The relationship between fMRI temporal signal to noise ratio and necessary scan duration. NeuroImage *34*, 565–574.](http://paperpile.com/b/gQLl7E/hqIXJ) [10.1016/j.neuroimage.2006.09.032](http://dx.doi.org/10.1016/j.neuroimage.2006.09.032)[.](http://paperpile.com/b/gQLl7E/hqIXJ)

10. [van Buuren, S., and Groothuis-Oudshoorn, K. (2011). **mice**: Multivariate Imputation by Chained Equations in*R*. Journal of Statistical Software *45*.](http://paperpile.com/b/gQLl7E/AzQJi) [10.18637/jss.v045.i03](http://dx.doi.org/10.18637/jss.v045.i03)[.](http://paperpile.com/b/gQLl7E/AzQJi)

11. [Thirion, B., Pinel, P., Mériaux, S., Roche, A., Dehaene, S., and Poline, J.-B. (2007). Analysis of a large fMRI cohort: Statistical and methodological issues for group analyses. NeuroImage *35*, 105–120.](http://paperpile.com/b/gQLl7E/DLlUm) [10.1016/j.neuroimage.2006.11.054](http://dx.doi.org/10.1016/j.neuroimage.2006.11.054)[.](http://paperpile.com/b/gQLl7E/DLlUm)

12. [Onnela, J.-P. (2021). Opportunities and challenges in the collection and analysis of digital phenotyping data. Neuropsychopharmacology *46*, 45–54.](http://paperpile.com/b/gQLl7E/GKUR3)

13. Triana, A. & Glerean, E. (2024). Effects of daily environmental, physiological, and lifestyle factors on functional brain connectivity [Data set]. Zenodo. <https://doi.org/10.5281/zenodo.10571956>

14. [Esteban, O., Markiewicz, C.J., Blair, R.W., Moodie, C.A., Isik, A.I., Erramuzpe, A., Kent, J.D., Goncalves, M., DuPre, E., Snyder, M., et al. (2019). fMRIPrep: a robust preprocessing pipeline for functional MRI. Nat. Methods *16*, 111–116.](http://paperpile.com/b/gQLl7E/5ydN)

15. Esteban, O., Ross B., Christopher J. M., Shoshana L. B., Craig M., Feilong M., Ayse I., et al. (2018). “FMRIPrep.” Software. Zenodo. https://doi.org/10.5281/zenodo.852659.

16. Triana, A., & Glerean, E. (2024). AnaTomomi/externalfactors_on_functionalconnectivity: EF_BC_v2 (EF_BC_v2). Zenodo. <https://doi.org/10.5281/zenodo.13208812>
