# Supplementary figures and images for: Longitudinal single-subject neuroimaging study reveals effects of daily environmental, physiological, and lifestyle factors on functional brain connectivity
Source: PLoS Biol. 2024 Oct 8;22(10):e3002797. doi: 10.1371/journal.pbio.3002797 (PMC11460715; doi:10.1371/journal.pbio.3002797)

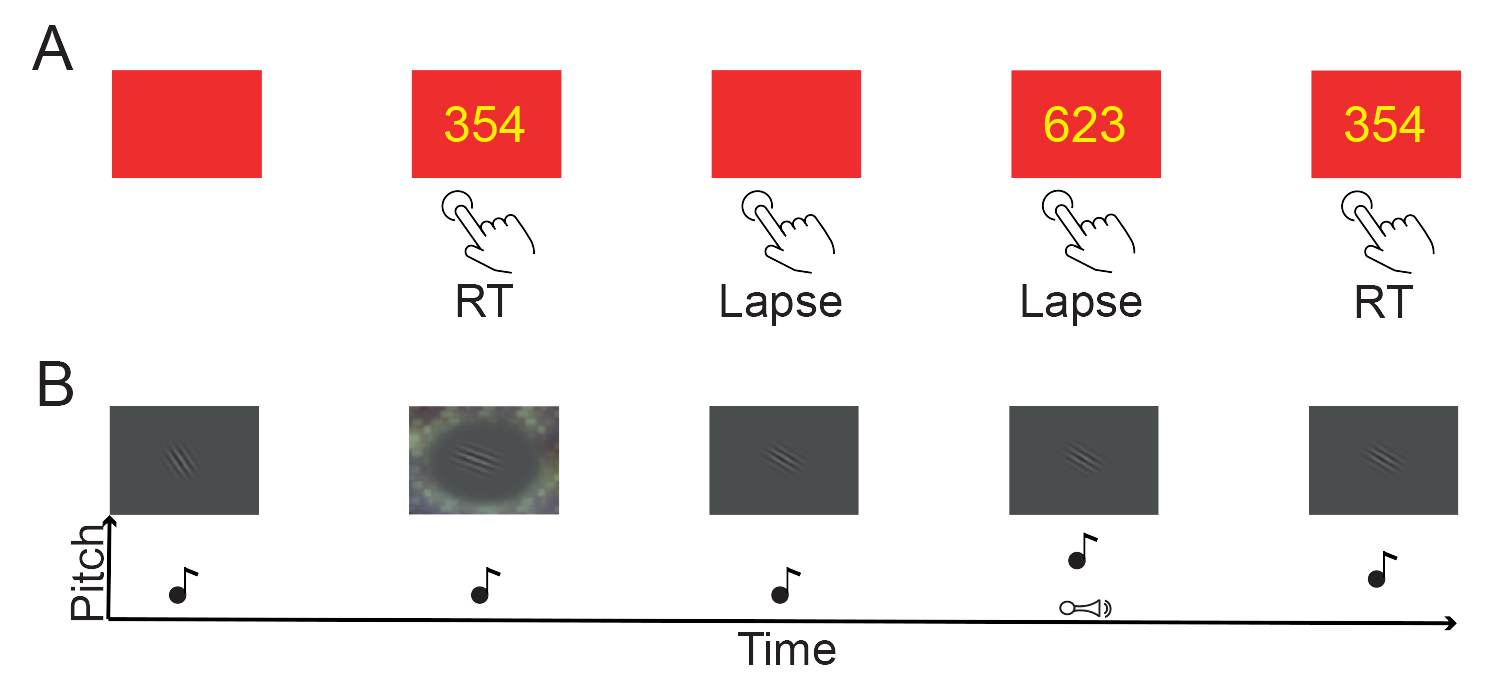

Supplement: S1 Fig — (A) PVT task [42]. The participant must observe a red screen and press a button as soon as a yellow counter appears on it. The counter shows the milliseconds elapsed between the start of the counter and the button press, i.e., reaction time (RT). Lapses are counted if the button is pressed when no stimulus is shown, or if the RT is longer than 500 ms. The period between the last response and the new stimulus varies between 2 and 10 s. (B) N-back task [43]. Auditory and visual stimuli are presented simultaneously. Only 1 stimulus (either visual or auditory) changes per trial. Auditory stimuli and visual stimuli are sinewave gratings with occasional auditory or visual distractors. The participant should press the buttons up/down when the pitch has increased/decreased. The participant should also press the buttons left/right when the grading has changed counterclockwise/clockwise. (TIF) [file pbio.3002797.s002.tif]

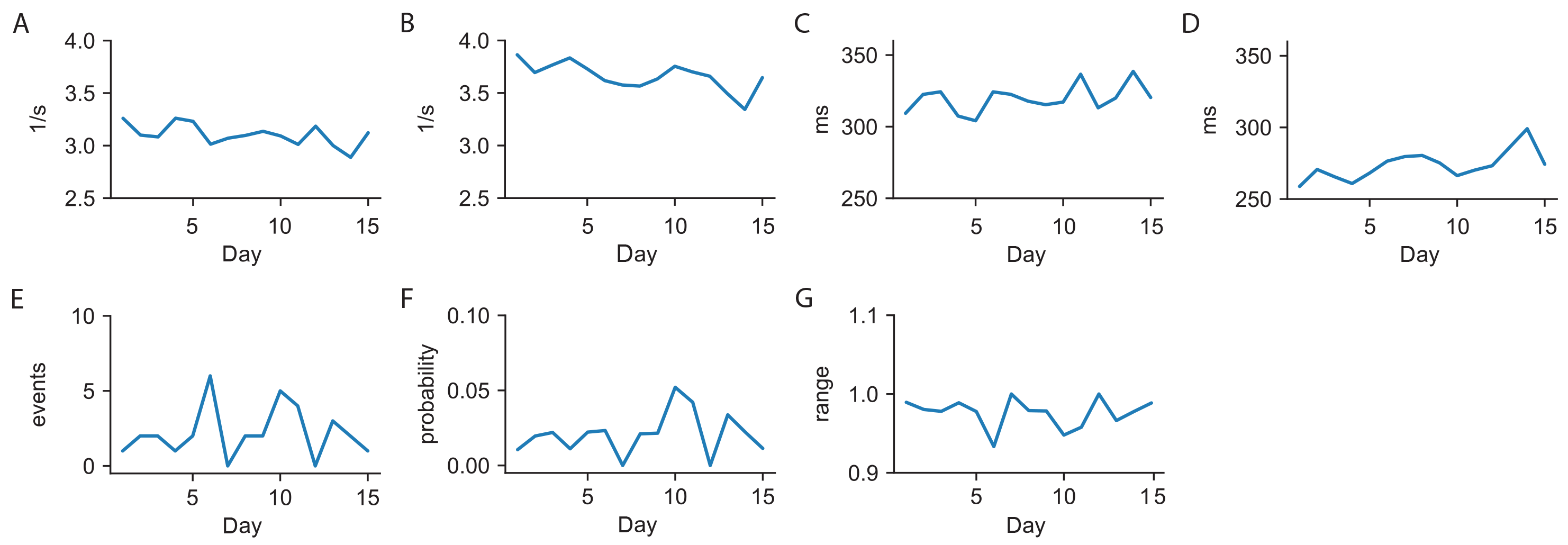

Supplement: S2 Fig — The participant tested the PVT task for 15 consecutive days (n = 15) and her performance was monitored. The PVT scores were computed according to Basner and Dinges [42]. The figure shows the scores for (A) mean 1/RT, (B) slowest 1/RT, (C) median RT, (D) fastest 10% RT, (E) number of lapses, (F) lapse probability, and (G) performance. Similar to previous reports [42], we observed no apparent learning effects in any of the scores for the PVT task. Pilot I data can be found in the Zenodo release [174]. (TIF) [file pbio.3002797.s003.tif]

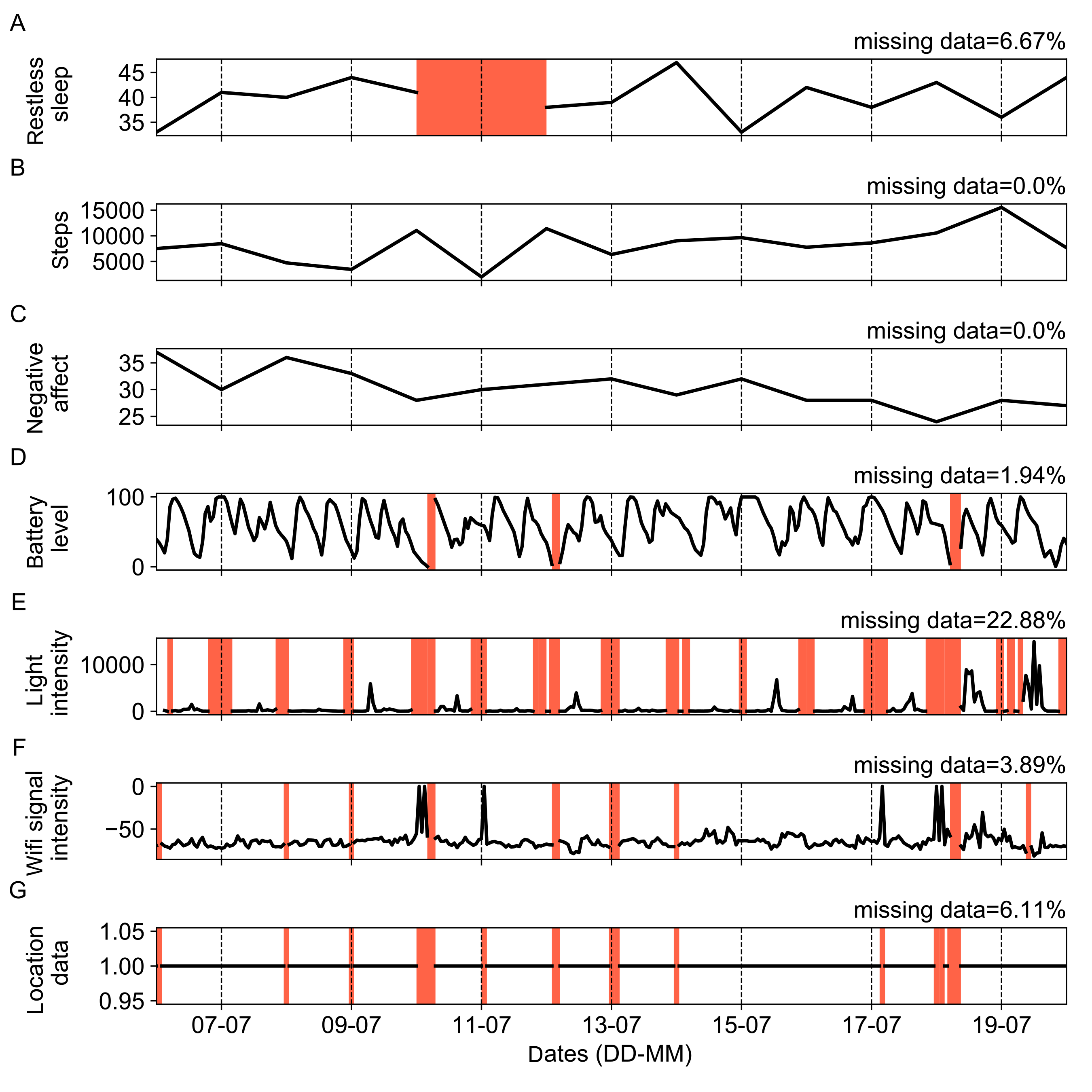

Supplement: S3 Fig — We plot 1 feature from each sensor and highlight in red the days where data are missing. We estimate the missing data ratio as the proportion of missing data points over the total number of data points each sensor should have gathered. We show the data quality for (A) sleep, (B) activity, (C) Experience Sampling Method (ESM), (D) battery, (E) light, (F) WiFi, and (G) GPS location data. Overall, the data quality is good, with most sensors losing less than 10% of the data. Only the light sensor lost approximately 20% of the data and there seems to be a pattern of lost data every second day that cannot be associated with other sensors. Pilot I data can be found in the Zenodo release [174]. (TIF) [file pbio.3002797.s004.tif]

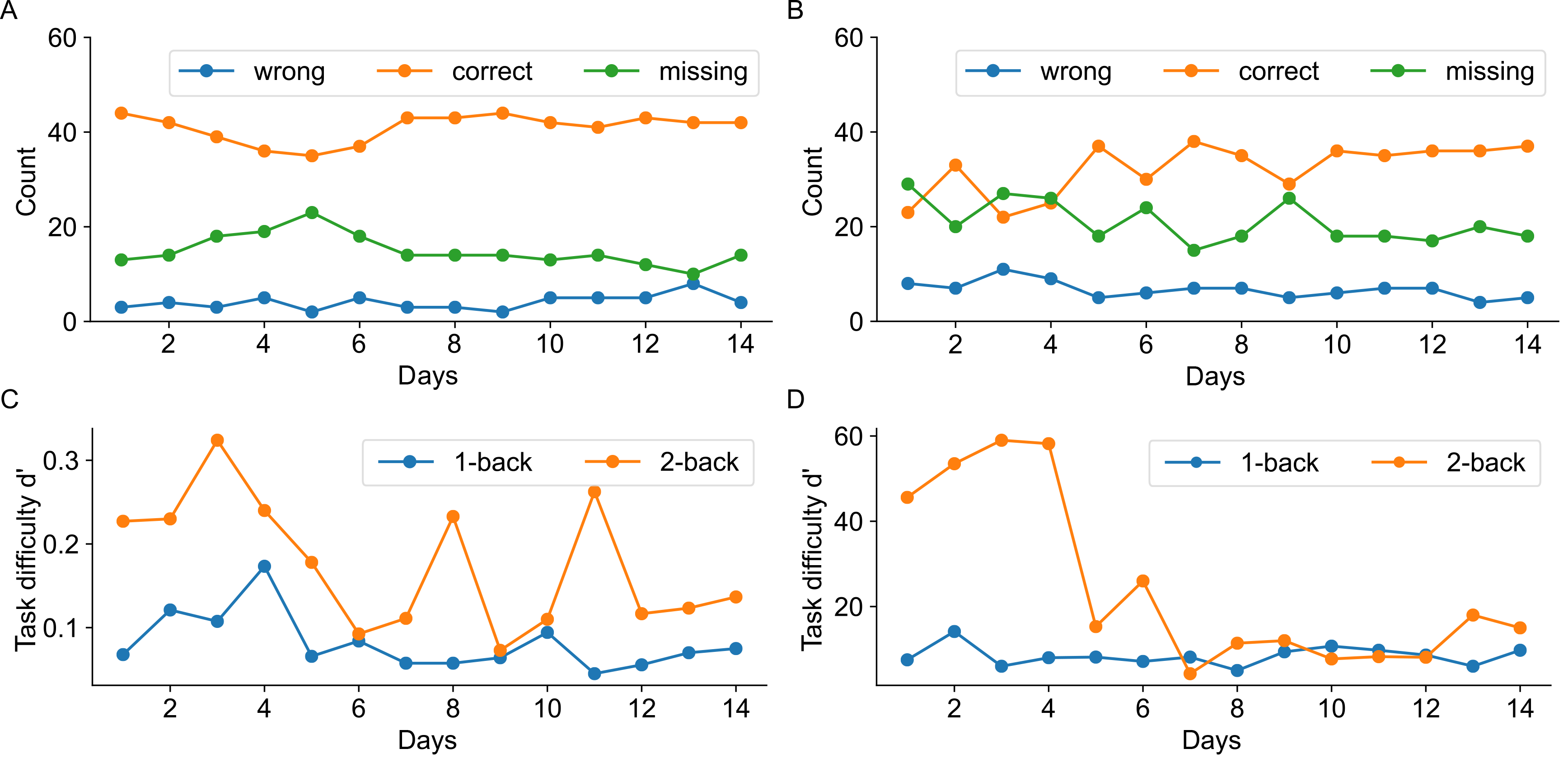

Supplement: S4 Fig — The participant tested the n-back task for 14 consecutive days (n = 14) and her performance was monitored. Three n-back scores were computed: the number of correct answers, the number of wrong answers, and the number of missing answers (i.e., the subject failed to press a button). The figure shows the scores for (A) 1-back, and (B) 2-back. For both tasks, there is high variability in the number of correct and missing answers at the beginning of the pilot study. As time goes by, the variability decreases and the number of correct and missing answers slightly oscillates over a stable count. It takes approximately 7 days for the 1-back task, and 10 days for the 2-back task to reach this stability. In addition, the task difficulty d’ was assessed for (C) pitch changes (octaves), and (D) visual angle (degrees). Pilot II data can be found in the Zenodo release [174]. (TIF) [file pbio.3002797.s005.tif]

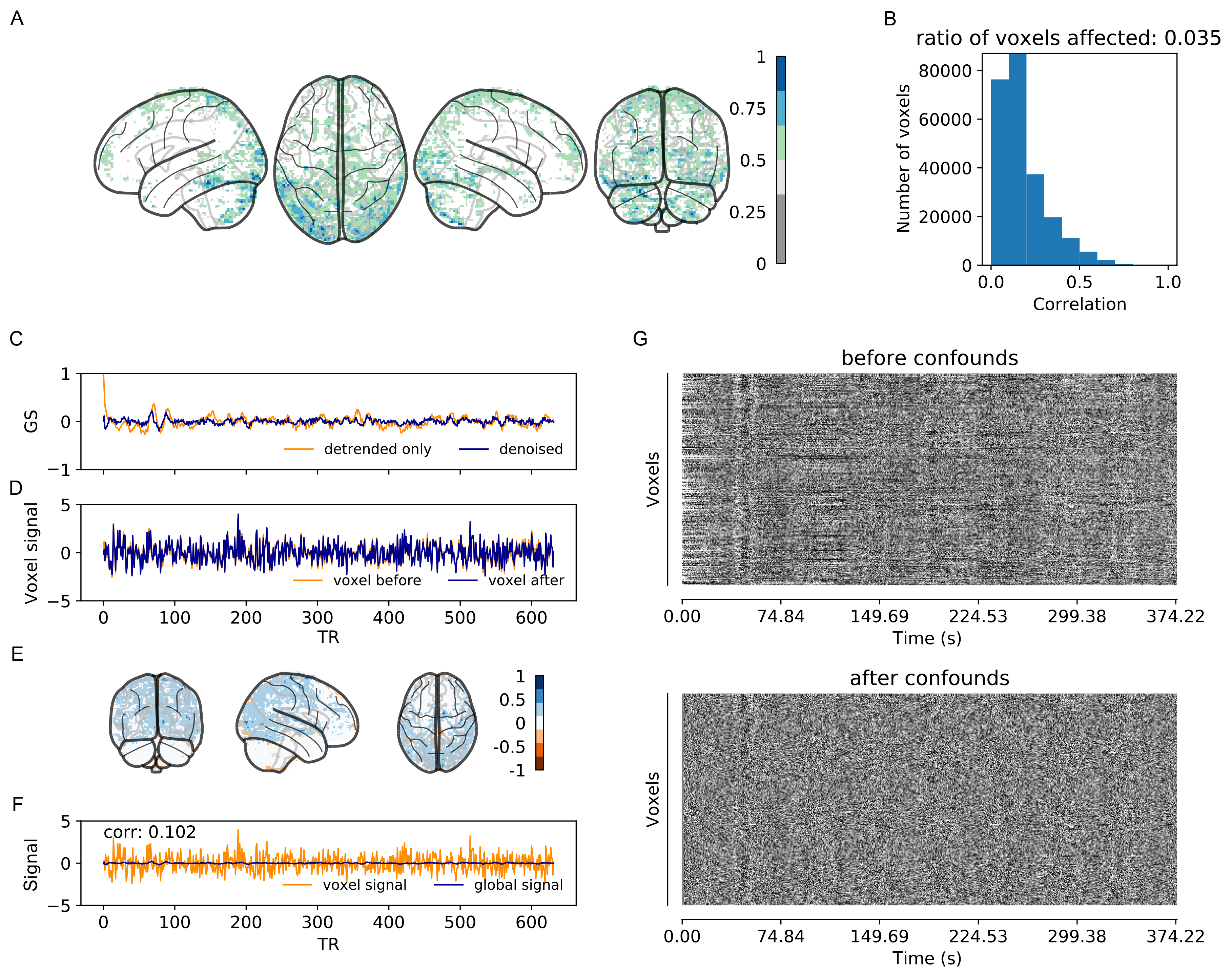

Supplement: S5 Fig — We inspected how our preprocessing choices affected the BOLD signal by comparing the images before and after denoising. Our aim is to remove the chosen confounds, without losing the signal, so a cleaning effect needs to be evident, but moderate. We see that: (A) Most of the affected voxels are in the cortex, where the signal of interest lies. Moreover, the correlation coefficient shows that the impact is moderate. (B) Similarly, the distribution of the correlation coefficient of voxels shows a low proportion of the voxels being hardly affected (i.e., harsh denoising). In this case, higher correlations imply a stronger effect of the denoising strategy. (C) The denoising strategy impacts the global signal (GS). Drift effects are apparent before regressing the confounds and filtering. (D) The denoising effect is also visible in individual voxel signals. We chose a voxel from the cortex at random and plotted its signal before and after the confound regression and filtering. Both signals looked similar, but some peaks have been attenuated after denoising (voxel after). (E) Correlation between the global signal and the brain voxels. Most of the global signal was highly correlated with voxels in the cortex and areas of interest. (F) Correlation of the global signal and a voxel from the cortex chosen at random. (G) Carpet plots [224] of the signal before and after denoising. The carpet plot after denoising (bottom) is less noisy and more greatly resembles white noise than the carpet plot before denoising (top), a feature that is desirable. Brain plots were generated with nilearn [170]. Pilot III data can be found in the Zenodo release [174]. (TIF) [file pbio.3002797.s006.tif]

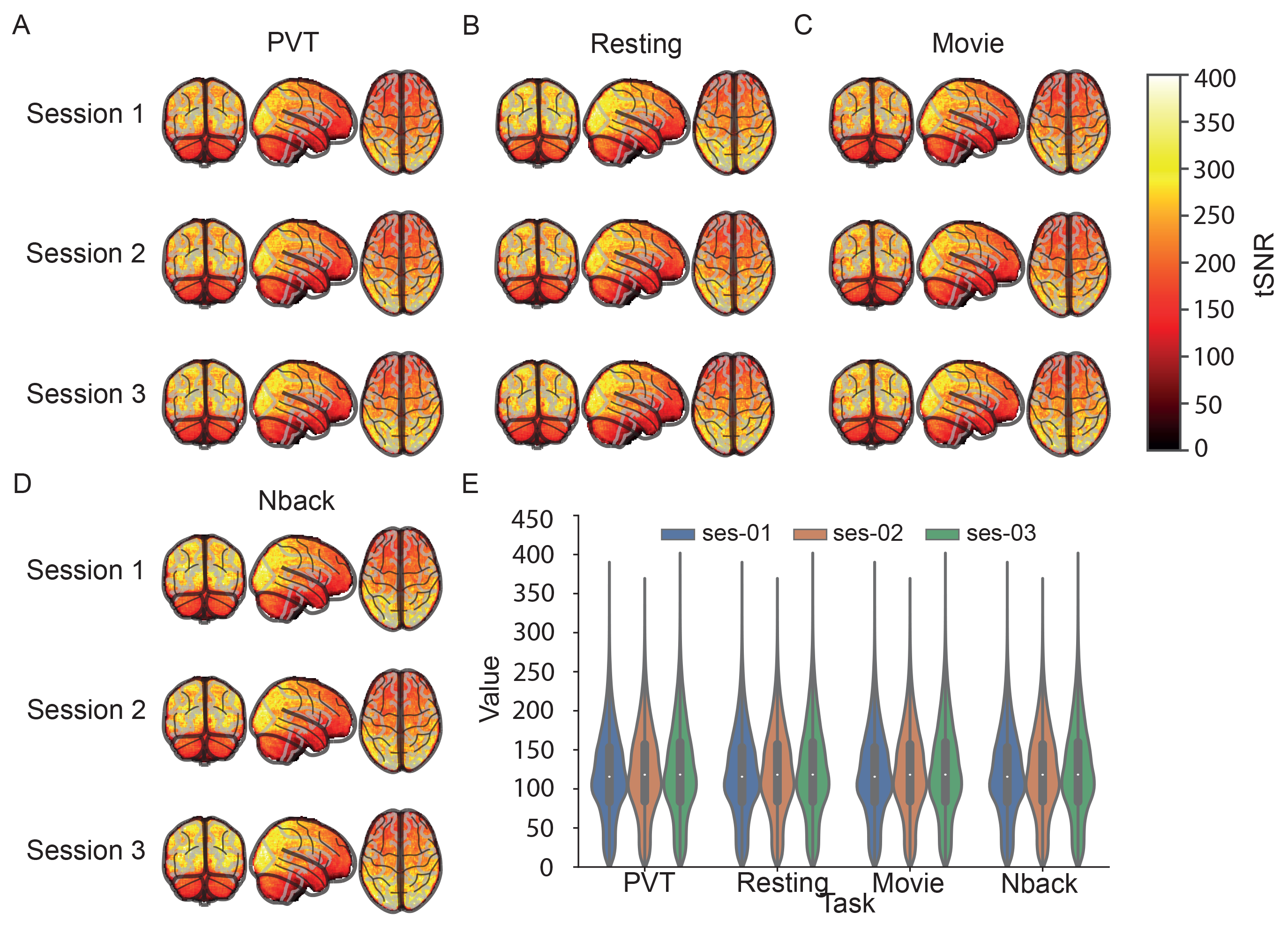

Supplement: S6 Fig — For each task and session, a tSNR map was generated by computing the ratio between mean and standard deviation for each voxel across time. This plot shows the tSNR values within a brainmask. Across tasks and sessions, the mean tSNR was 120.11. As expected, we observed signal dropout in areas close to air-tissue borders. Brain plots were generated with nilearn [170]. Pilot III data can be found in the Zenodo release [174]. (TIF) [file pbio.3002797.s007.tif]

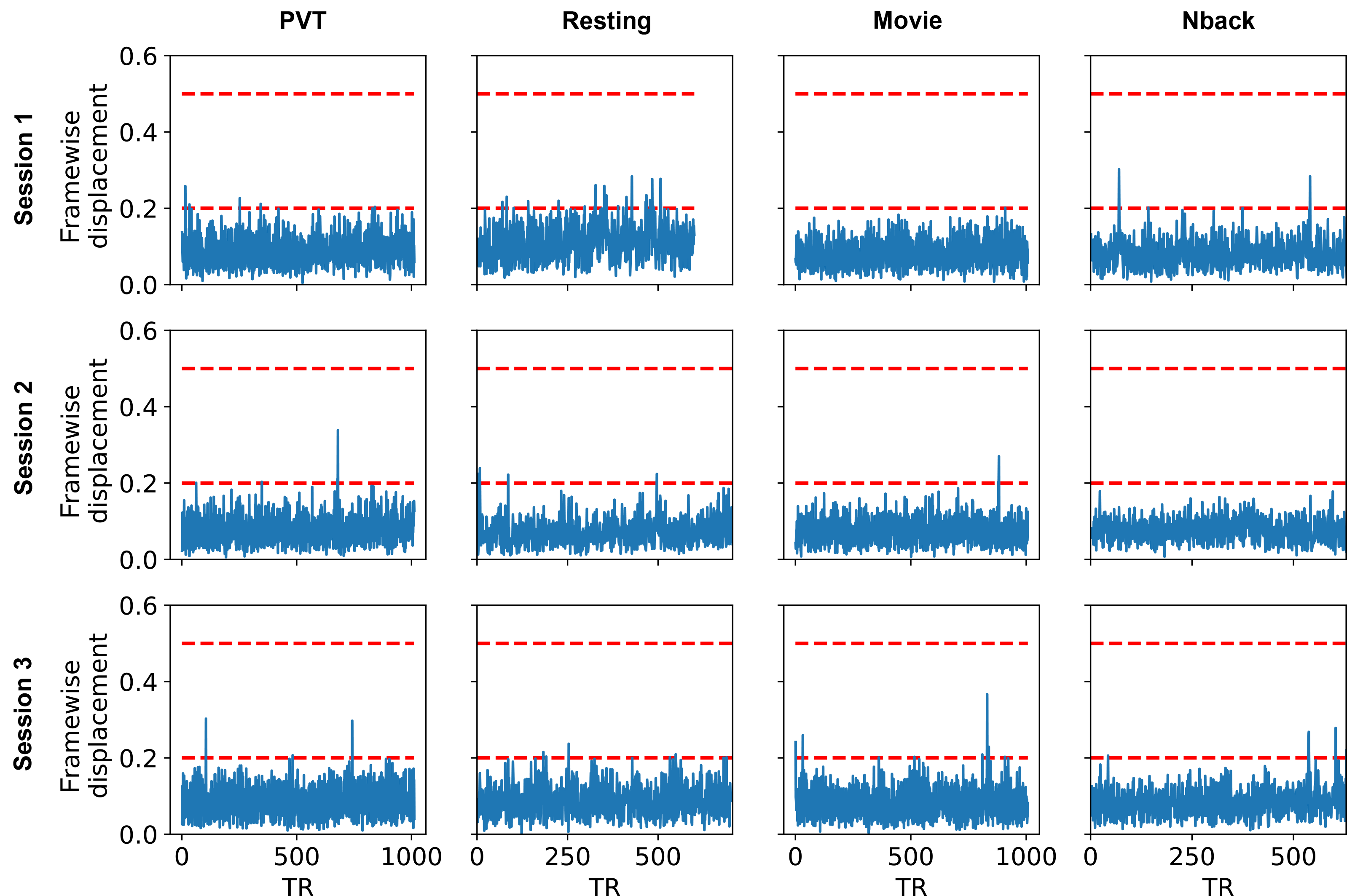

Supplement: S7 Fig — For each task and session, we plotted the FD as computed by fmriprep. In general, the signal quality was good; 99.2% of the time peaks were below 0.2 and no peaks were over 0.5. Pilot III data can be found in the Zenodo release [174]. (TIF) [file pbio.3002797.s008.tif]

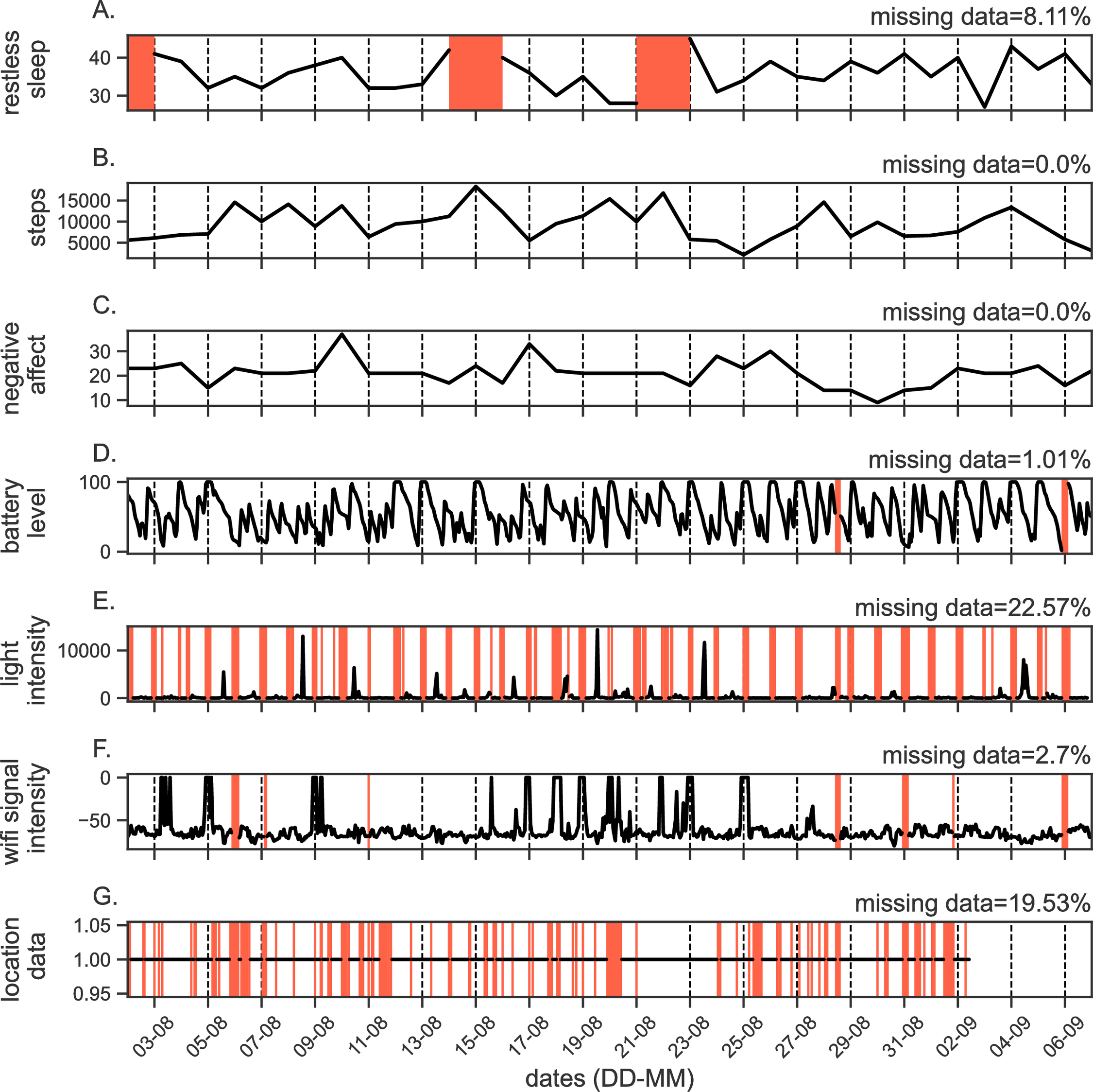

Supplement: S8 Fig — We plotted 1 feature from each sensor and highlighted in red the days where data were missing. We estimate the missing data ratio as the proportion of missing data points over the total number of data points each sensor should have gathered. We show the data quality for (A) sleep, (B) activity, (C) ESM, (D) battery, (E) light, (F) WiFi, and (G) GPS location data. Overall, the data quality was good, with most sensors losing less than 10% of the data. Light and location sensors had more than 15% missing data points. While the light sensor had a pattern, the location sensor did not. Given the battery sensor had less than 2% missing data points, the light and location sensor missingness could be associated with technical problems of their own, rather than other factors such as the subject shutting down the phone. Pilot III data can be found in the Zenodo release [174]. (TIF) [file pbio.3002797.s009.tif]

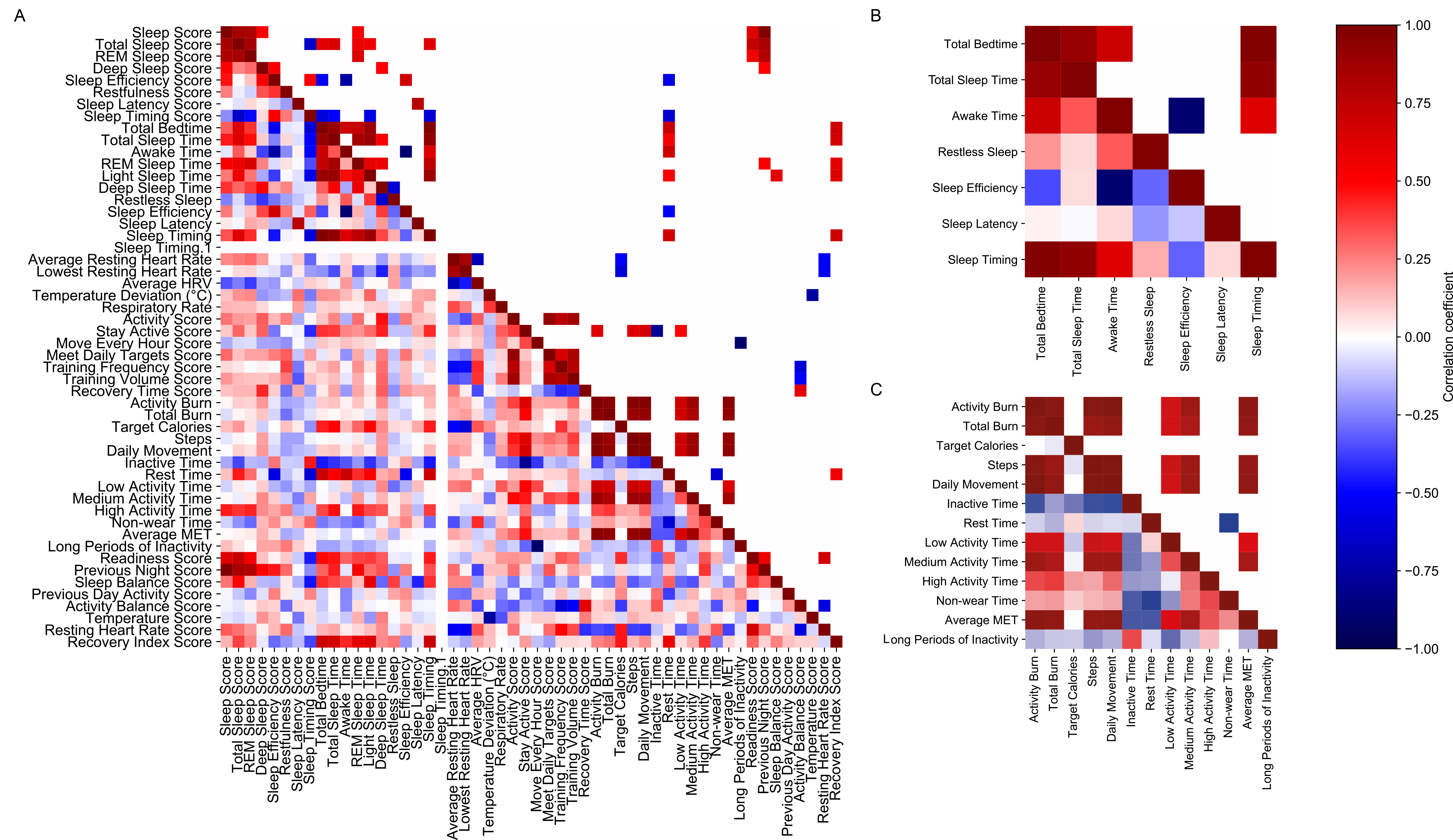

Supplement: S9 Fig — (A) Full correlation matrix. (B) Correlation between a subset of sleep features. (C) Correlation between a subset of activity features. Features in B and C were selected based on 2 reasons: they are not scores computed automatically by the manufacturer and they are not related to sleep staging. For all matrices, we thresholded the upper triangle to show correlation coefficients whose absolute value is greater than 0.5. Pilot III data can be found in the Zenodo release [174]. (TIF) [file pbio.3002797.s010.tif]

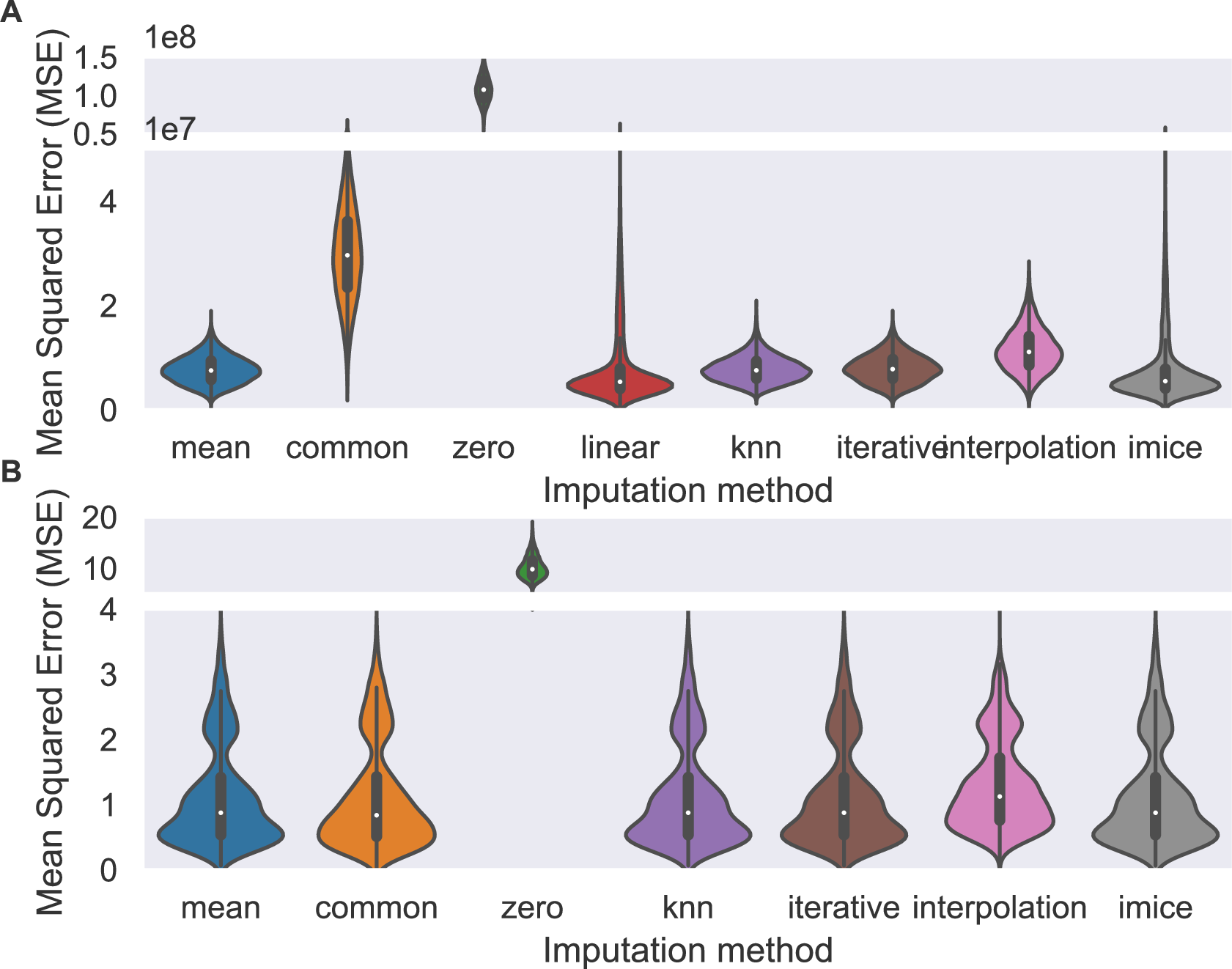

Supplement: S10 Fig — We deleted 20% of the (A) sleep and (B) ESM collected data at random. Then, we employed each imputer to generate values on the artificial missing data. Finally, we computed the mean squared error (MSE) between the true value and the value yielded by the imputer and repeated the procedure 10,000 times. The zero, mean, and common imputers replaced missing data points with zeros, the mean, and the most frequent value of the data set, respectively. The linear imputer calculates a linear model based on the existent data and uses it to predict the missing data value. The knn imputer completes missing values based on the k-nearest neighbors method. The iterative imputer estimates new values based on other features from the same dataset. Finally, mice imputes incomplete multivariate data by chained equations [225]. Pilot III data can be found in the Zenodo release [174]. (TIF) [file pbio.3002797.s011.tif]

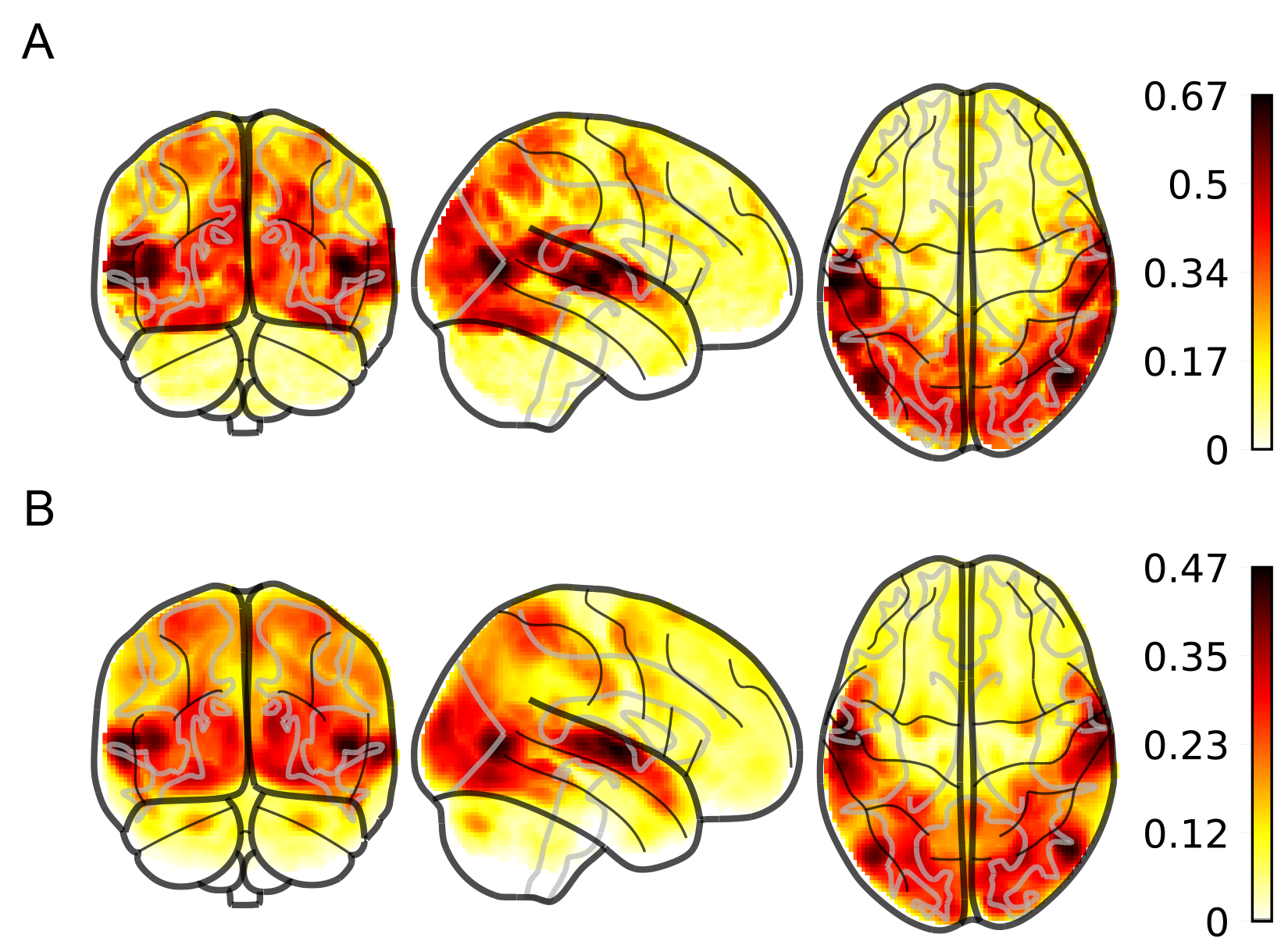

Supplement: S11 Fig — (A) ISC from the pilot study data. (B) ISC from the Budapest data set. We observe that similar areas in the brain are activated across sessions (pilot study) and subjects (Budapest). Nevertheless, the correlation values are stronger in the pilot study data. Brain plots were generated with nilearn [170]. Pilot III data can be found in the Zenodo release [174]. (TIF) [file pbio.3002797.s012.tif]

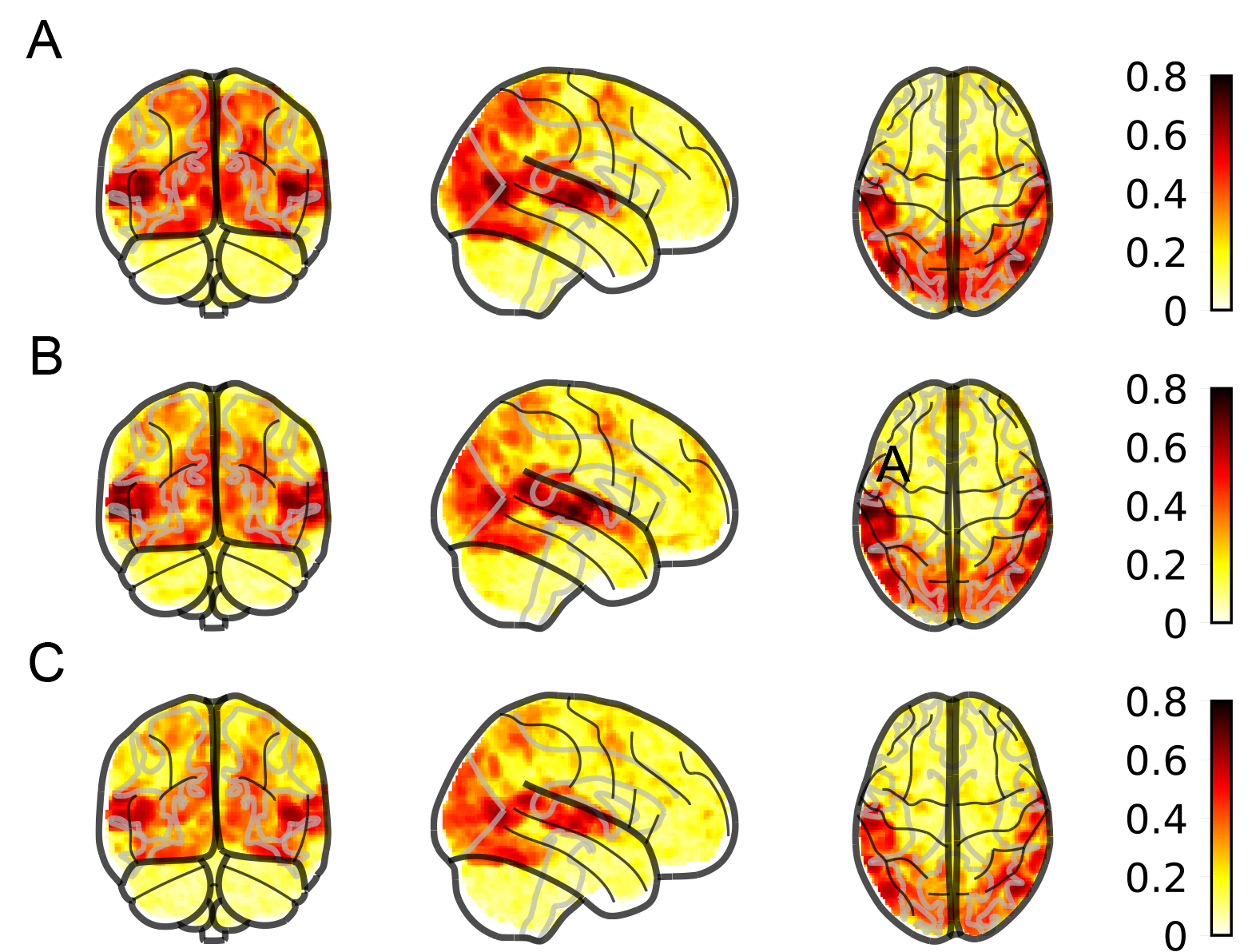

Supplement: S12 Fig — (A) Session 1 vs. session 2. (B) Session 1 vs. session 3, and (C) session 2 vs. session 3. We observed high values for the ISC between the 3 sessions. We also observed similar activation clusters, with similar intensity. Brain plots were generated with nilearn [170]. Pilot III data can be found in the Zenodo release [174]. (TIF) [file pbio.3002797.s013.tif]

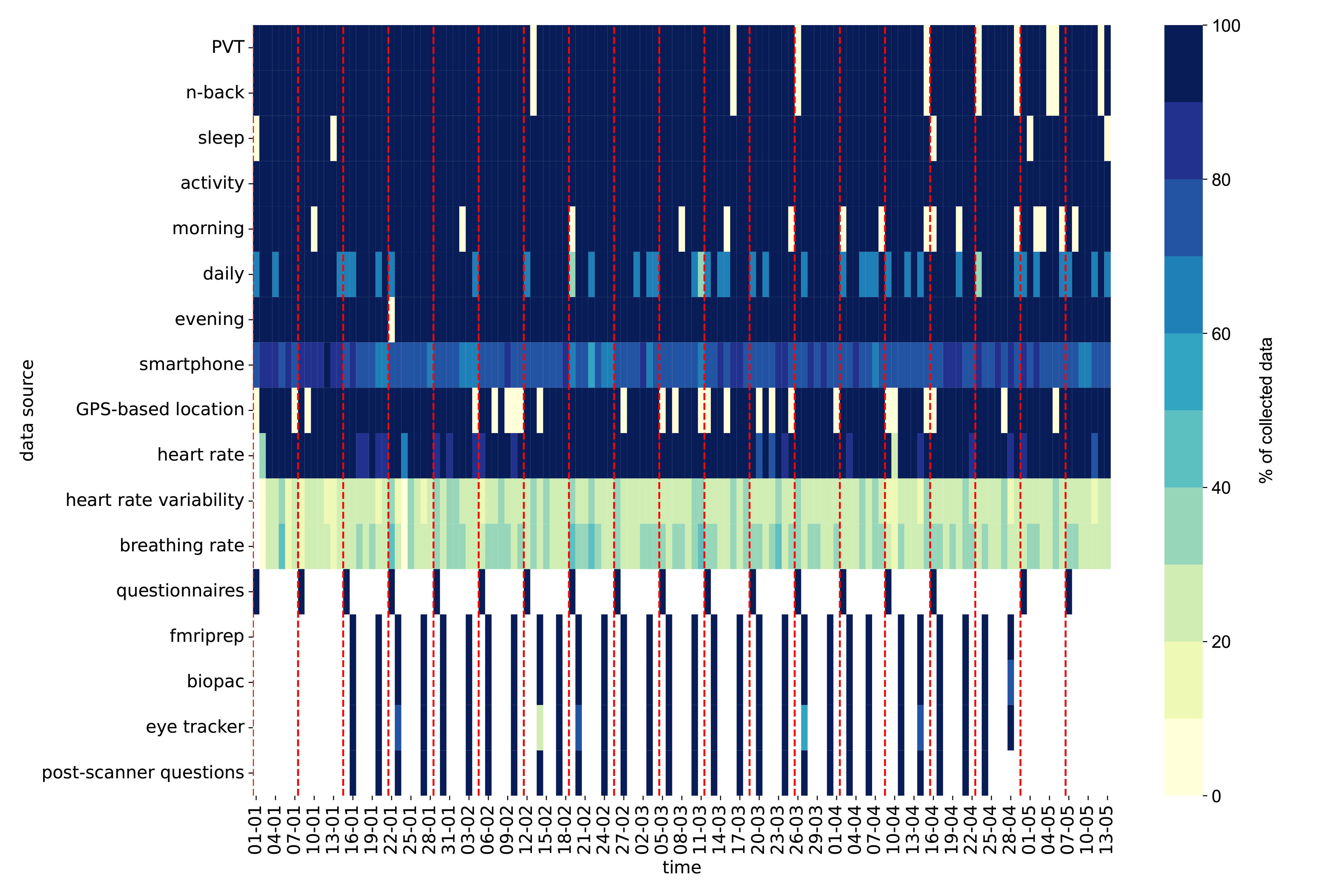

Supplement: S13 Fig — For each day (x-axis), we plotted the percentage of available data for each data source (y-axis). The percentage of available data represents the number of data points that were successfully collected over the total number of data points that were planned to be collected per data source in a day. Some sources provided data only a few days per week, such as the weekly questionnaires that were collected each Sunday (indicated by the red dotted lines), and the MRI sessions, scheduled on Mondays and Fridays. The data set shows minimal missingness, with most of the daily data reaching at least 60% completion, except for pulse rate variability and breathing rate, which have a lower collection rate 20% and 40%. Unprocessed study data can be found in the Zenodo data set release [175]. (TIF) [file pbio.3002797.s014.tif]

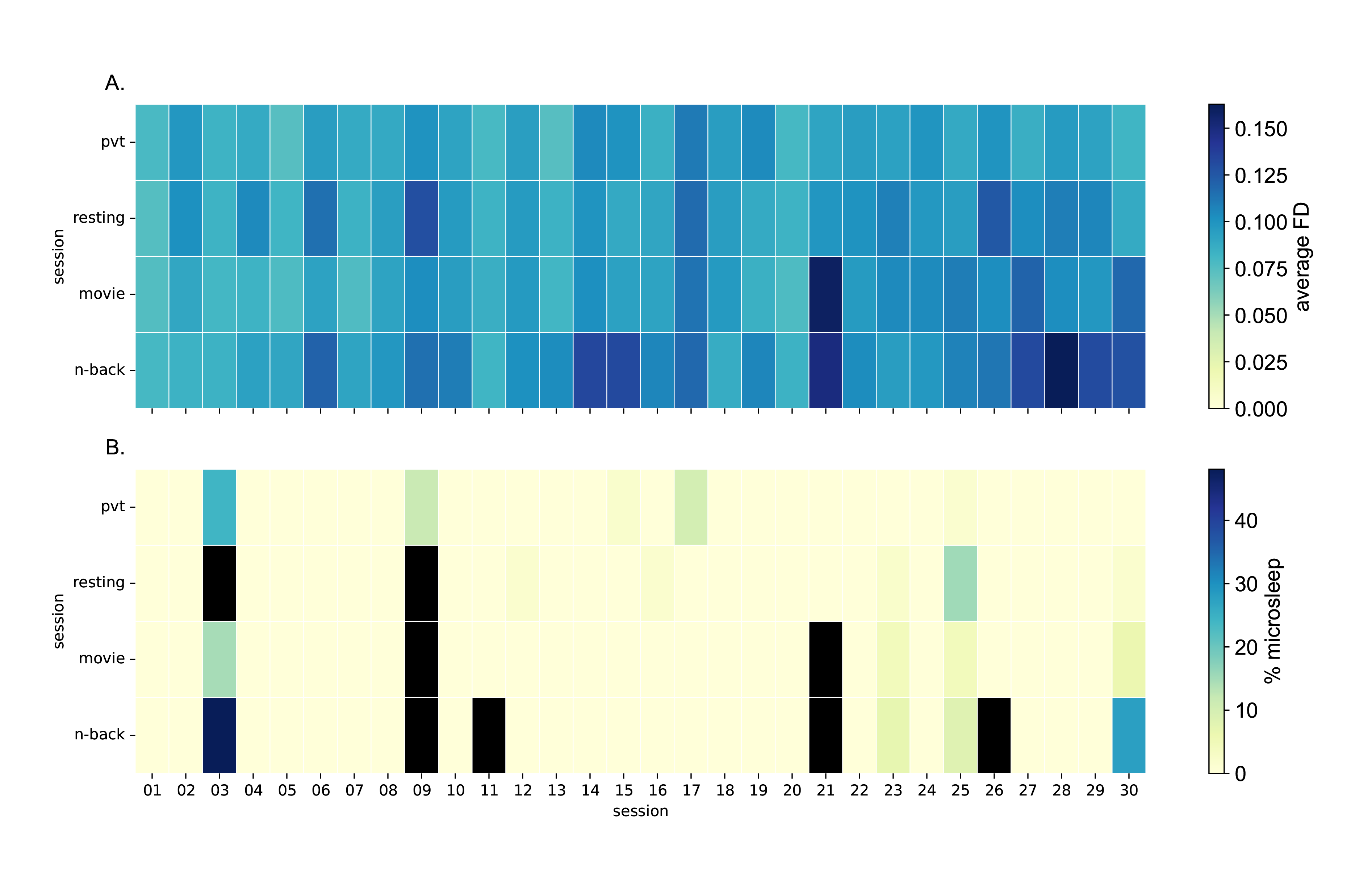

Supplement: S14 Fig — (A) The subject head movement was minimal across most of the sessions and tasks, with mean average framewise displacement (FD) values between 0.07 and 0.17. (B) The subject also remained alert for most of the sessions, only experiencing microsleeps in 18 out of 120 acquisitions. Data from 8 acquisitions was corrupted and could not be used. Unprocessed study data can be found in the Zenodo dataset release [175]. (TIF) [file pbio.3002797.s015.tif]

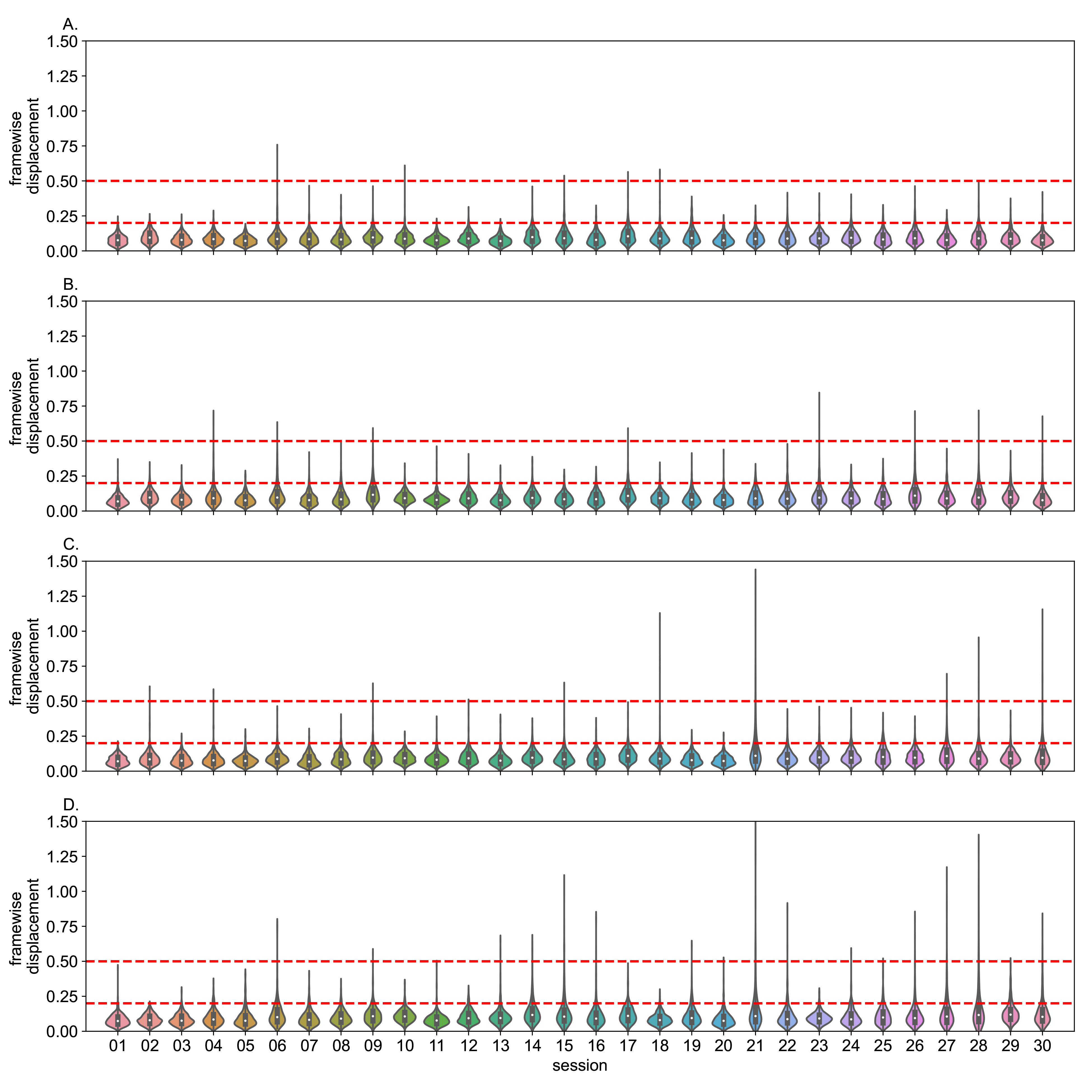

Supplement: S15 Fig — The framewise displacement distributions are shown as violin plots for all sessions during (A) the PVT task, (B) resting state, (C) movie-watching, and (D) the n-back task. The average FD remained under 0.2 for all sessions during all tasks, albeit some high movement peaks. Higher peaks tended to happen during the n-back task. FD data as preprocessed by fmriprep [134,135] are accessible in the GIT repository [176], under the results folder. (TIF) [file pbio.3002797.s016.tif]

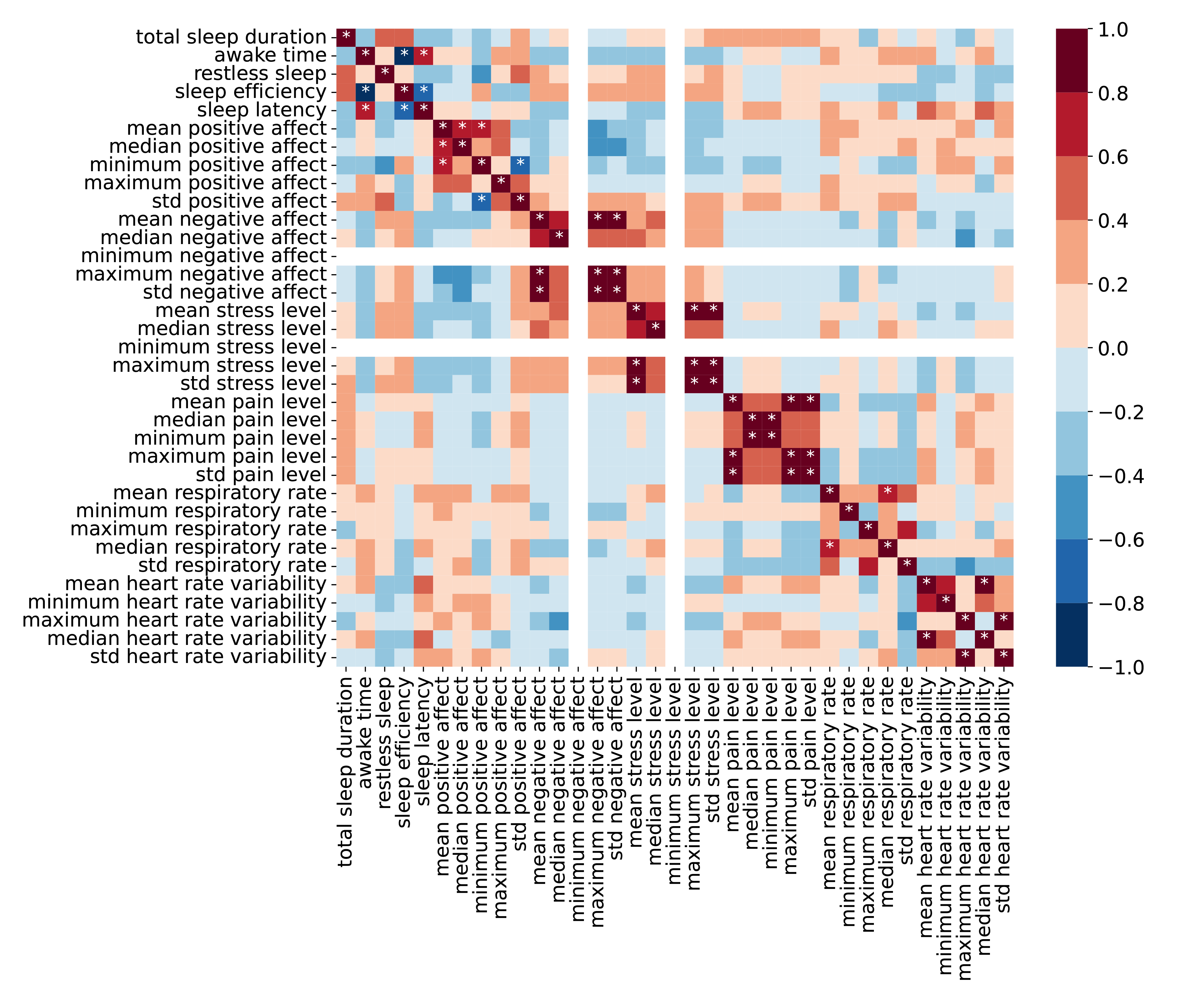

Supplement: S16 Fig — High correlations (ρ > 0.7) are marked with stars. For sleep measurements, high correlations between sleep efficiency and sleep latency are notorious. For mood measurements, high correlations between different statistics on pain levels, negative affects, and stress levels are relevant. In addition, the minimum negative affect and minimum stress levels did not exhibit variations (white stripes). For physiological measurements, we see high correlations between mean respiratory rate and median respiratory rate, maximum respiratory rate and the standard deviation of respiratory rate, mean HRV and median HRV, and maximum HRV and the standard deviation of HRV. Unprocessed study data can be found in the Zenodo dataset release [175]. (TIF) [file pbio.3002797.s017.tif]

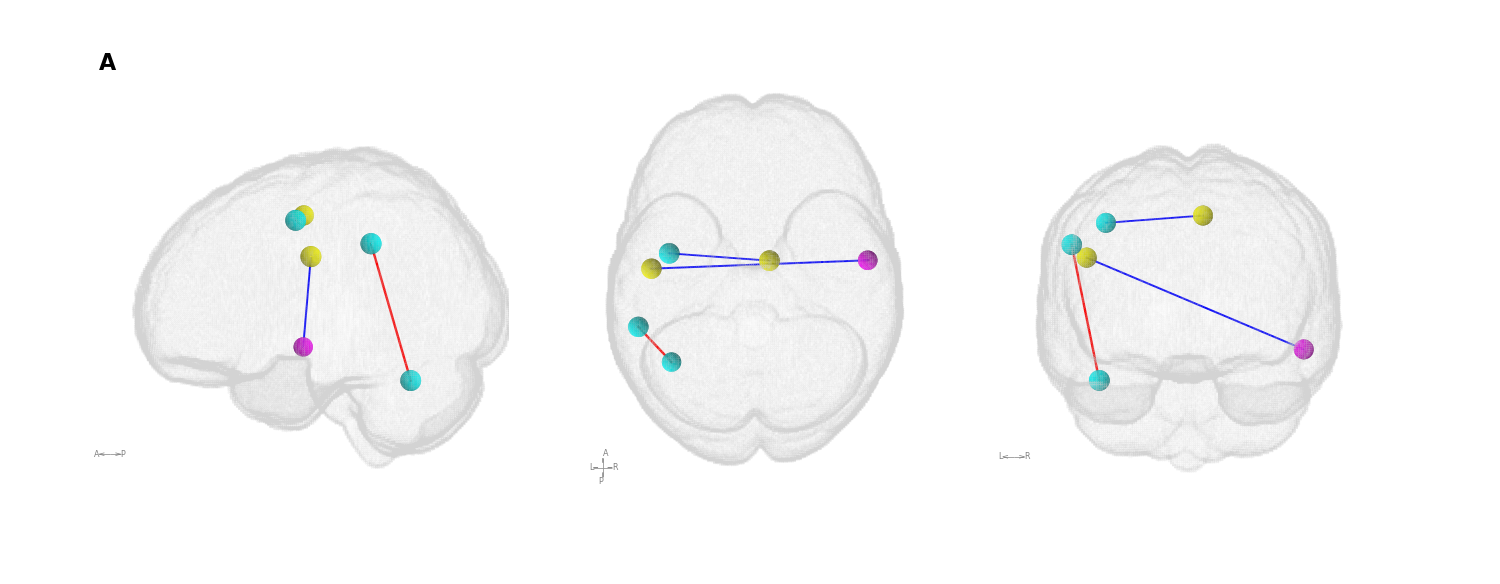

Supplement: S17 Fig — (A) These results are derived using a second parcellation (set2 from Seitzman and colleagues [158]). In this case, restless sleep is associated with connectivity among nodes within the DMN (purple), cingulo-opercular (cyan), and somatomotor (yellow) networks. Red colors indicate positive correlations and blue colors indicate negative correlations. Results are empirically thresholded via 10,000 iterations of nonparametric permutation testing and further corrected for multiple comparisons (corrected p < 0.05). All links are listed in the S5 Table. Brain plots were generated with netplotbrain [171]. Unprocessed study data can be found in the Zenodo dataset release [175]. Processed results derived from the study data are accessible in the GIT repository [176], under the results folder. (TIF) [file pbio.3002797.s018.tif]

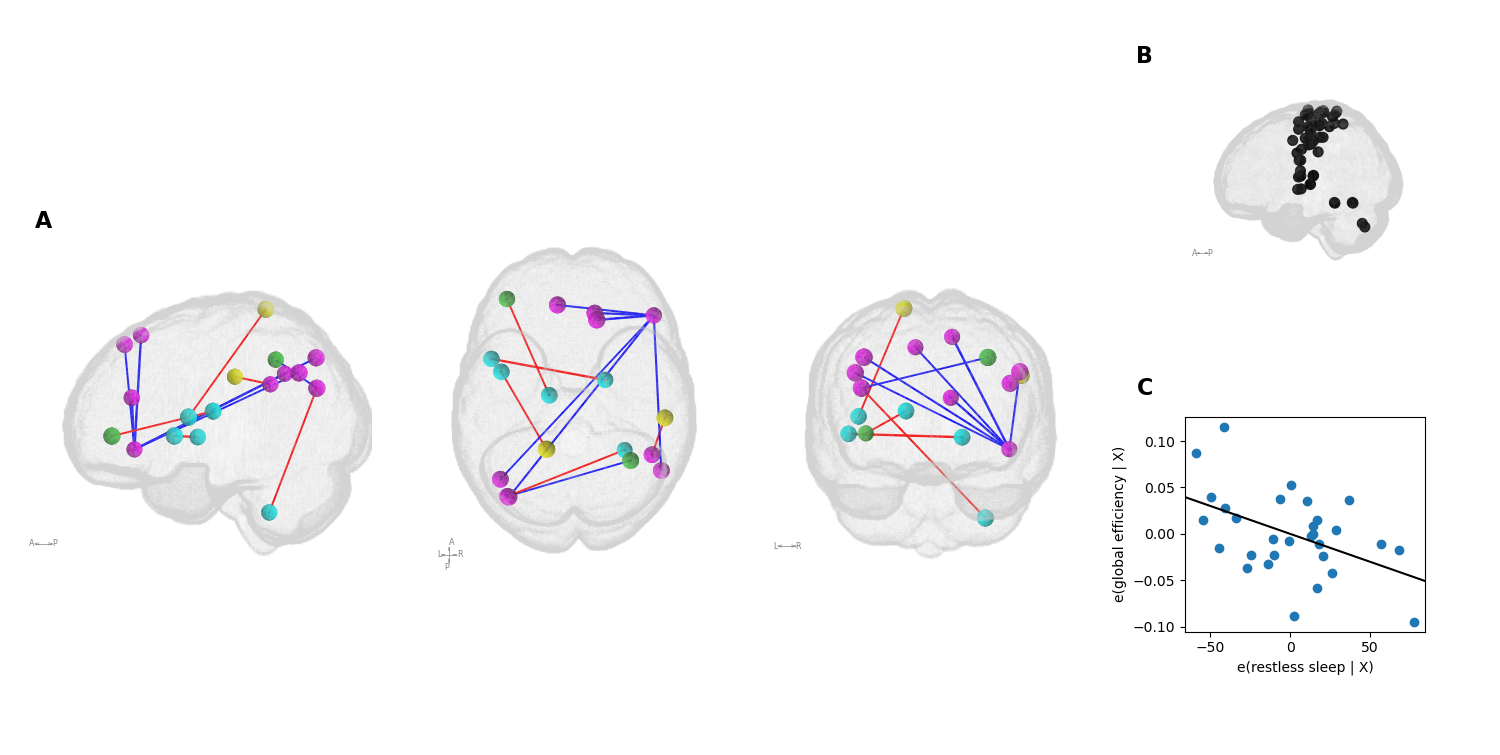

Supplement: S18 Fig — These results are derived by including the global signal as a regressor and using the set1 from Seitzman and colleagues [158]. (A) For this case, restless sleep is associated with connectivity among nodes within the DMN (purple), cingulo-opercular (cyan), fronto-parietal (green), and somatomotor (yellow) networks. Red colors indicate positive correlations and blue colors indicate negative correlations. (B) Nodes from the somatomotor network employed to compute the participation coefficient. (C) Partial regression plot showing that the somatomotor network’s participation coefficient is proportionally related to the previous night’s restless sleep. Results are empirically thresholded via 10,000 iterations of nonparametric permutation testing and further corrected for multiple comparisons (corrected p < 0.05). All links are listed in the S6 Table. Brain plots were generated with netplotbrain [171]. Unprocessed study data can be found in the Zenodo data set release [175]. Processed results derived from the study data are accessible in the GIT repository [176], under the results folder. (TIF) [file pbio.3002797.s019.tif]

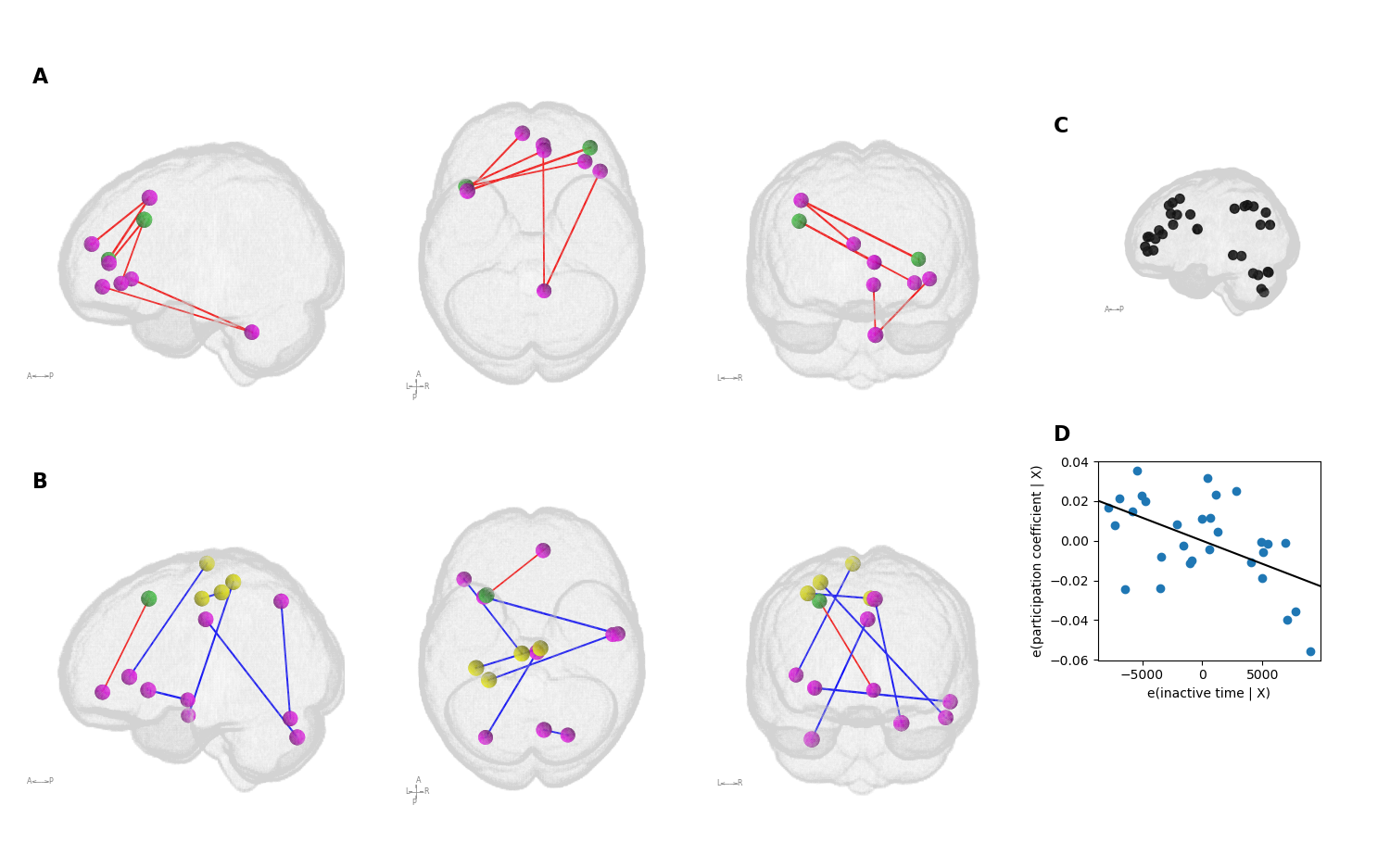

Supplement: S19 Fig — These results are derived using a second parcellation (set2 from Seitzman and colleagues [158]). (A) Linear regression models on individual links showed significant associations between the previous day’s inactive time and links in the DMN (purple), fronto-parietal (green), and somatomotor (yellow) networks. (B) Similarly, analyses revealed significant relationships between the prior night’s restless sleep and these same networks. Red colors indicate positive correlations and blue colors indicate negative correlations. (C) Nodes from the fronto-parietal network employed to compute the participation coefficient. (D) Partial regression plot showing that the fronto-parietal network’s participation coefficient is proportionally related to the previous day’s inactive time. Results are empirically thresholded via 10,000 iterations of non-parametric permutation testing and further corrected for multiple comparisons (corrected p < 0.05). All links are listed in the S9 Table. Brain plots were generated with netplotbrain [171]. Unprocessed study data can be found in the Zenodo data set release [175]. Processed results derived from the study data are accessible in the GIT repository [176], under the results folder. (TIF) [file pbio.3002797.s020.tif]

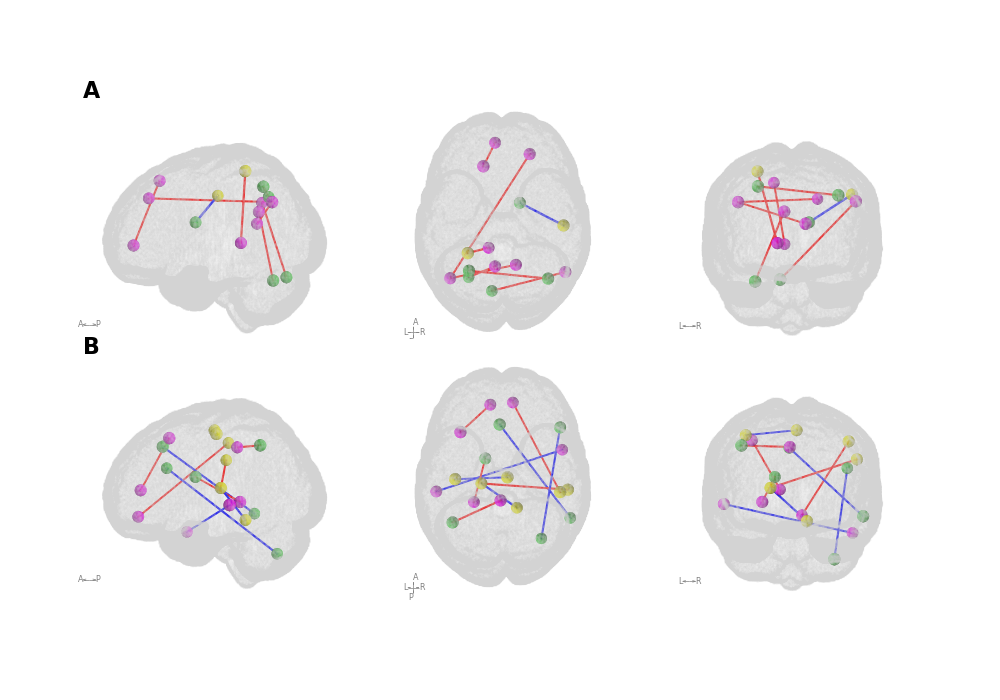

Supplement: S20 Fig — These results are derived by including the global signal as a regressor and using the set1 from Seitzman and colleagues [158]. (A) Linear regression models on individual links showed significant associations between the previous day’s inactive time and links in the DMN (purple), fronto-parietal (green), and somatomotor (yellow) networks. (B) Similarly, analyses revealed significant relationships between the prior night’s restless sleep and these same networks. Red colors indicate positive correlations and blue colors indicate negative correlations. Results are empirically thresholded via 10,000 iterations of nonparametric permutation testing and further corrected for multiple comparisons (corrected p < 0.05). All links are listed in the S10 Table. Brain plots were generated with netplotbrain [171]. Unprocessed study data can be found in the Zenodo data set release [175]. Processed results derived from the study data are accessible in the GIT repository [176], under the results folder. (TIF) [file pbio.3002797.s021.tif]

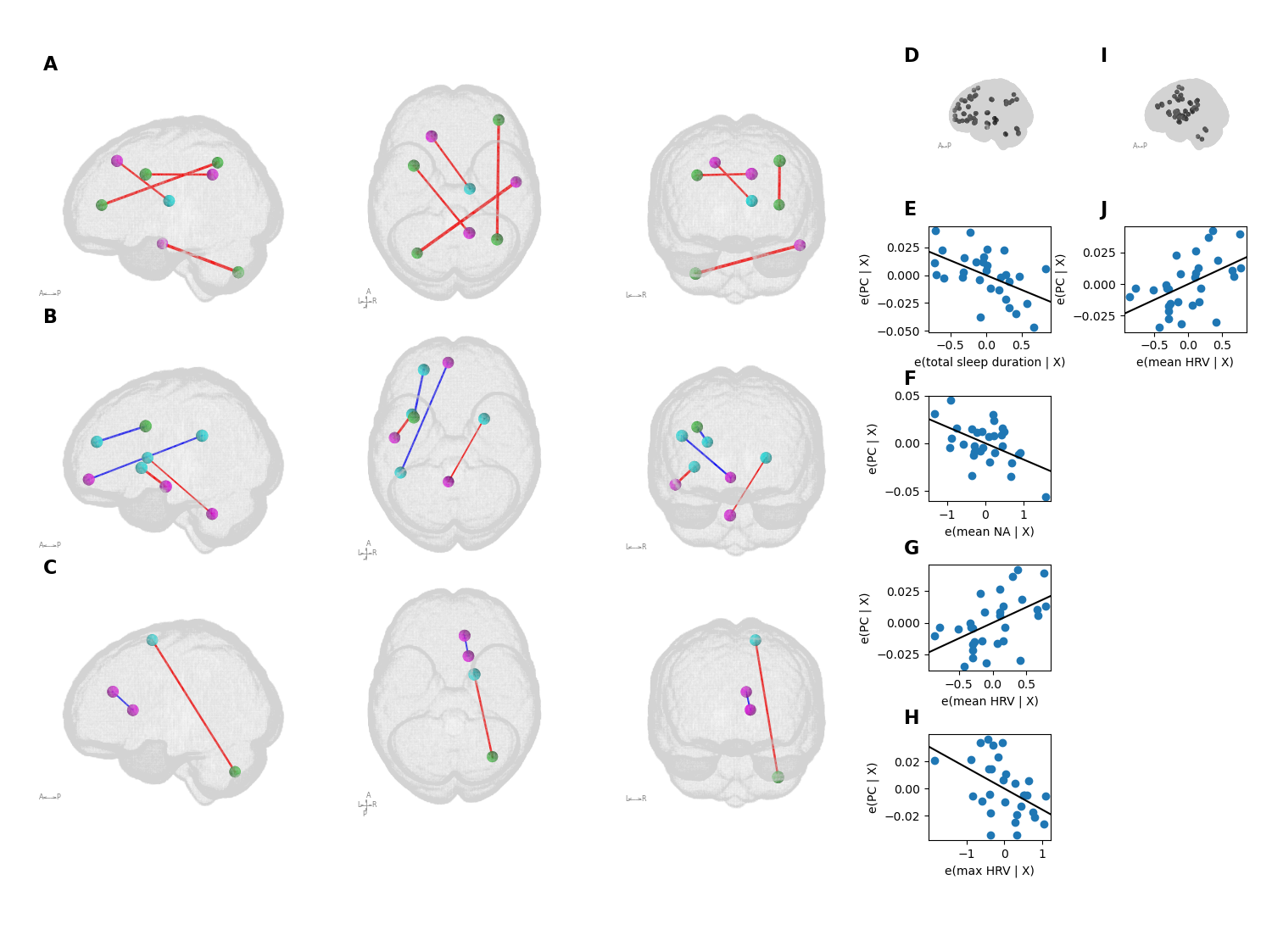

Supplement: S21 Fig — These results are derived using a second parcellation (set2 from Seitzman and colleagues [158]). (A) Linear regression models on individual links showed significant associations between the previous day’s awake time in bed and the connectivity between the cingulo-opercular network (cyan), FPN(green), and the DMN (purple). (B) Analyses also revealed significant relationships between the microsleep time in the scanner and the resting-state connectivity in the DMN, cingulo-opercular, and fronto-parietal networks. (C) Similarly, regression analysis demonstrated a direct proportional relationship between prior night’s maximum heart rate variability and the connectivity between links in the DMN, FPN, and cingulo-opercular network. Red colors indicate positive correlations and blue colors indicate negative correlations. (D) Nodes from the DMN employed to compute the network’s participation coefficient. Partial regression plots showing that the DMN participation coefficient is strongly predicted by the previous day’s (E) total sleep duration, (F) mean negative affect, (G) mean heart rate variability, and (H) maximum heart rate variability. (I) Nodes from the cingulo-opercular network employed to compute the network’s participation coefficient. (J) Partial regression plot showing that the CON participation coefficient is strongly predicted by the previous day’s mean heart rate variability. Results are empirically thresholded via 10,000 iterations of nonparametric permutation testing and further corrected for multiple comparisons (corrected p < 0.05). All links are listed in the S13 Table. Brain plots were generated with netplotbrain [171]. Unprocessed study data can be found in the Zenodo data set release [175]. Processed results derived from the study data are accessible in the GIT repository [176], under the results folder. (TIF) [file pbio.3002797.s022.tif]

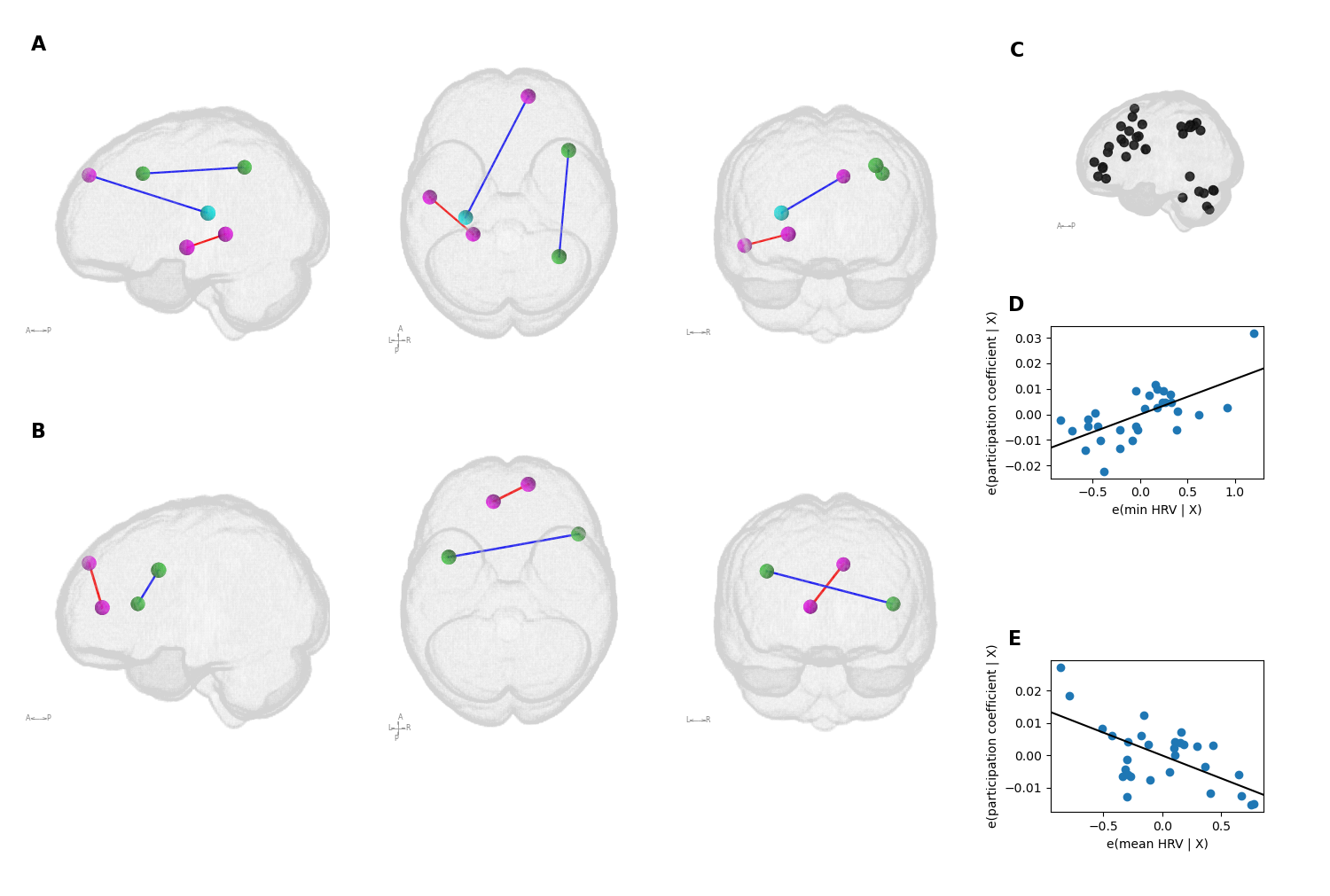

Supplement: S22 Fig — These results are derived by including the global signal as a regressor and using the set1 from Seitzman and colleagues [158]. (A) Linear regression models on individual links showed significant associations between the prior night’s maximum heart rate variability and the connectivity between the cingulo-opercular network (cyan), FPN(green), and the DMN (purple). (B) Analyses also revealed significant relationships between the microsleep time in the scanner and the resting-state connectivity in the DMN, cingulo-opercular, and fronto-parietal networks. (C) Nodes from the FPN employed to compute the network’s participation coefficient. Partial regression plots showing that the FPN participation coefficient is strongly predicted by the previous day’s (D) minimum heart rate variability, (F) mean heart rate variability. Results are empirically thresholded via 10,000 iterations of nonparametric permutation testing and further corrected for multiple comparisons (corrected p < 0.05). All links are listed in the S14 Table. Results for the participation coefficient are listed in the S15 Table. Brain plots were generated with netplotbrain [171]. Unprocessed study data can be found in the Zenodo data set release [175]. Processed results derived from the study data are accessible in the GIT repository [176], under the results folder. (TIF) [file pbio.3002797.s023.tif]

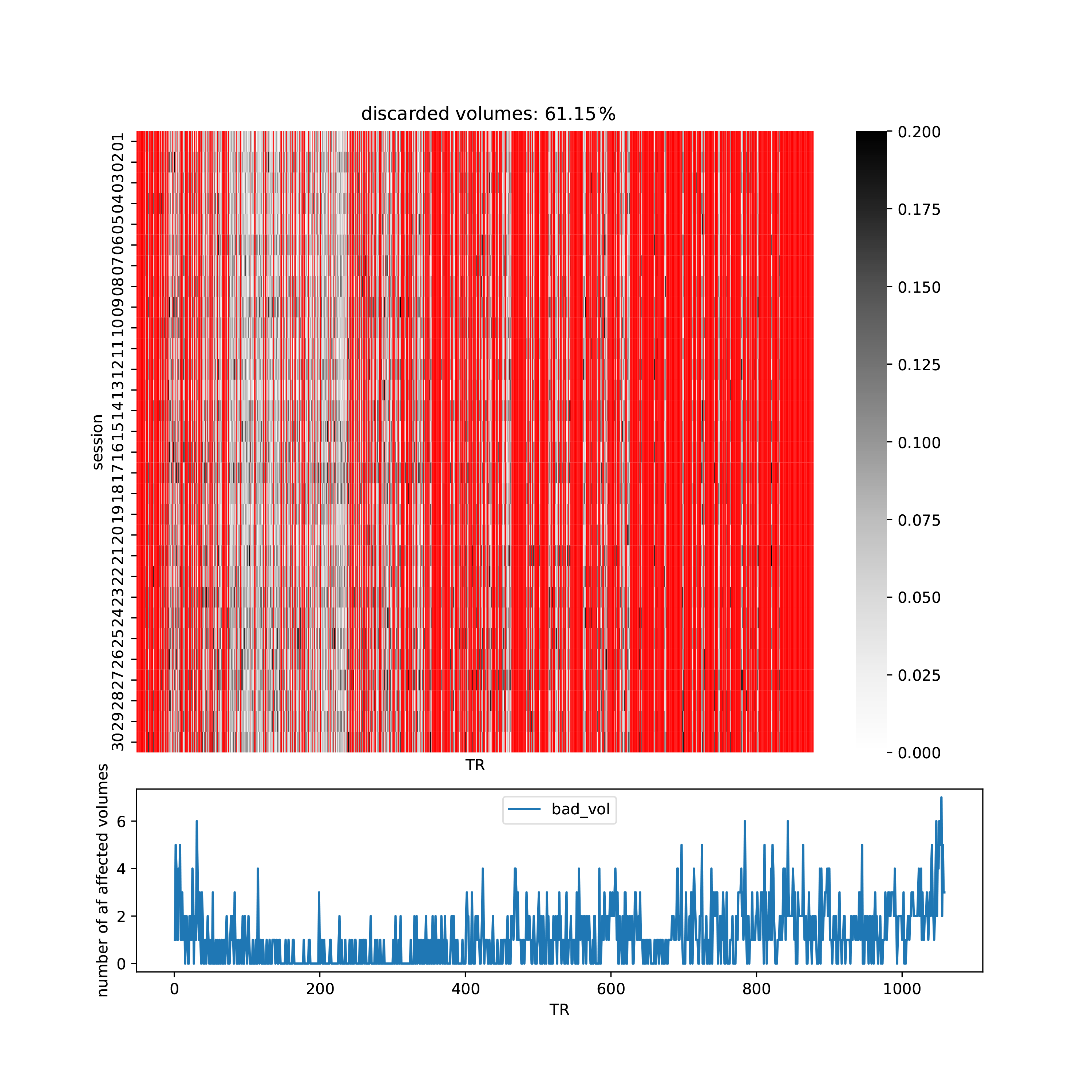

Supplement: S23 Fig — Because the ISC computations require fMRI data with equal number of volumes, ordinary scrubbing requires detecting all volumes with high movement (FD > 0.2) in each session and censoring them across all sessions. Applying this scrubbing technique censors 60.11% of the volumes for all sessions. We plotted the (A) volumes to be censored using ordinary scrubbing for the 30 sessions. Each TR volume is presented in the x-axis, while sessions are shown in the y-axis. Gray colors show the FD and censored volumes are in red. (B) We also plot the number of sessions that exceeded the FD threshold. Unprocessed study data can be found in the Zenodo data set release [175]. Processed results derived from the study data are accessible in the GIT repository [176], under the results folder. (TIF) [file pbio.3002797.s024.tif]

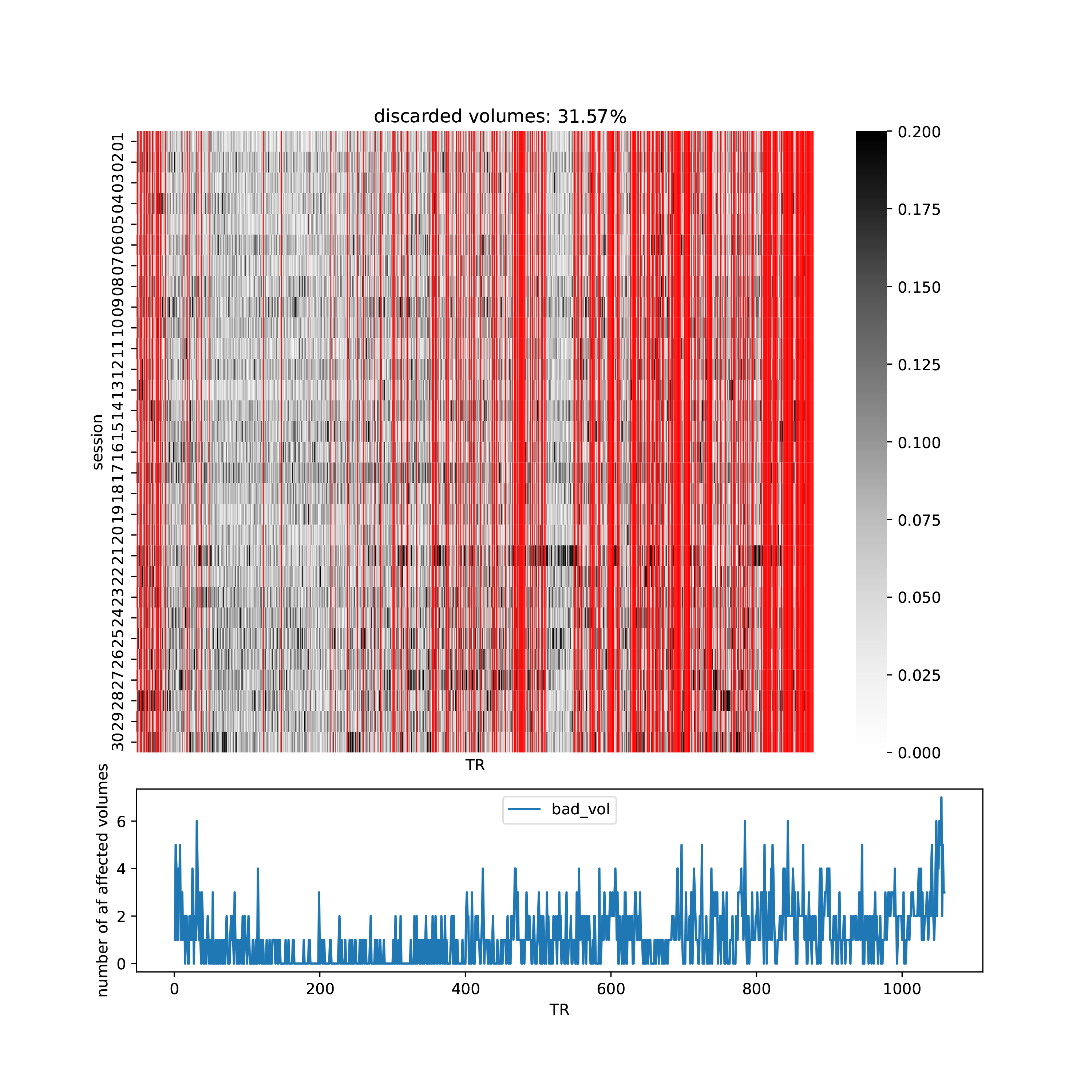

Supplement: S24 Fig — For each volume, percentage scrubbing involves detecting the total number of sessions (ns) with high movement (FD > 0.2) and censoring the volume across all sessions only if ns is above a threshold. This threshold is usually related to a percentage of the sessions. Applying this scrubbing technique censors 31.47% of the volumes for all sessions. We plotted the (A) volumes to be censored using percentage scrubbing at 5% (i.e., minimum 2 sessions with high movement). Each TR volume is presented in the x-axis, while sessions are shown in the y-axis. Gray colors show the FD and censored volumes are in red. (B) We also plot the number of sessions that exceeded the FD threshold. Unprocessed study data can be found in the Zenodo data set release [175]. Processed results derived from the study data are accessible in the GIT repository [176], under the results folder. (TIF) [file pbio.3002797.s025.tif]

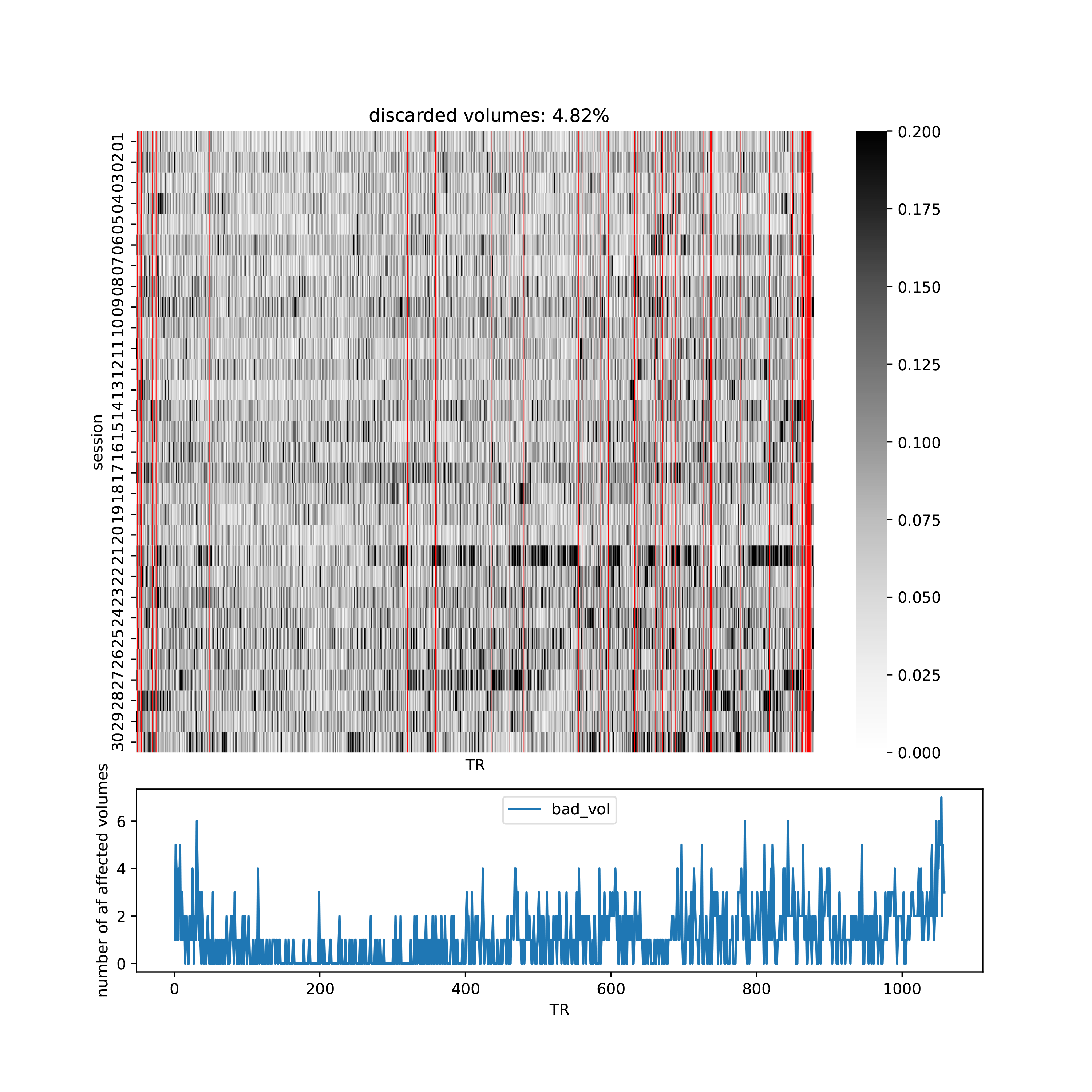

Supplement: S25 Fig — For each volume, percentage scrubbing involves detecting the total number of sessions (ns) with high movement (FD > 0.2) and censoring the volume across all sessions only if ns is above a threshold. This threshold is usually related to a percentage of the sessions. Applying this scrubbing technique censors 4.63% of the volumes for all sessions. We plotted the (A) volumes to be censored using percentage scrubbing at 10% (i.e., minimum 3 sessions with high movement). Each TR volume is presented in the x-axis, while sessions are shown in the y-axis. Gray colors show the FD and censored volumes are in red. (B) We also plot the number of sessions that exceeded the FD threshold. Unprocessed study data can be found in the Zenodo data set release [175]. Processed results derived from the study data are accessible in the GIT repository [176], under the results folder. (TIF) [file pbio.3002797.s026.tif]

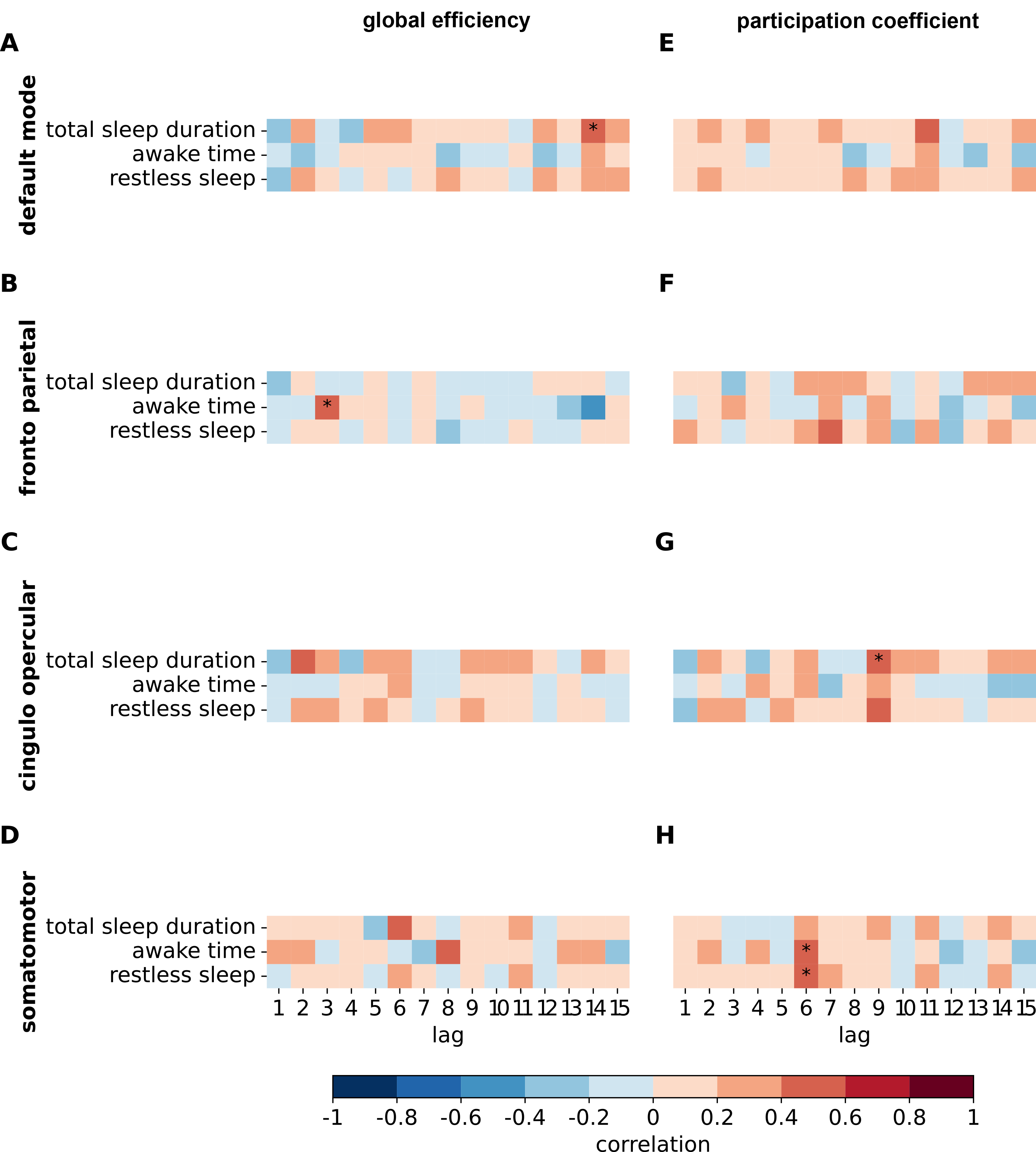

Supplement: S26 Fig — These results are derived by thresholding the network at 20% proportional threshold. Sleep patterns from 3 and 14 days in the past are correlated with the global efficiency in the (A) DMN, and (B) FPN. No significant correlations are found for sleep patterns and (C) CON, and the (D) somatomotor network. Similarly, no correlation patterns are found for the participation coefficient of the (E) DMN or (F) FPN. Nevertheless, sleep patterns from 6 to 9 days in the past are correlated with the participation coefficient in the (G) CON, and (H) somatomotor network. Significant correlations are shown by an asterisk (*). Unprocessed study data can be found in the Zenodo data set release [175]. Processed results derived from the study data are accessible in the GIT repository [176], under the results folder. (TIF) [file pbio.3002797.s027.tif]

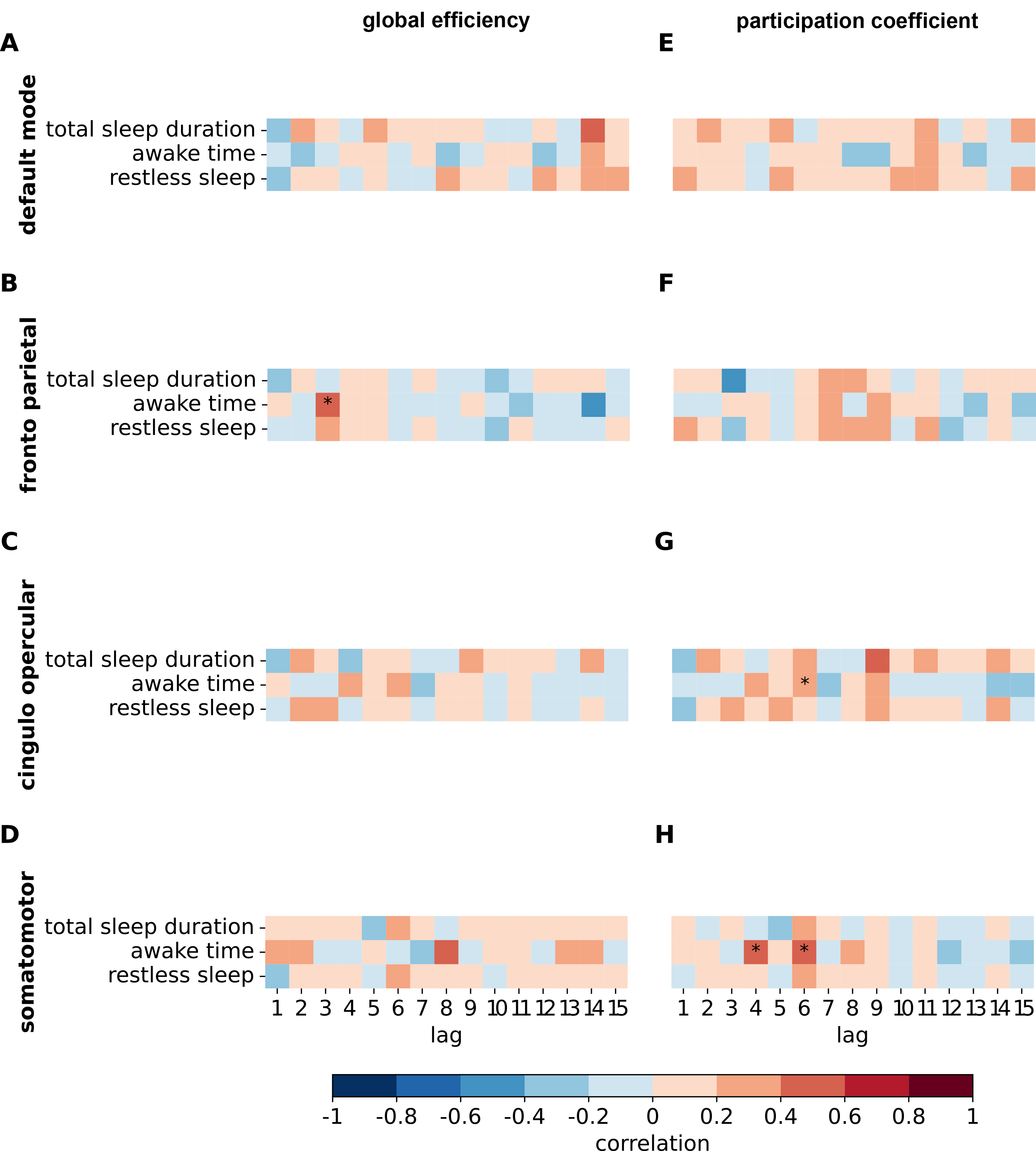

Supplement: S27 Fig — These results are derived by thresholding the network at 30% proportional threshold. Sleep patterns from 3 days in the past are correlated with the global efficiency in the (B) FPN. No significant correlations are found for sleep patterns and the (A) DMN, (C) CON, and the (D) somatomotor network. Similarly, no correlation patterns are found for the participation coefficient of the (E) DMN or (F) FPN. Nevertheless, sleep patterns from 4 to 6 days in the past are correlated with the participation coefficient in the (G) CON, and (H) somatomotor network. Significant correlations are shown by an asterisk (*). Unprocessed study data can be found in the Zenodo data set release [175]. Processed results derived from the study data are accessible in the GIT repository [176], under the results folder. (TIF) [file pbio.3002797.s028.tif]

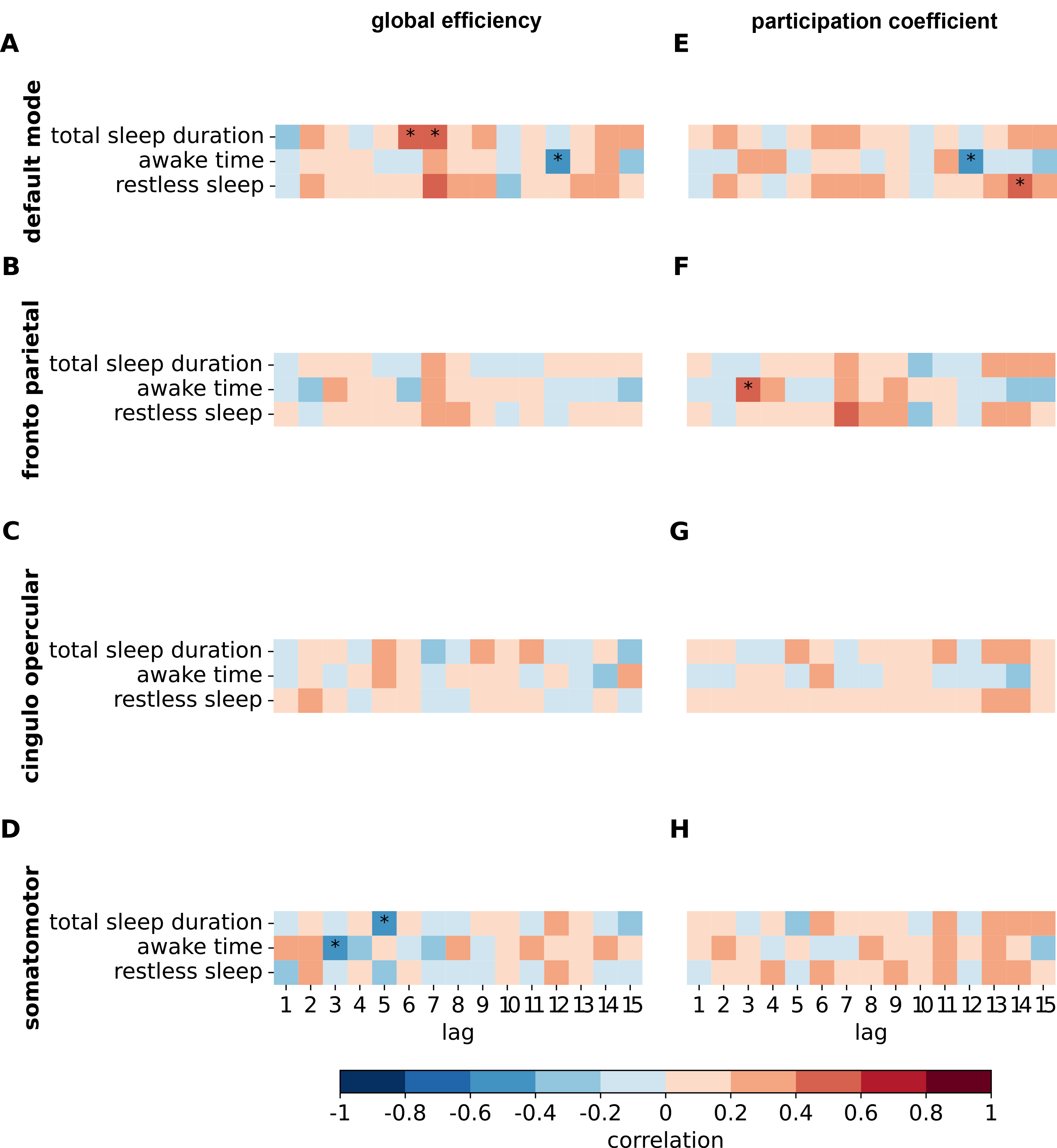

Supplement: S28 Fig — These results are derived by using the set2 from Seitzman and colleagues [158] with 10% proportional threshold. Sleep patterns from 3 to 12 days in the past are correlated with the global efficiency in the (A) DMN and the (D) somatomotor network. No significant correlations are found for sleep patterns and the (B) FPN, and (C) CON. Similarly, sleep patterns from 3 to 14 days in the past are correlated with the participation coefficient in the (E) DMN and (F) FPN. No correlation patterns are found for the participation coefficient of the (G) CON or (H) somatomotor network. Significant correlations are shown by an asterisk (*). Unprocessed study data can be found in the Zenodo data set release [175]. Processed results derived from the study data are accessible in the GIT repository [176], under the results folder. (TIF) [file pbio.3002797.s029.tif]

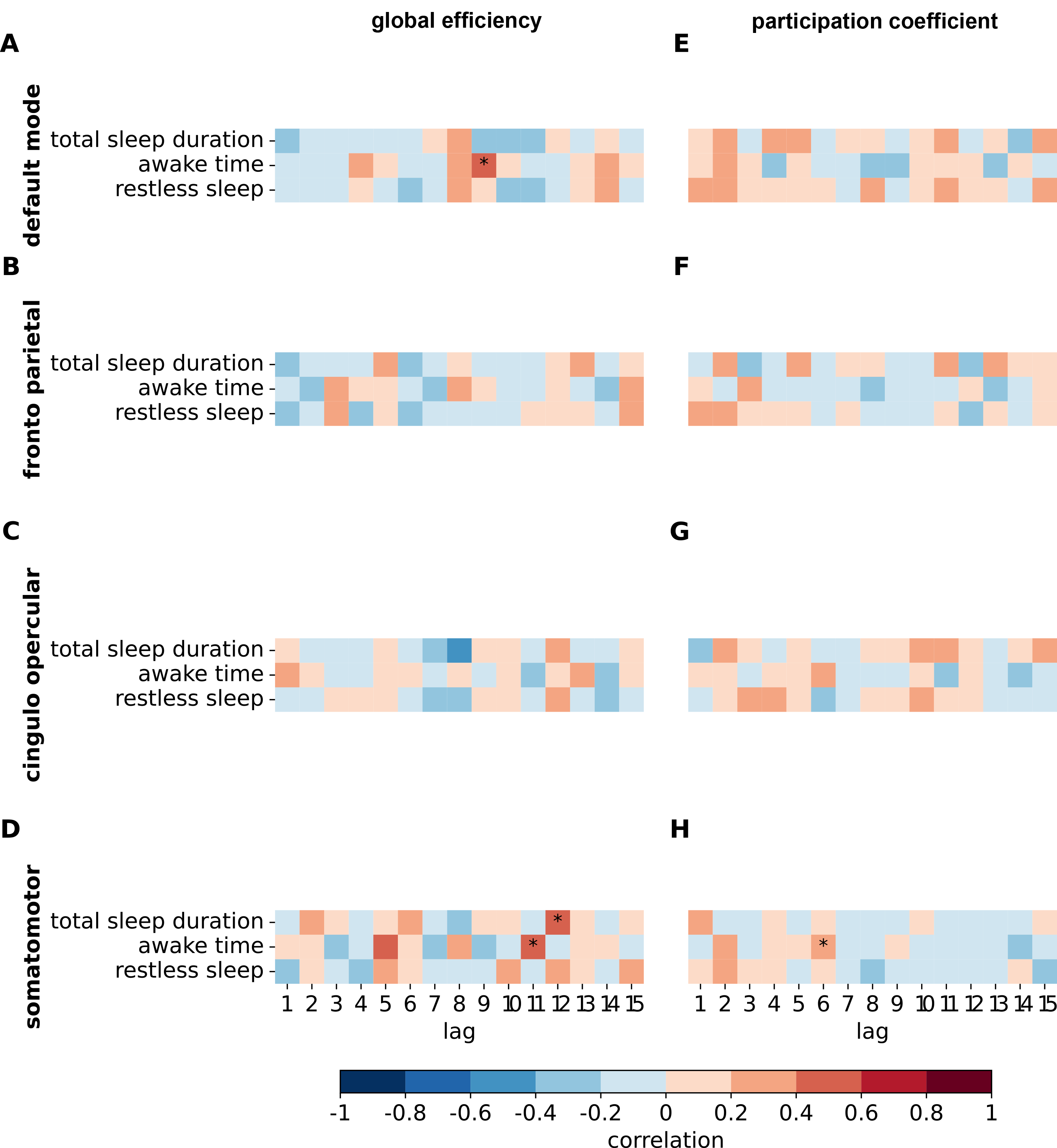

Supplement: S29 Fig — These results are derived by including the global signal as a regressor and using the set1 from Seitzman and colleagues [158] with 10% proportional threshold. Sleep patterns from 9 to 12 days in the past are correlated with the global efficiency in the (A) DMN and the (D) somatomotor network. No significant correlations are found for sleep patterns and the (B) FPN, and (C) CON. Similarly, no correlation patterns are found for the participation coefficient of the (E) DMN, (F) FPN, or (G) CON. Nevertheless, sleep patterns from 6 days in the past are correlated with the participation coefficient in the (H) somatomotor network. Significant correlations are shown by an asterisk (*). Unprocessed study data can be found in the Zenodo data set release [175]. Processed results derived from the study data are accessible in the GIT repository [176], under the results folder. (TIF) [file pbio.3002797.s030.tif]

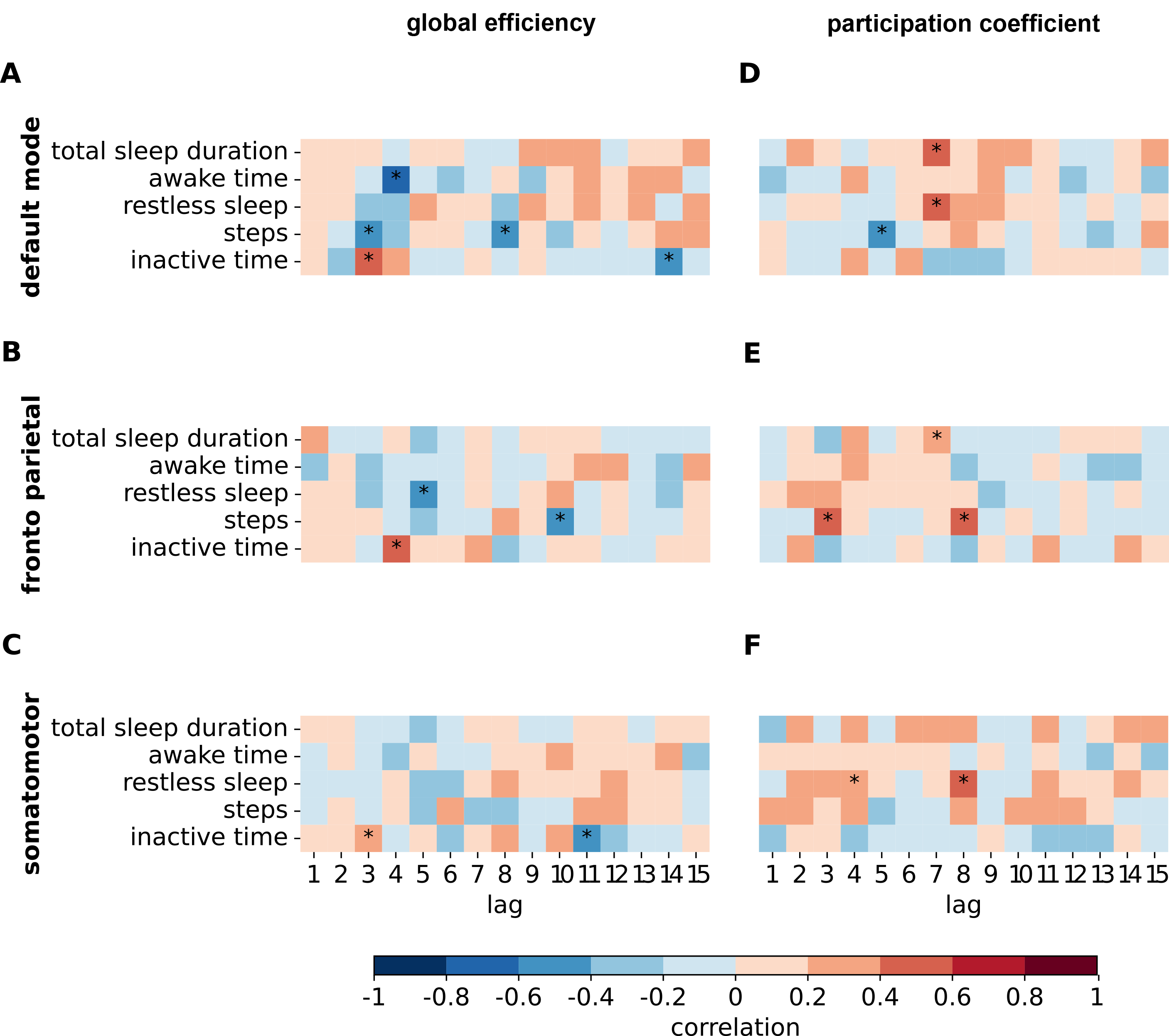

Supplement: S30 Fig — These results are derived by thresholding the network at 20% proportional threshold. Sleep and activity patterns from 3 to 14 days in the past are correlated with the global efficiency in the (A) DMN, (B) FPN, and (C) somatomotor networks. Similarly, sleep and activity patterns from 3 to 8 days in the past are correlated with the participation coefficient in the (D) DMN, (E) FPN, and (F) somatomotor networks. Significant correlations are shown by an asterisk (*). Unprocessed study data can be found in the Zenodo data set release [175]. Processed results derived from the study data are accessible in the GIT repository [176], under the results folder. (TIF) [file pbio.3002797.s031.tif]

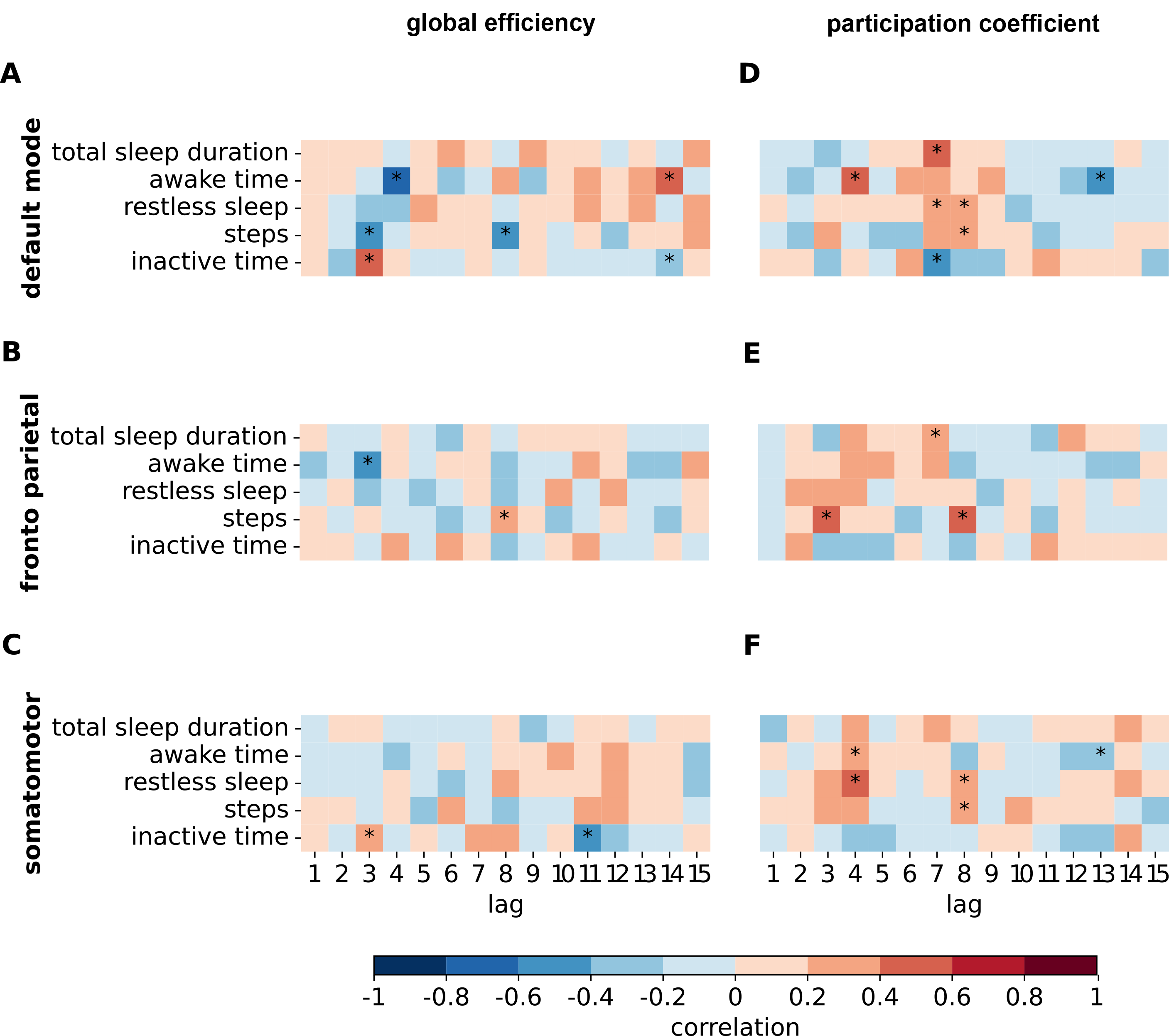

Supplement: S31 Fig — These results are derived by thresholding the network at 30% proportional threshold. Sleep and activity patterns from 3 to 14 days in the past are correlated with the global efficiency in the (A) DMN, (B) FPN, and (C) somatomotor networks. Similarly, sleep and activity patterns from 3 to 13 days in the past are correlated with the participation coefficient in the (D) DMN, (E) FPN, and (F) somatomotor networks. Significant correlations are shown by an asterisk (*). Unprocessed study data can be found in the Zenodo data set release [175]. Processed results derived from the study data are accessible in the GIT repository [176], under the results folder. (TIF) [file pbio.3002797.s032.tif]

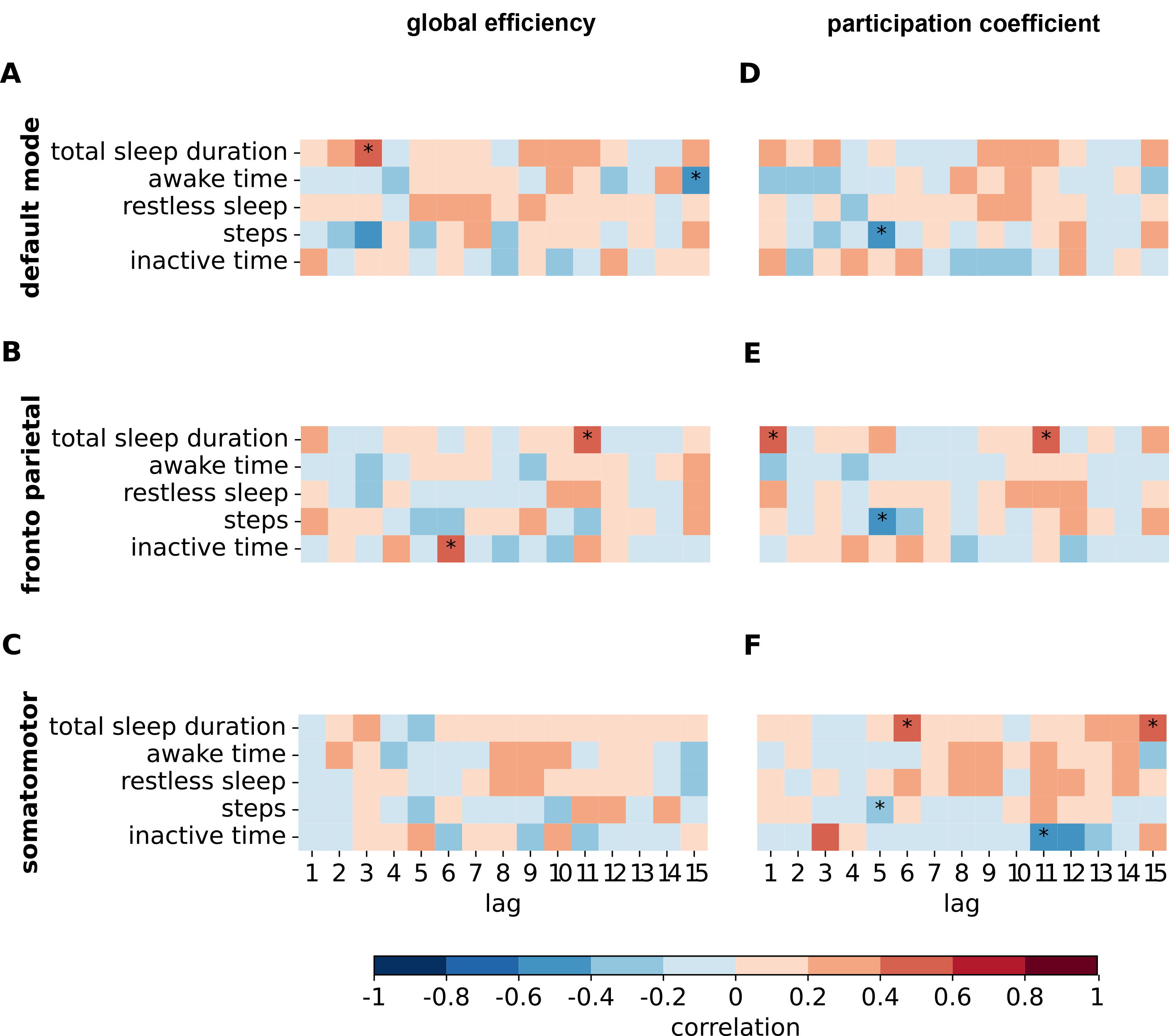

Supplement: S32 Fig — These results are derived by using the set2 from Seitzman and colleagues [158] with 10% proportional threshold. Sleep and activity patterns from 3 to 15 days in the past are correlated with the global efficiency in the (A) DMN, and the (B) FPN. No results are found for the (C) somatomotor network. Similarly, sleep and activity patterns from 1 to 15 days in the past are correlated with the participation coefficient in the (D) DMN, (E) FPN, and (F) somatomotor networks. Significant correlations are shown by an asterisk (*). Unprocessed study data can be found in the Zenodo data set release [175]. Processed results derived from the study data are accessible in the GIT repository [176], under the results folder. (TIF) [file pbio.3002797.s033.tif]

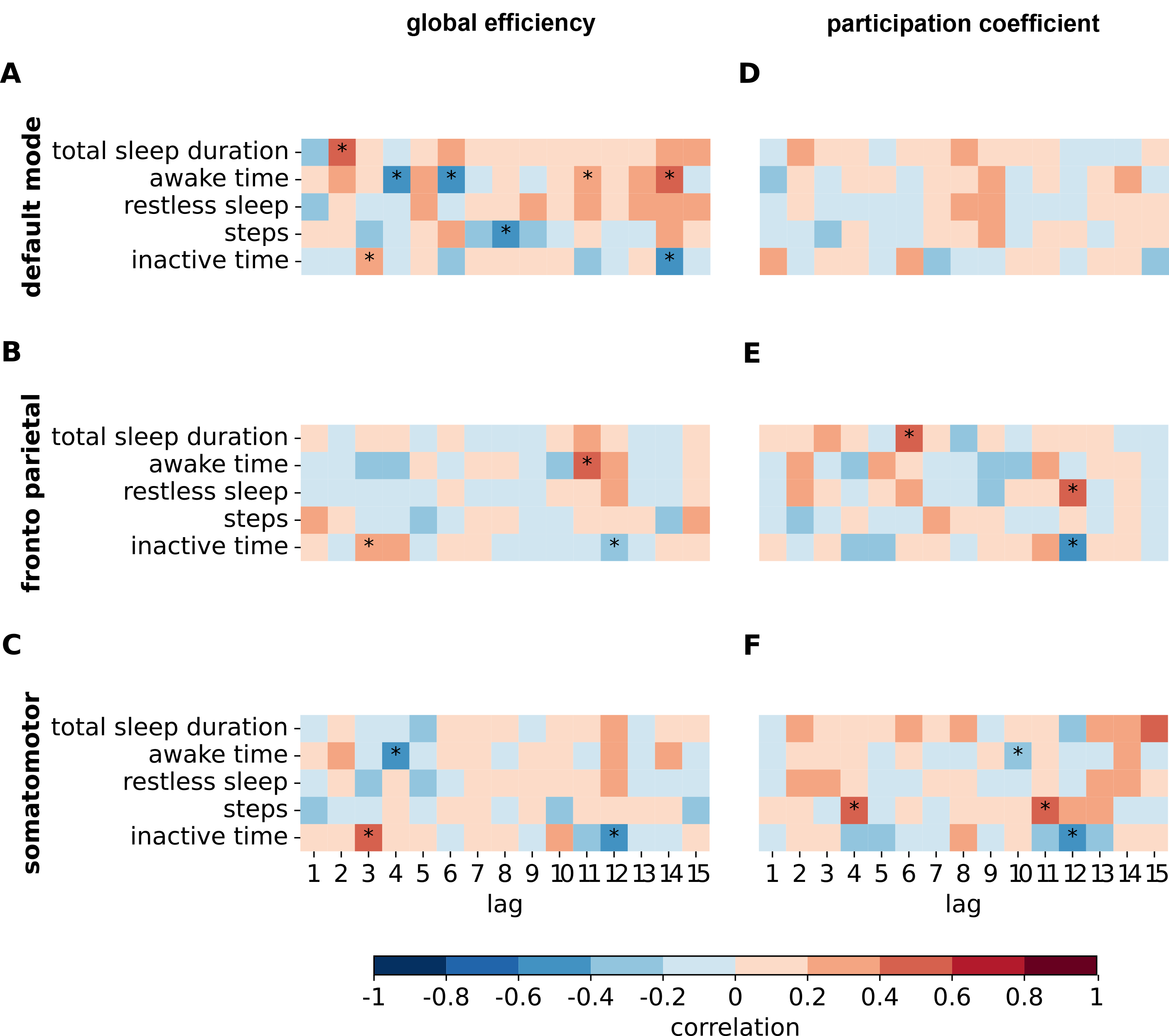

Supplement: S33 Fig — These results are derived by including the global signal as a regressor and using the set1 from Seitzman and colleagues [158] with 10% proportional threshold. Sleep and activity patterns from 2 to 14 days in the past are correlated with the global efficiency in the (A) DMN, (B) FPN, and (C) somatomotor networks. Similarly, sleep and activity patterns from 4 to 12 days in the past are correlated with the participation coefficient in the (E) FPN, and (F) somatomotor networks. No significant results are found for the (D) DMN. Significant correlations are shown by an asterisk (*). Unprocessed study data can be found in the Zenodo data set release [175]. Processed results derived from the study data are accessible in the GIT repository [176], under the results folder. (TIF) [file pbio.3002797.s034.tif]

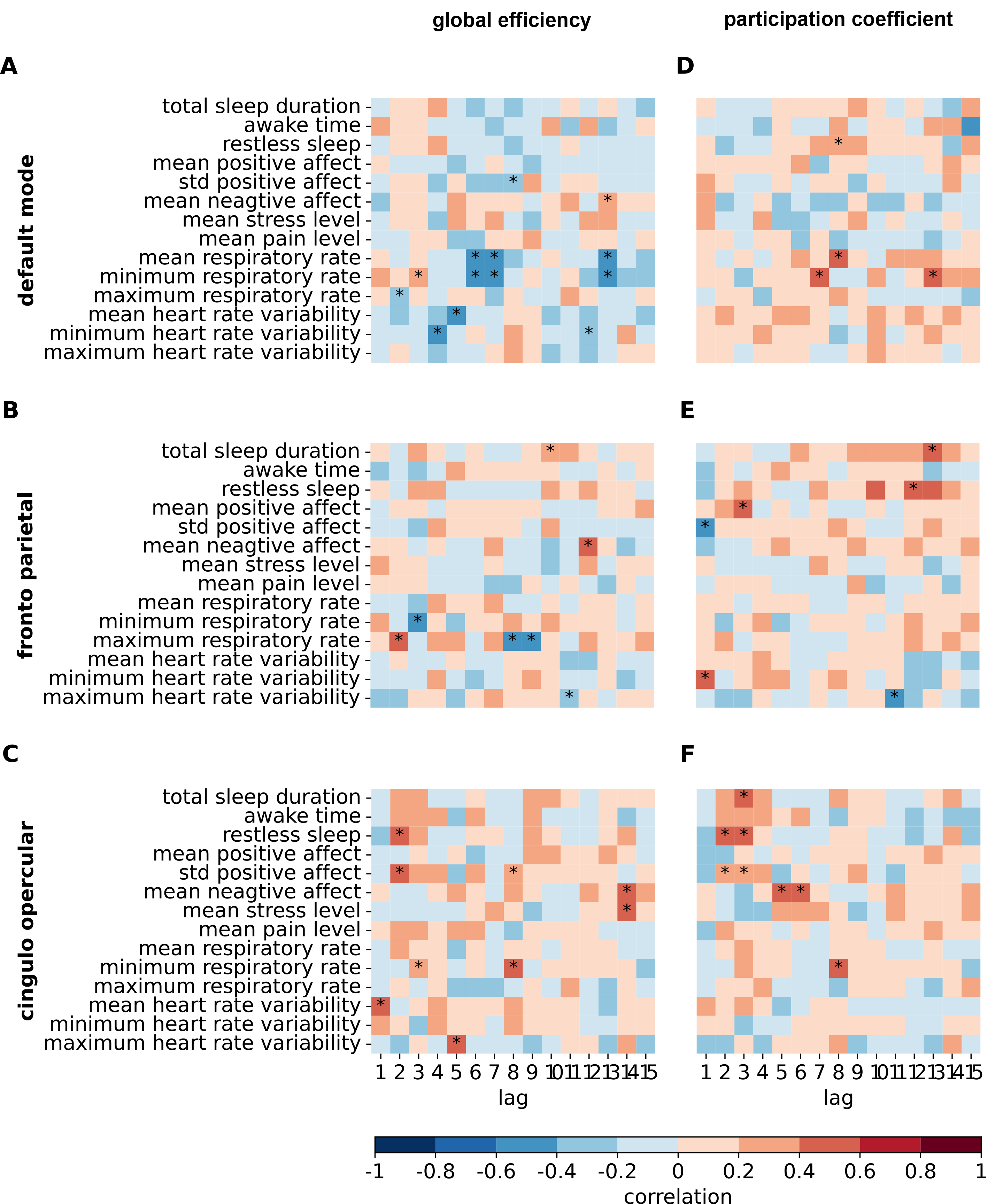

Supplement: S34 Fig — These results are derived by thresholding the network at 20% proportional threshold. Patterns from the previous day to 14 days in the past are correlated with the global efficiency in the (A) DMN, (B) FPN, and (C) CON. Similarly, sleep, activity and ANS activity patterns from the previous day up to 13 days in the past are correlated with the participation coefficient in the (D) DMN, (E)FPN, and (F) CON. Significant correlations are shown by an asterisk (*). Unprocessed study data can be found in the Zenodo data set release [175]. Processed results derived from the study data are accessible in the GIT repository [176], under the results folder. (TIF) [file pbio.3002797.s035.tif]

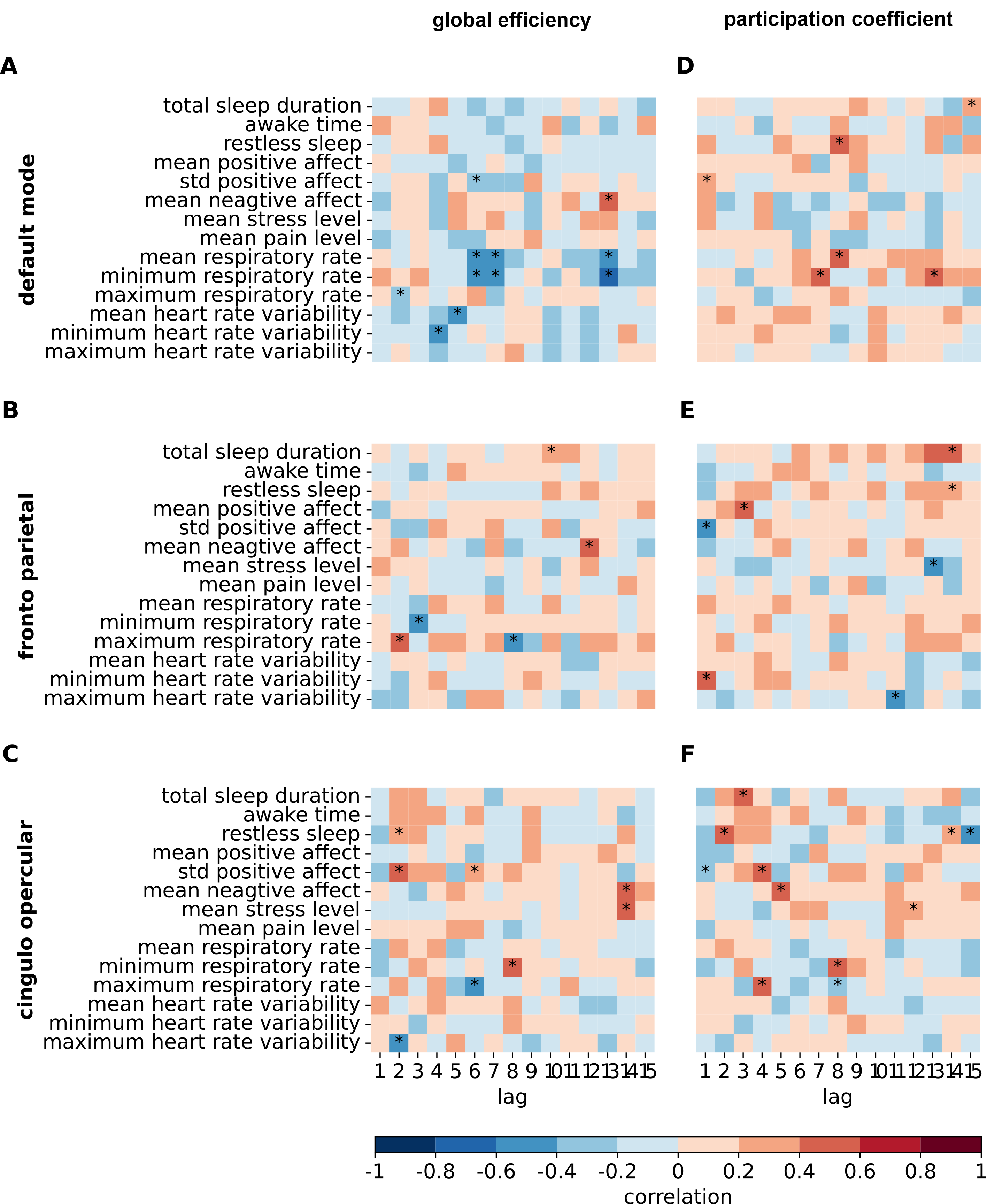

Supplement: S35 Fig — These results are derived by thresholding the network at 30% proportional threshold. Patterns from the 2 to 14 days in the past are correlated with the global efficiency in the (A) DMN, (B) FPN, and (C) CON. Similarly, sleep, activity and ANS activity patterns from the previous day up to 15 days in the past are correlated with the participation coefficient in the (D) DMN, (E)FPN, and (F) CON. Significant correlations are shown by an asterisk (*). Unprocessed study data can be found in the Zenodo data set release [175]. Processed results derived from the study data are accessible in the GIT repository [176], under the results folder. (TIF) [file pbio.3002797.s036.tif]

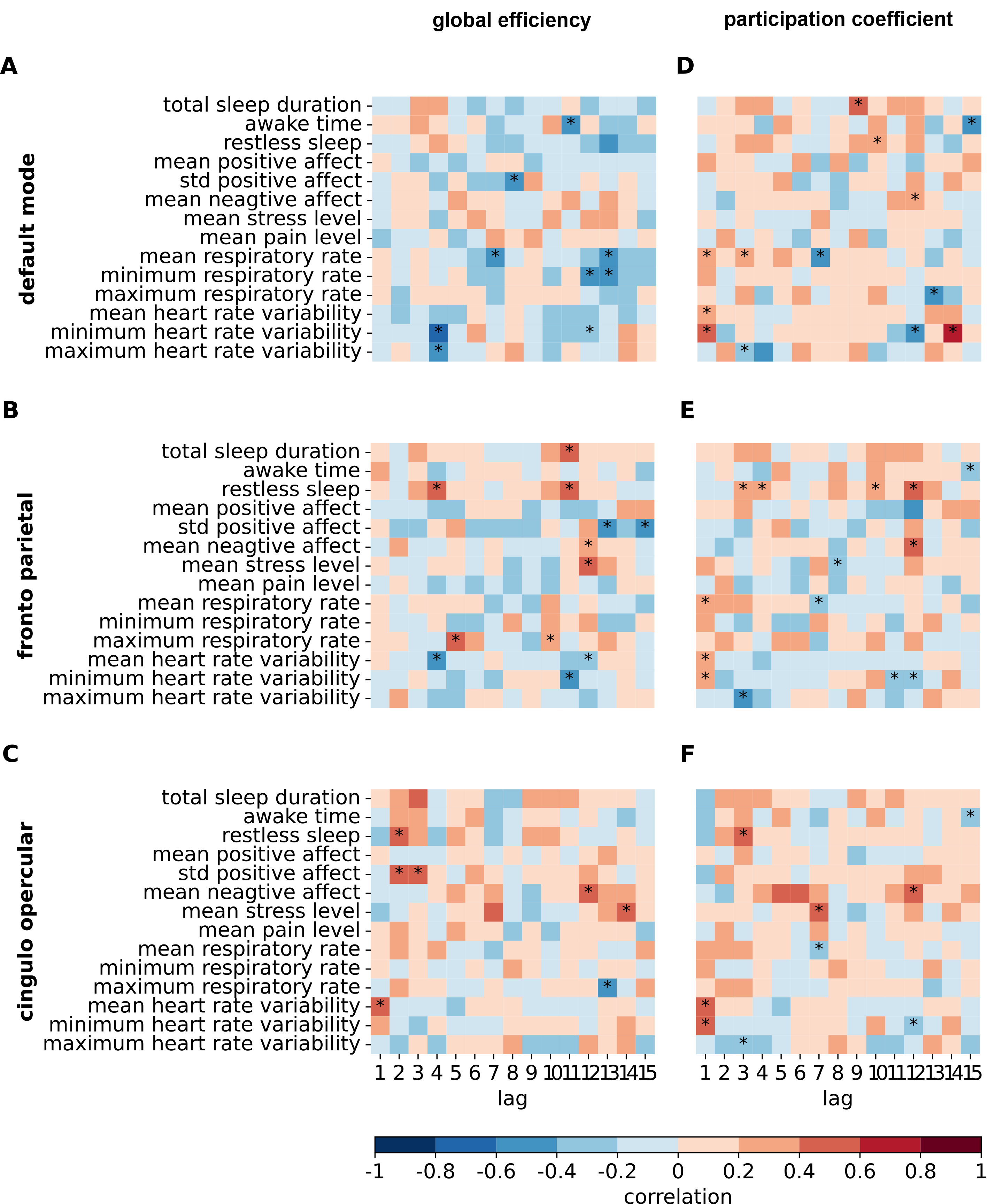

Supplement: S36 Fig — These results are derived by using the set2 from Seitzman and colleagues [158] with 10% proportional threshold. Patterns from the previous day to 15 days in the past are correlated with the global efficiency in the (A) DMN, (B) FPN, and (C) CON. Similarly, sleep, activity and ANS activity patterns from the previous day up to 15 days in the past are correlated with the participation coefficient in the (D) DMN, (E)FPN, and (F) CON. Significant correlations are shown by an asterisk (*). Unprocessed study data can be found in the Zenodo data set release [175]. Processed results derived from the study data are accessible in the GIT repository [176], under the results folder. (TIF) [file pbio.3002797.s037.tif]

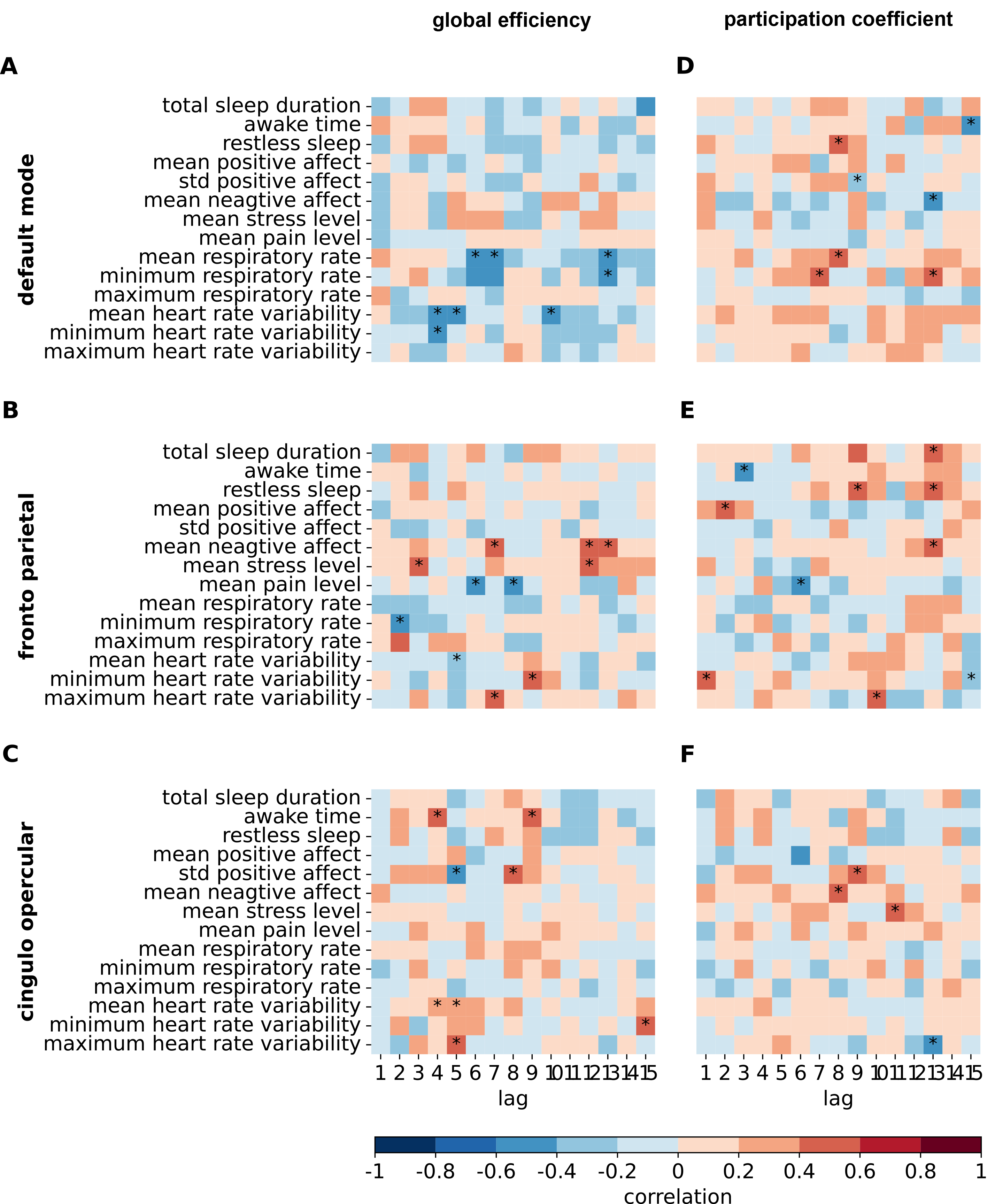

Supplement: S37 Fig — These results are derived by including the global signal as a regressor and using the set1 from Seitzman and colleagues [158] with 10% proportional threshold. Patterns from the 2 to 15 days in the past are correlated with the global efficiency in the (A) DMN, (B) FPN, and (C) CON. Similarly, sleep, activity and ANS activity patterns from the previous day up to 15 days in the past are correlated with the participation coefficient in the (D) DMN, (E)FPN, and (F) CON. Significant correlations are shown by an asterisk (*). Unprocessed study data can be found in the Zenodo data set release [175]. Processed results derived from the study data are accessible in the GIT repository [176], under the results folder. (TIF) [file pbio.3002797.s038.tif]

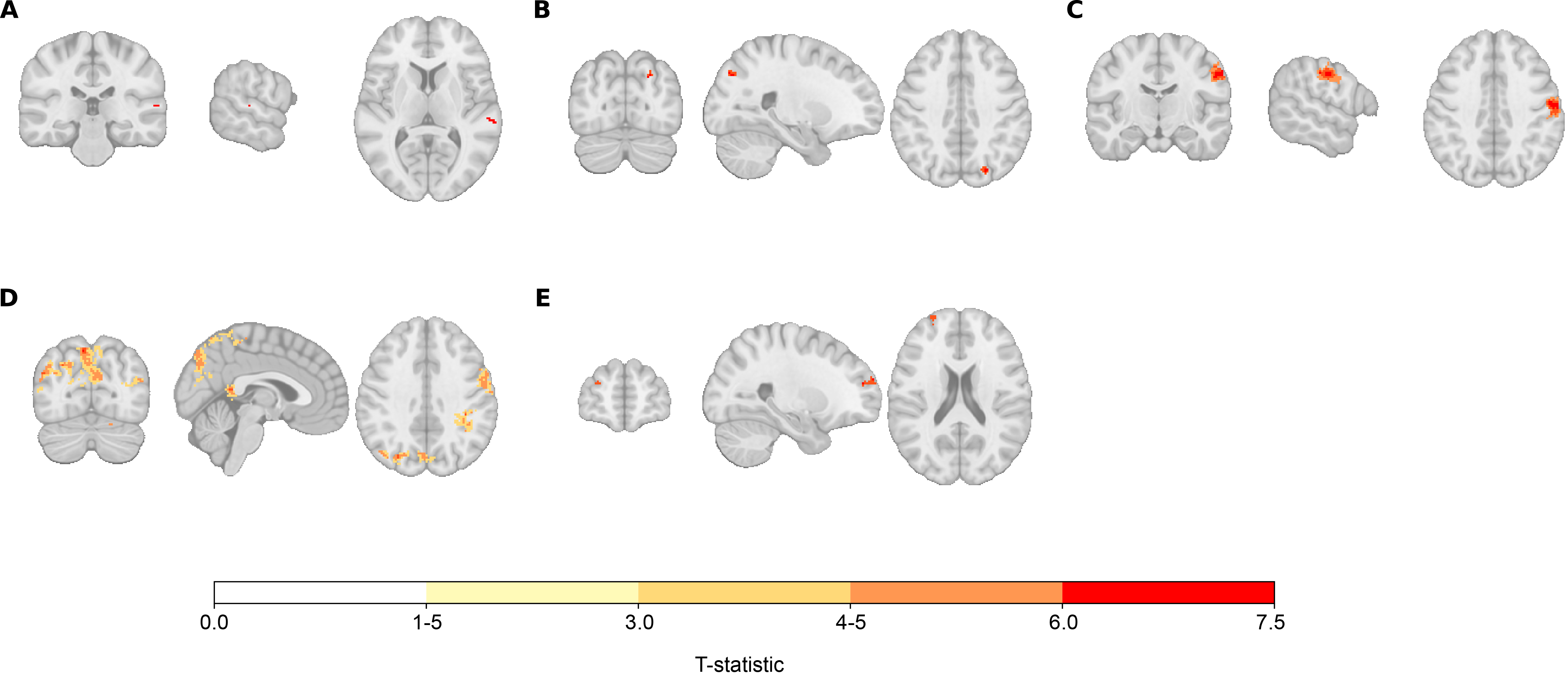

Supplement: S38 Fig — These results are derived by shifting the sliding window with 2TRs. The classification accuracy is related to: (A) standard deviation of positive affect at lag 2, (B) mean positive affect at lag 4, (C) maximum respiration rate at lag 8, (D) maximum respiratory rate at lag 11, and (E) maximum respiratory rate at lag 12. Brain plots were generated with nilearn [170]. Unprocessed study data can be found in the Zenodo data set release [175]. Processed results derived from the study data are accessible in the GIT repository [176], under the results folder. (TIF) [file pbio.3002797.s039.tif]

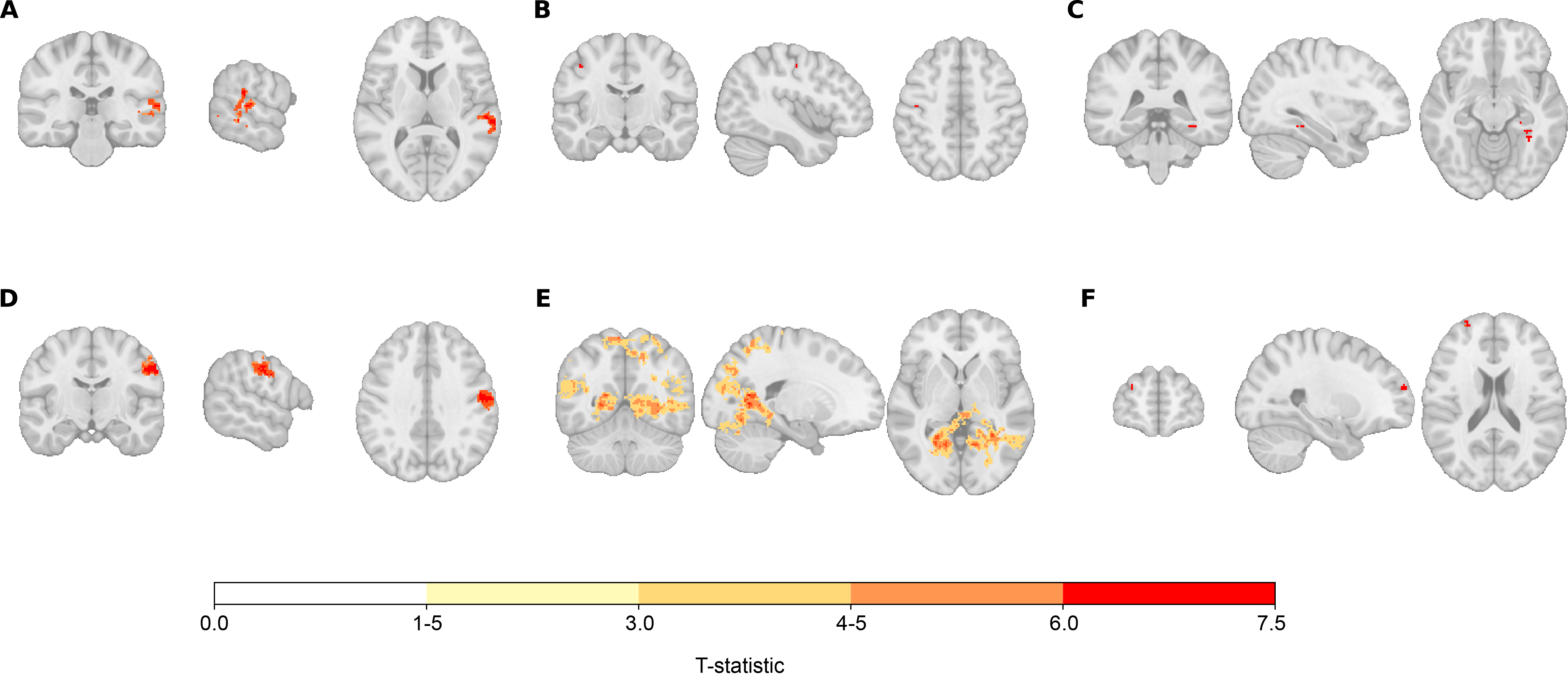

Supplement: S39 Fig — These results are derived by shifting the sliding window with 4TRs. The classification accuracy is related to: (A) standard deviation of positive affect at lag 2. (B) Total sleep duration at lag 2. (C) Mean positive affect at lag 4. (D) Maximum respiration rate at lag 8. (E) Maximum respiratory rate at lag 11. (E) Maximum respiratory rate at lag 12. Brain plots were generated with nilearn [170]. Unprocessed study data can be found in the Zenodo data set release [175]. Processed results derived from the study data are accessible in the GIT repository [176], under the results folder. (TIF) [file pbio.3002797.s040.tif]

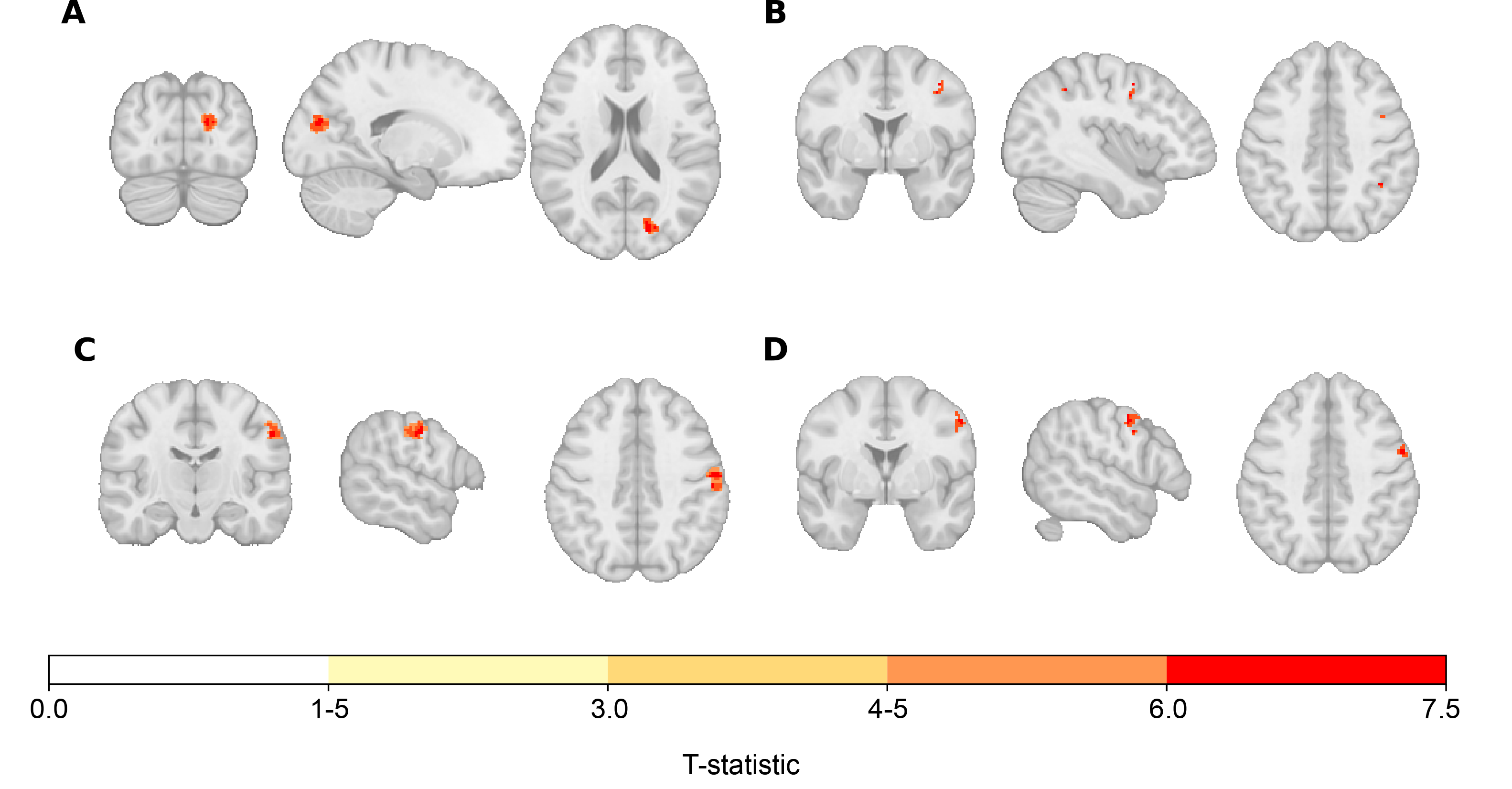

Supplement: S40 Fig — These results are derived by including the global signal as a regressor. The classification accuracy is related to: (A) mean pain lag 3. (B) Mean positive affect lag 4. (C) Maximum respiration rate lag 8. (D) Maximum respiratory rate lag 11. Brain plots were generated with nilearn [170]. Unprocessed study data can be found in the Zenodo data set release [175]. Processed results derived from the study data are accessible in the GIT repository [176], under the results folder. (TIF) [file pbio.3002797.s041.tif]

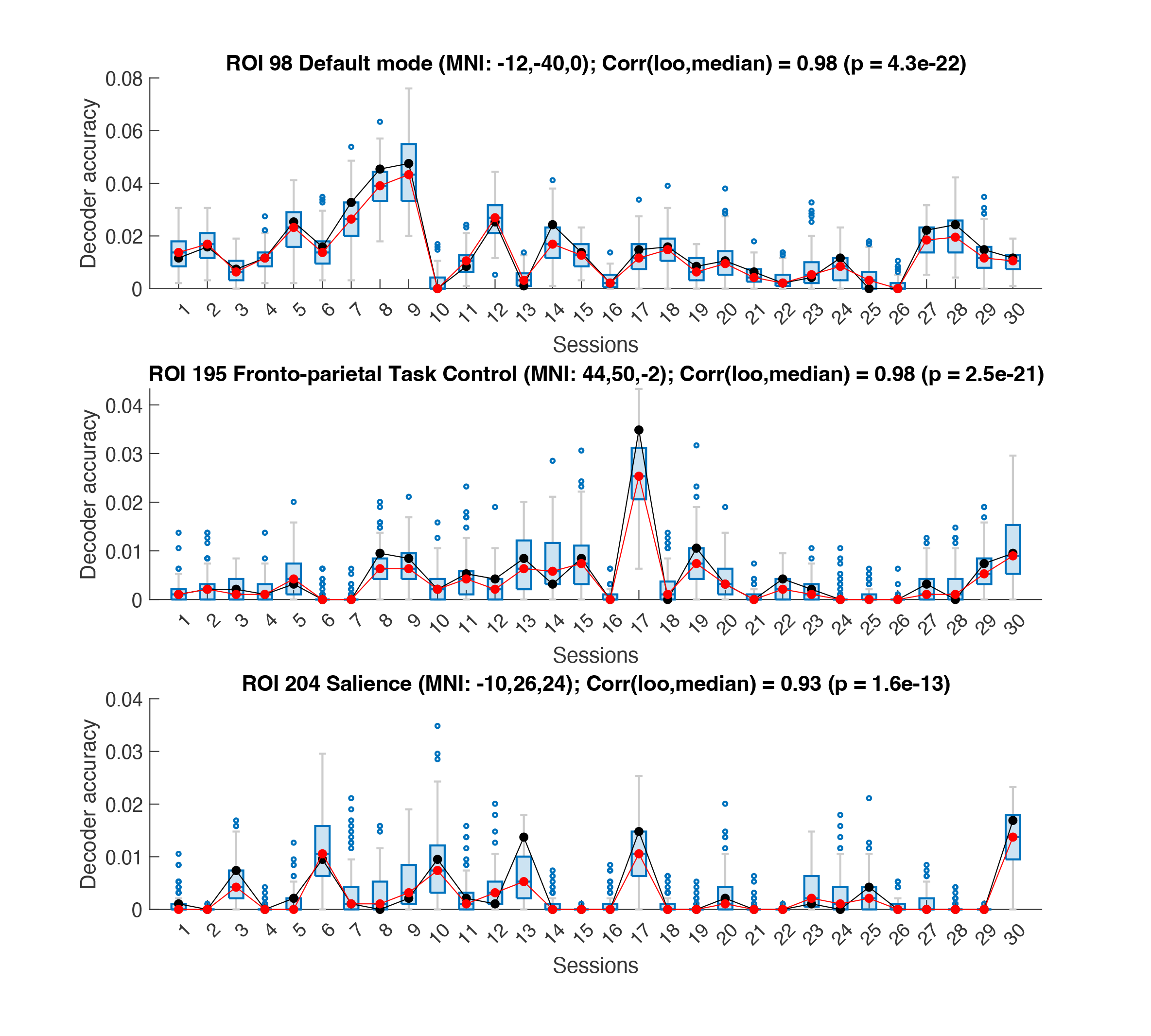

Supplement: S41 Fig — For 3 different regions of interest the correlations between the LOO and the median of the 20%-out are reported. Processed results derived from the study data are accessible in the GIT repository [176], under the results folder. (TIF) [file pbio.3002797.s042.tif]

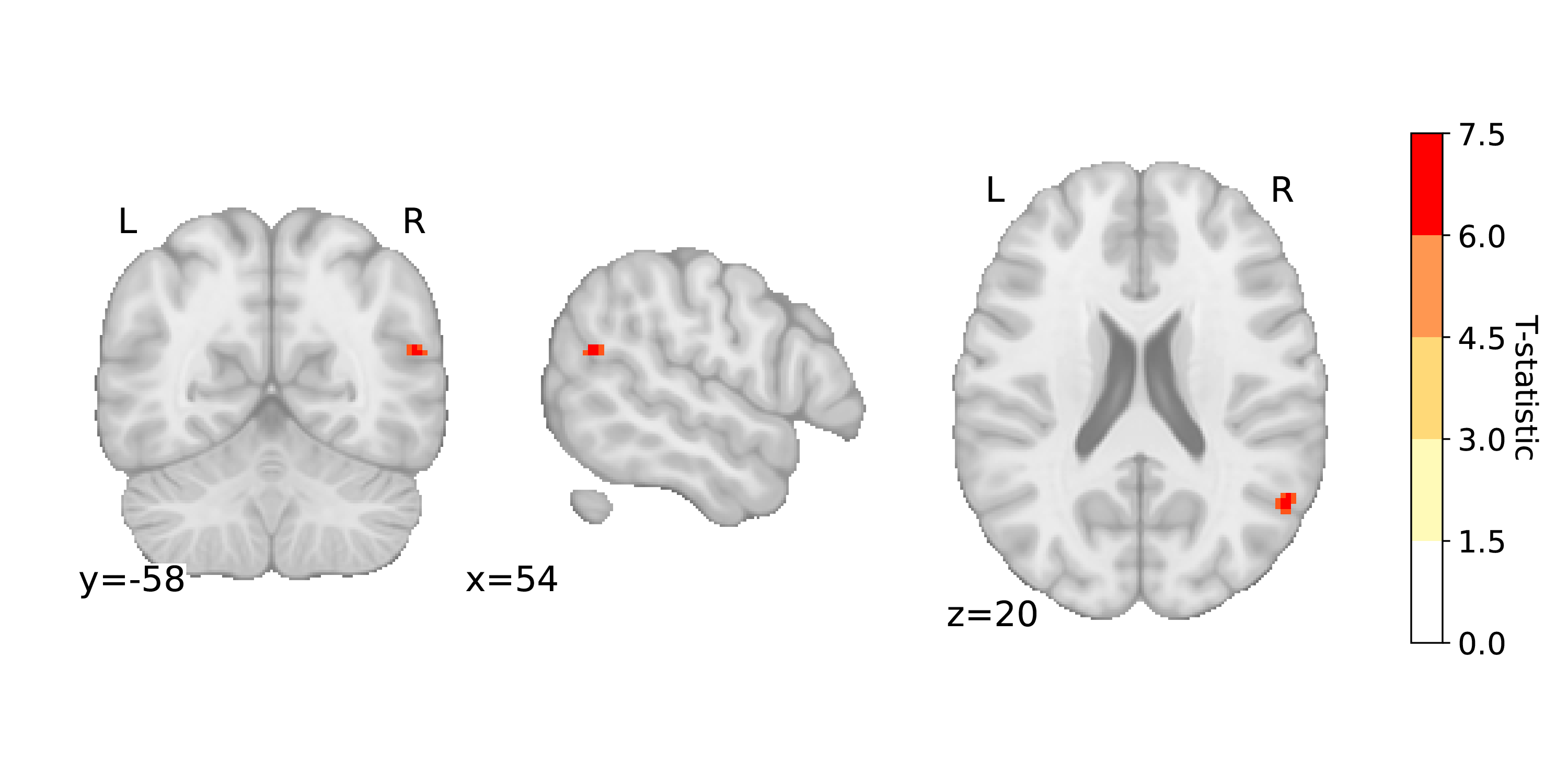

Supplement: S42 Fig — The cluster is located at MNI coordinates x = 55.04, y = −58.54, z = 19.89. The global signal was included as a regressor in the fMRI data preprocessing. Brain plots were generated with nilearn [170]. Unprocessed study data can be found in the Zenodo data set release [175]. Processed results derived from the study data are accessible in the GIT repository [176], under the results folder. (TIF) [file pbio.3002797.s043.tif]
