# Supplementary material for: Longitudinal single-subject neuroimaging study reveals effects of daily environmental, physiological, and lifestyle factors on functional brain connectivity
Source: PLoS Biol. 2024 Oct 8;22(10):e3002797. doi: 10.1371/journal.pbio.3002797 (PMC11460715; doi:10.1371/journal.pbio.3002797)
Supplement: Attachment — Submitted filename: PLOSBiology_reply2editor.pdf [file pbio.3002797.s075.pdf]

Ana María Triana, Juha Salmi, Nick Hayward, Jari Saramäki, Enrico Glerean

Effects of daily environmental, physiological, and lifestyle factors in functional brain connectivity

To the editorial staff and academic editors of PLOS Biology,

In this letter we reply to the concerns raised by the editors, we quote the original email from the editors in italic.

> *Your manuscript has now been evaluated by the PLOS Biology editorial staff, as well as by an academic editor with relevant expertise and an academic editor focused on specifically evaluating Preregistered Research Articles. I am writing to let you know that we would like to send your submission out for external peer review.*

We thank the editorial staff and academic editors for the positive feedback and for considering our submission for external peer review.

> *Prior to full submission, we ask that you address some of the conceptual framing concerns that have been raised by the Academic Editors. Specifically, both felt that the current proposal has the potential to provide useful information about daily behavioral factors that covary with individual brain variability. However, they expressed concern that the specific hypotheses laid out do not currently take full advantage of the individualized project design, tending to overlap with ideas already in the literature based on group studies, and that the broad nature of the hypotheses makes it difficult to understand how specific outcomes will help to confirm/refute these ideas.*

This is an important concern. While the current hypotheses build on existing literature of cross-sectional studies with the added novelty of within-individual longitudinal design, we propose to also pre-register an exploratory analysis which is not based on previous findings. For this analysis we cannot a-priori predict the significance of the relationships between brain activity and external behavioral sensor data, however we think that this exploration would add further novelty to the literature and it would take full advantage of the individualized design and also of the wide range of behavioral signals that we are able to collect in this study.

> *As it stands, there was concern expressed that any activity within these large-scale networks could be viewed a supporting your hypotheses. We therefore ask that you spend time developing a more precise breakdown of predicted activity under specific conditions and outcomes and how this will confirm or disconfirm each hypothesis.*

We have now defined more clearly the outcomes and interpretations to them from each hypothesis to better confirm or reject whether the brain-behavior associations known from cross-sectional studies reflect similar mechanisms that are consistent within an individual, or if previous results from group studies cannot be generalized to individuals.

> *Please also move the power analysis into the main text, expanding upon this analysis while also making it clear that this is an N=1.*

We have moved the power analysis to section Sampling plan.

Here a full list of changes from the previous version of the manuscript. The changes will be listed in the following format:

Page X, Line NNN:

Previous text:

> *This is the original text and in bold what has changed*

Current text:

> *This is the **changed** text and in bold and italic what has changed*

1. Page 3, line 106

Previous text:

> *To undertake the two challenges discussed above, we propose collecting an FPM dataset from one individual. This dataset will contain both brain activity data under a set of different fMRI tasks and objective data from external factors collected through automatic sensors. A carefully selected set of experimental conditions, including the Psychomotor Vigilance Test (PVT)41, an adaptive n-back task42, resting-state, and movie watching43 will allow us to understand the daily within-subject variability in attention, working memory, resting, and naturalistic stimuli tasks. At the same time, state-of-the-art smartphones and wearable devices make it feasible to measure (with low effort) external factors like sleep, physical activity, autonomic nervous system (ANS) activity, and mood34. These data will allow us to understand in real-life settings Q1: How is functional brain activity influenced by daily behavioral, physiological, and lifestyle factors (Table 1)? The relationship between the above factors and brain activity has been investigated cross-sectionally using a wide variety of paradigms. Although the setting differs from FPM, these studies still provide valuable hints about which brain areas and external factors are generally associated at the population level, and are thus worth investigating at the individual level as well. For example, several authors have reviewed in depth the relationship between brain activity and sleep44,45,46, physical activity47,48, autonomic nervous system (ANS) activity49,50 – comprising respiration rate, heart rate, and heart rate variability – and emotions and mood51,52. Based on these reviews and the studies cited therein, we approach Q1 through four specific hypotheses (see Table 1).*

Current text:

> *To undertake the two challenges discussed above, we propose collecting an FPM dataset from one individual. This dataset will contain both brain activity data under a set of different fMRI tasks and objective data from external factors collected through automatic sensors. A carefully selected set of experimental conditions, including the Psychomotor Vigilance Test (PVT)[41], an adaptive n-back task[42], resting-state, and movie watching[43] will allow us to understand the daily within-subject variability in attention, working memory, resting, and naturalistic stimuli tasks. At the same time, state-of-the-art smartphones and wearable devices make it feasible to measure (with low effort) external factors like sleep, physical activity, autonomic nervous system (ANS) activity, and mood[34]. These data will allow us to understand in real-life settings Q1: How is functional brain activity influenced by daily behavioral, physiological, and lifestyle factors (Table 1)? The relationship between the above factors and brain activity has been investigated cross-sectionally using a wide variety of paradigms. Although the setting differs from FPM, these studies still provide valuable*

hints about which brain areas and external factors are generally associated at the population level, and are thus worth investigating at the individual level as well. For example, several authors have reviewed in depth the relationship between brain activity and sleep[44,45,46], physical activity[47,48], autonomic nervous system (ANS) activity[49,50] – comprising respiration rate, heart rate, and heart rate variability – and emotions and mood[51,52]. Based on these reviews and the studies cited therein, we approach Q1 through ***five*** *specific hypotheses (see Table 1).*

2. Page 4, line 135

Previous text:

H3: Fluctuations of sleep and autonomic nervous system activity are correlated with brain connectivity in the default mode, frontoparietal, frontostriatal, thalamic, and motor networks during resting-state fMRI.

Current text:

H3: Fluctuations of sleep and autonomic nervous system activity are correlated with brain connectivity in the default mode, frontoparietal, thalamic, and **subcortical** *networks during resting-state fMRI.*

3. Page 5, line 169. We added the following text:

H5: Between-days time-segment classification accuracy is explained by daily behavioral, physiological, and lifestyle factors. While the prespecification of the hypotheses and analyses makes this registered report ideally suited for confirming and extending previous research results, the richness of the data collected in this study allows for further exploratory analyses[91]. In machine learning approaches in neuroimaging, a classifier is often trained on data from N-1 individuals and its accuracy is then tested with the subject that is left out and the average classification accuracy is reported. However, any observed inter-individual differences in classification accuracy in cross-sectional studies might depend on multiple confounding factors which are difficult to disentangle. For example, one cannot tell if the low classification accuracy for a subject is due to the brain anatomy and function per se or whether it is confounded with subject's state at the time of the measurement related to behavior. These include whether the subject slept well the night before the scanning session and the subject's phenotype (height, weight, etc). Moreover, phenotypic factors have also been linked to in-scanner confounding variables such as spatio-temporal patterns of head motion[92], making the challenge even more difficult. Previous studies have tested the accuracy of a classifier which identified consistent spatio-temporal patterns for movie segments of 15 s using hyperalignment[93] to minimize inter-individual differences and maximize classification accuracy[43]. Findings show highest classification accuracy in primary sensory areas as well as in areas related to the theory of mind, important during movie watching with social cues.

With multiple scans of a single subject, we can control for phenotypical, anatomical, and functional brain differences, so that the classification accuracy for each scanning session can then be directly related to daily fluctuations in behavioral,

physiological, and lifestyle factors. As this type of analysis has not been conducted in prior studies, we will keep this as an exploratory analysis. We will conduct similar time-segment classification with the leave-one-out approach similar than Visconti di Oleggio Castello and colleagues[43], using fmri movie data from N-1 days to train the classifier and testing it on the day that is left out for each of the days (runs) in the dataset. The classification accuracy for each daily scanning session will then be compared to other measured external factors using regression models, similarly to the other presented hypotheses H1-H4.

4. Page 5, line 211:

Previous text:

All data collected will be pseudonymized and released with this registered report. We hope this dataset will be useful to other researchers for investigating within-subject variability in brain function during tasks, alongside natural physiological variations, and for developing new methodologies for the analysis of multivariate brain and sensor data. This dataset will allow us to understand how task-evoked brain activity is influenced by daily physiological, behavioral, and lifestyle factors, advancing the understanding of brain-behavior dynamics. It will also allow us to use less artificial stimuli and take into account more natural situations over different time scales, which are a current challenge in current human behavior research92. This experiment can reveal clues about how results at the population-level are related to results at the individual level and can support the design of future precision-mapping studies with clinical populations for personalized medicine.

Current text:

All data collected will be pseudonymized and released with this registered report. ***This experiment presents a reductionist approach as reliability and robustness are embedded in the design. By using data from one subject, we remove many sources of confounds that are common in cross-sectional designs; for example, controlling for anatomical and functional factors, weight, movement, etc.*** *We hope this dataset will be useful to other researchers for investigating within-subject variability in brain function during tasks, alongside natural physiological variations, and for developing new methodologies for the analysis of multivariate brain and sensor data. This dataset will allow us to understand how task-evoked brain activity is influenced by daily physiological, behavioral, and lifestyle factors, advancing the understanding of brain-behavior dynamics. It will also allow us to use less artificial stimuli and take into account more natural situations over different time scales, which are a current challenge in current human behavior research[94].* ***This setting is not only important to understand how population-level findings are related to results at the individual level, but also the conditions for the phenomena to be reproducible. We hope that the results from this experiment*** *can support the design of future precision-mapping studies with clinical populations for personalized medicine.*

5. Page 12, line 454: the text is modified. We also added Figure 2.

Previous text:

Sample size

We will collect daily behavioral data for nineteen weeks. Simultaneously, we will also collect thirty MRI scans over fifteen weeks. We have opted for thirty scans as a compromise between the experiment load to the subject and sufficient number of scans for ISC analysis[108]. In addition, power analysis of pilot data for PVT and n-back tasks revealed that a minimum of fourteen samples are required to detect voxel activations given the presented stimuli. These fourteen samples yielded 95% power, once values are corrected with random field theory. Consequently, thirty scans should suffice. Power analysis for PVT and n-back tasks were done using second-level general linear models (GLM) from the pilot data (see Supplementary information, Pilot III) and computed by neuropower[109]. Results from these analyses are openly available (see Code availability). Finally, the proposed sample size has been previously used in similar PM studies[4,28] where fluctuations of brain connectivity across time were investigated.

Current text:

Sample size

*We will collect daily behavioral data for nineteen weeks **from one individual (N=1).** Simultaneously, we will also collect thirty MRI scans over fifteen weeks **from the same individual.** We have opted for thirty scans as a compromise between the experiment load to the subject and sufficient number of scans for all tasks according to the power analysis.*

Power analysis

*Power analysis of pilot data for PVT and n-back tasks revealed that a minimum of fourteen samples are required to detect voxel activations given the presented stimuli. These fourteen samples yielded 95% power, once values are corrected with random field theory. Power analysis for PVT and n-back tasks were done using second-level general linear models (GLM) from the pilot data (see Supplementary information, Pilot III) and computed by neuropower[110]. Results from these analyses **are shown in Fig. 2 and** are openly available (see Code availability).*

As assessing power calculations for model-free stimuli (i.e. resting-state and ISC) is more difficult, we based our calculations on proposed sample sizes that have been previously employed. For resting-state fMRI, 30 scans have been used in similar PM studies where fluctuations of brain connectivity across time were investigated[4,28]. For naturalistic stimuli, previous work[111] has established that 30 scans are within the optimal number of samples for the ISC statistics to converge.

Figure 2. Power analysis por (A) PVT and (B) n-back tasks computed by neuropower[108]. Power curves are shown for uncorrected values (green), random field theory (orange), and Bonferroni (blue) corrected values. Fourteen scans are necessary to achieve 95% power using Random Field Theory for both tasks.The curves also show that samples bigger than twenty scans assure enough power for all types of corrections in both tasks.

6. Page 18, line 666. We added the following text:

Between-days movie time-segment classification

Following the work of Visconti di Oleggio Catello and colleagues[43], we will use the same leave-one-out classifier on our naturalistic movie-watching data. The approach is described in detail in the zenodo release[141]. Briefly, the movie fMRI time-series are divided into overlapping segments of 14.85 seconds (25 TRs, in the original paper segments were 15 seconds long). The segments are obtained with a sliding window of 25 TRs, with sliding time equal to 1 TR, i.e. each segment has 24 time-points overlapping with the previous segment for a total of 946 segments. Then, the classifier is implemented by calculating the pairwise similarity (correlation-based distance) between the n-th segment from the left-out movie fMRI session and the average signal from the n-th segment from the other sessions. The pairwise similarity is computed over a searchlight sphere of 10mm radius and the segment with the highest similarity is chosen as the predicted one (chance level equals 1/946). The accuracy of the classifier is then estimated as the percentage of correctly classified segments across the whole run that is left out. The procedure is repeated for all searchlight spheres covering the whole gray matter brain areas. For each day, a brain map with values of classification accuracy will be obtained. These maps can then be correlated with external factors as described in the previous sections using linear regression and correction for multiple comparisons with FSL randomise over 5000 permutations.

The proposed classification analysis in this manuscript differs from the original one in the following: Firstly, in the original study the TR was 1 second, i.e. the temporal distance between consecutive time segments is 1 second which is almost twice as big than the distance in this study (TR = 0.594). Because of this, we will explore a few temporal segmentation approaches by shifting the sliding window with 1TR, 2TRs, 4TRs. Secondly, contrary to the original study, we will not perform hyperalignment as the subject is the same across all sessions. Thirdly, in the original study only the cortical surface was considered after transforming the volumetric data with freesurfer. Here, we will keep the data in volumetric format as it has two advantages, (i) we can also explore subcortical areas, and (ii) we can better control for false positives using the threshold-free cluster enhancement correction as implemented in FSL randomize since the classification accuracy for each day is correlated with other behavioral and physiological time series obtained outside the scanner. Finally, although still widely used in the neuroimaging literature, leave-one-out approaches have limitations due to potential overfitting and outliers effect[142]. For this reason, we will also explore other forms of cross-validation with repeated random splits by leaving out 20% of the sessions.

7. Page 22, Summary table, H1, Interpretation given to different outcomes column:

Previous text:

A significant interaction will be interpreted as evidence of the effects of sleep on functional brain connectivity under attentional tasks for the specific functional brain networks that yielded statistical significance. The coefficient of determination will be interpreted as the strength of the relationship between the external factor and the functional connectivity. Absence of statistical significance will be interpreted as failure to reject the null hypothesis

Current text:

> ***A statistically significant interaction will be interpreted as evidence that a decrease in sleep time, efficiency, and latency is related to decreased link weight in the fronto-parietal, sensory, and thalamic networks and increased link weight in the default mode network. Similarly, a decrease in sleep time, efficiency, and latency is related to decreased global functional network efficiency.*** *The coefficient of determination will be interpreted as the strength of the relationship between the external factor and the functional connectivity. Absence of statistical significance will be interpreted as failure to reject the null hypothesis.*

8. Page 22, Summary table, H2, Interpretation given to different outcomes column:

Previous text:

> *A significant interaction will be interpreted as evidence of the effects of sleep or activity on functional brain connectivity under working memory tasks for the specific functional brain networks that yielded statistical significance. The coefficient of determination will be interpreted as the strength of the relationship between the external factor and the functional connectivity. Absence of statistical significance will be interpreted as failure to reject the null hypothesis.*

Current text:

> ***A statistically significant interaction will be interpreted as evidence that a decrease in sleep time, efficiency, sleep latency, and steps is related to decreased link weight in the fronto-parietal, anterior cingulate cortex, and motor areas and increased link weight in the default mode network. Similarly, an increase in the inactive time is related to decreased global efficiency.*** *The coefficient of determination will be interpreted as the strength of the relationship between the external factor and the functional connectivity. Absence of statistical significance will be interpreted as failure to reject the null hypothesis.*

9. Page 23, Summary table, H3, hypothesis column:

Previous text:

> *H3: Fluctuations of sleep and ANS activity are correlated with brain connectivity in the default mode, frontoparietal, frontostriatal, thalamic, and motor networks during resting-state fMRI.*

Current text:

> *H3: Fluctuations of sleep and ANS activity are correlated with brain connectivity in the default mode, frontoparietal, thalamic, and* ***subcortical*** *networks during resting-state fMRI.*

10. Page 23, Summary table, H3, Interpretation given to different outcomes column:

Previous text:

A significant interaction will be interpreted as evidence of the effects of sleep on functional brain connectivity under resting-state for the specific links and measures that yielded statistical significance. The coefficient of determination will be interpreted as the strength of the relationship between the external factor and the functional connectivity. Absence of statistical significance will be interpreted as failure to reject the null hypothesis.

Current text:

A statistically significant interaction will be interpreted as evidence that a decrease in sleep time, efficiency, and latency is related to decreased within-network connectivity in the DMN. Similarly, an decrease in the heart rate variability and sleep time is related to a decrease in the link weight between thalamic, subcortical, frontoparietal, and default mode networks. *The coefficient of determination will be interpreted as the strength of the relationship between the external factor and the functional connectivity. Absence of statistical significance will be interpreted as failure to reject the null hypothesis.*

11. Page 23, Summary table, H4, Interpretation given to different outcomes column:

Previous text:

A statistically significant effect in an ROI will be interpreted as evidence that similarity of the daily brain activity is related to the similarity of sleep or mood. Absence of statistical significance will be interpreted as failure of proof for the alternative hypothesis.

Current text:

A statistically significant effect will be interpreted as a positive relationship between the similarity of an ROI and the similarity of the behavioral score. For example, the more similar the signal in the precuneus between two days, the more similar the behavioral scores for those two same days, if the statistical significance is above the FDR corrected p-value. *Absence of statistical significance will be interpreted as failure of proof for the alternative hypothesis.*

12. Page 24, Summary table, H5, we added the following text to the hypothesis column:

H5: Between-days time-segment classification accuracy is explained by daily behavioral, physiological, and lifestyle factors.

13. Page 24, Summary table, H5, we added the following text to the sampling plan column:

30 samples (Pajula & Tohka, 2016)[108]

14. Page 24, Summary table, H5, we added the following text to the analysis plan column:

We will conduct a regression analysis of the total sleep time, sleep efficiency, steps, and heart rate variability on the classification accuracy brain maps. The

classification brain maps will be obtained following Visconti di Oleggio Castello and colleagues43. For each 10mm-sphere, we will divide the data in 25TR segments with a slide window of 1TR. Then, we will train a classifier calculating the pairwise similarity between the left-out segment of the left-out run and the average signal from the segments of the other runs. Statistical validity will be assessed via FSL randomise over 5000 permutations. This hypothesis is exploratory, other classification approaches will also be tried (e.g. k-fold cross validation instead of leave-one out).

15. Page 24, Summary table, H5, we added the following text to the interpretation of outcomes column:

> ***A statistically significant interaction will be interpreted as evidence that a decrease in sleep time, sleep efficiency, steps, and heart rate variability are related to decreased classification accuracy. The coefficient of determination will be interpreted as the strength of the relationship between the external factor and the classification accuracy. Absence of statistical significance will be interpreted as failure to reject the null hypothesis.***
