# Supplementary material for: Longitudinal single-subject neuroimaging study reveals effects of daily environmental, physiological, and lifestyle factors on functional brain connectivity
Source: PLoS Biol. 2024 Oct 8;22(10):e3002797. doi: 10.1371/journal.pbio.3002797 (PMC11460715; doi:10.1371/journal.pbio.3002797)
Supplement: Attachment — Submitted filename: PlosBiology_ReplytoReviewers.pdf [file pbio.3002797.s076.pdf]

Ana María Triana, Juha Salmi, Nick Hayward, Jari Saramäki, Enrico Glerean

Effects of daily environmental, physiological, and lifestyle factors in functional brain connectivity

To the editorial staff and academic editors of PLOS Biology,

We would like to thank all reviewers and editors for their enthusiasm and very positive view of our manuscript — reviewer 1 finds that "*this an important direction for future research, and the dataset generated from this study is exciting",* reviewer 2 commends us for taking on the project, and reviewer 3 points out that "*as studies of this nature are rare, this work and the accompanying dataset are likely to generate valuable information about the links between brain connectivity measures and individual variability."*

We are also thankful for the reviewers' constructive feedback that has helped us to further improve the manuscript. In particular, we appreciate the comments regarding additional analyses that are made possible by the proposed experimental design, as they reinforce our belief that the experimental setup is unique and the resulting data is of high interest to the scientific community.

In the first version of the manuscript, we proposed four hypotheses based on existing literature. For each hypothesis, the behavioral variables were carefully chosen after a thorough literature review, in line with the purpose of the registered report format, where the focus is mainly on testing well-defined hypotheses founded on literature.

In the first revised version, in response to the editor's comments, we included an additional hypothesis based on exploratory analysis. In the current version, as requested by Reviewer 3, we have included another exploratory question to take advantage of our longitudinal design. This additional question explores three new hypotheses and also includes the aforementioned hypothesis. Each new hypothesis expands on the original four hypotheses to investigate the effects of behavioral factors on brain connectivity over different timescales. All hypotheses will be tested by a large number of analyses involving data coming from more than seven distinct sources.

As pointed out by the reviewers, the dataset to be collected will be rich, and there are several additional analyses that would be interesting and worth pursuing. However, taking into account the length and coherence of the manuscript, we hope that editors and reviewers agree that a few of these additional analyses will have to be left out from this work and rather reported separately after this first manuscript is finished. As we will provide open data, these exploratory analyses can be pursued either by us or other members of the scientific community. Changes are highlighted in the manuscript in blue font. Below, we quote the original email from the editors and reviewers in italic.

> *Thank you for your patience while your manuscript "Effects of daily environmental, physiological, and lifestyle factors in functional brain connectivity" was peer-reviewed at*

PLOS Biology. It has now been evaluated by the PLOS Biology editors, an Academic Editor with relevant expertise, and by three independent reviewers.

In light of the reviews, which you will find at the end of this email, we would like to invite you to revise the work to thoroughly address the reviewers' reports. As you will see below, the reviewers are broadly positive about your proposed study, they each have a number of requests and suggestions that will need addressing before further consideration.

Given the extent of revision needed, we cannot make a decision about publication until we have seen the revised manuscript and your response to the reviewers' comments. Your revised manuscript is likely to be sent for further evaluation by all or a subset of the reviewers.

Below, we address each referee's comments in detail.

Reviewer #1:

This manuscript presents a pre-registered report of a study with deep phenotyping, monitoring, and brain imaging in a single individual. This approach is an important direction for future research, and the dataset generated from this study is exciting. The collection of daily behavioral data via wearables and smartphone questionnaires along with frequent neuroimaging is novel and may provide a more reliable approach to measuring brain-behavior associations. I commend the authors for taking on this project.

We thank reviewer #1 for the positive feedback.

I have some comments and questions for the authors to address:

1. It is not clear why all aspects of hypotheses 3 and 4 are specific to resting state fMRI and movie watching, respectively. For example, why is mood part of H4, but not H3? Would you predict that mood would be associated with resting state vs. movie watching differently?

We have modified H3 and H4 to match the external factors that will be evaluated in relationship to the functional connectivity. H3 in (page 4, lines 137-139) now reads "Fluctuations of sleep, autonomic nervous system activity, and mood are correlated with brain connectivity in the default mode, frontoparietal, and subcortical networks during resting-state fMRI." Similarly, H4 in (page 4, lines 157-159) now reads "Increased similarity in sleep, autonomic nervous system activity, or mood between days is seen as an increase of inter-day similarity in the frontoparietal, default mode, and salience networks during movie-watching tasks." These changes are reflected in the text and in the summary table as well.

Regarding H4, there is evidence that movie fMRI can drive higher-order neural processes, enabling us to capture brain dynamics under complex, ecological conditions (Eickhoff et al., 2020). In this case, movie fMRI exhibits an advantage compared to rs-fMRI when studying emotions. In fact, there are several studies studies linking emotional components to movie fMRI

(see Jääskeläinen et al., 2021 for a review) and even classification of emotional patterns has been achieved when using movie fMRI (Kragel & LaBar, 2015; Saarimäki et al., 2016, Karama et al., 2011, and Saarimäki et al., 2022).

Regarding H3, ANS has been widely employed in rs-fMRI studies, most of the time to study the role of physiology as a confound for resting state studies (see Birn, 2012 and Murphy et al., 2013). However, it is uncommon to collect autonomic nervous system measures outside the scanner and analyze it along fMRI data, which is a point that we want to explore.

As the external factors evaluated in H3 and H4 are now the same, this will allow us to understand whether, e.g., mood is associated with resting state and movie watching conditions differently, and in general, we will be able to draw a qualitative comparison between the two tasks. This is a point we will include in the discussion part of the paper once the data has been analyzed.

In addition to the aforementioned changes, we included some new references and words in the text to justify the inclusion of ANS and mood in H3 and H4. These changes are:

page 4, line 144: we included the word mood.

page 4, line 164-165: we included the sentence “and physiological changes are correlated with different stimuli within the movies”.

> *2. Given how well this subject holds still in the scanner, it is not clear why more conservative methods for accounting for motion aren't planned. The proponents of strict motion methods in the field are cited in this paper (e.g., Power et al. 2014), yet the recommended criteria are not planned. For example, motion censoring is often recommended for volumes exceeding FD=0.2mm and global signal regression is touted as best accounting for motion (Power et al. 2014, Ciric et al. 2017, 2018, Satterthwaite et al. 2019). The authors state that they will not censor volumes from the movie watching scans because the proposed analyses require a consistent number of volumes for each day. A straightforward approach would be to censor volumes at a threshold of FD < 0.2mm, identify the scan day with the least number of volumes remaining, and then randomly censor additional volumes on the other days in order to match the numbers across days. Given how well this subject holds still, it is unlikely that this will yield too much data loss. But, it will ensure that the few high motion points do not contaminate the data. In any case, the authors should propose to test more strict processing methods in addition to the currently planned methods.*

This is an important point and we agree with the reviewer. We have modified the manuscript to reflect the use of volume censoring for both, rs-fMRI and the movie watching condition using the FD>0.2 criteria. Specifically for movie fMRI, the same time point can be censored across all days if for one day that time point had high motion (FD>0.2).

It is important to remember that this type of data collection is different from cross-sectional studies. In cross sectional studies, we would exclude those subjects that do not have enough good-quality data (FD<0.2). Since our study design is different, we would like to propose that if after scrubbing with FD<0.2, we lose more than 20% of time points, we will adopt a 0.5 threshold. This is of course unlikely as our participant is experienced and able to hold still quite well, as the reviewer pointed out. We have modified the manuscript in (page 15, line 547-554):

"rs-fMRI volumes will be excluded because of high movement (i.e., volumes whose FD>0.2). If this exclusion leads to losing more than 20% of the data points, we will use a more flexible threshold (FD<0.5). Scrubbed volumes could be different for each session of the rs-fMRI data. For movie-fMRI, we will detect the volumes with high movements (FD>0.2) in each session and censor them across the 30 sessions. Similar to resting state, if this exclusion leads to losing more than 20% of the data points, we will increase the threshold to FD>0.5. Discarding volumes does not affect the sampling size (i.e. the number of scans to be taken)"

> *Regarding global signal regression, I understand this is a contentious topic in the field. Therefore, I recommend that the authors propose to analyze the data both with and without global signal regression. The carpet plots in the supplement actually give some support for using global signal regression. They clearly show the beneficial effects of denoising, but there is still some banding where motion affected the signal across the whole brain. This is why the author of the paper cited describing the carpet plots (Power 2017) recommends global signal regression.*

We agree with the reviewer and we have edited the manuscript in (Supplementary File, Page 7, lines 1091-1094) "Since the global signal appears to be highly correlated with parietal areas, we decided not to regress it out in the primary analysis. However, because the global signal is a contentious topic in the field, we will also run the analysis with a denoising strategy that includes global signal regression".

> *3. The authors state that the adjacency matrices will be "subdivided into smaller network-specific matrices according to Power and colleagues138." There is a lot that goes into this analysis, so more detail is needed. For example, do the authors plan to use the Infomap algorithm? If so, what thresholds will you use? How do you propose to create consensus communities? There are more updated and improved methodological details for these approaches as well, such as that reported in Gordon et al. 2017, which is probably more appropriate for the proposed study. I recommend that the authors describe the details of these planned analyses.*

We apologize that the text about the method was not clear. As our goal is to study how previous cross-sectional findings are linked to single-participant level in a longitudinal design, we consider a widely used predefined parcellation as the most viable option. This also makes our main analyses more straightforward, making sure that there are no biases potentially affecting the analyses of brain connectivity over time or between different conditions. Another possible option would be to use approaches similar to Gordon and colleagues (2017) or Gratton and colleagues (2018). There could certainly be a possible benefit of improved precision at

individual level. However, we think that validating such a method for the purposes of our design would require further work and see that it might be a risky choice here. We expect that at network level, almost any robust finding will be observed with multiple different parcellation methods.

After a careful consideration, we performed a revision to the analysis plan regarding parcellation. Previously, we have planned to use the Brainnetome parcellation and map the networks from Power et al. (2011). However, this mapping was indeed confusing and we will leave that step out from the analysis, using the parcellation by Power et al. (2011) in a conventional manner.

This has been clarified in page 18, lines 669-670: “Functional network nodes will be defined based on the parcellation defined by Power and colleagues. This parcellation comprises 264 putative functional areas”

Because of this change in parcellation, some minor modifications in the hypotheses were made to match the network names provided by Power et al. (2011). These changes are:

page 3, line 111: we replaced the word “thalamic” for “subcortical”

page 4, line 127: we deleted the words “anterior cingulate cortex”

page 4, line 138: we deleted the word “thalamic” as these areas are included in the “subcortical” network.

> *Minor:*
>
> *1. I recommend using the term "Precision functional mapping (PFM)" instead of FPM to be consistent with the literature (e.g., Gordon et al. 2017).*

We changed the term functional precision mapping to precision functional mapping across the whole manuscript.

> *2. Pg. 3, line 109: what is meant by "exacerbates the performance in attentional task"?*

The line was rephrased to (page 3, line 113-114) “the amount of accumulated awake time is associated with the performance in attentional tasks” to clarify.

> *3. There is likely a typo on pg. 3, line 117 "sustained ascending arousal from the thalamus."*

The word “input” was missing. This part of the sentence (page 4, lines 122-123) now reads “sustained ascending arousal input from the thalamus supports a reciprocal inhibition between the fronto-parietal network (FPN) and default mode network (DMN)”

> *4. What is the reason for using two versions of the PVT (10 min in the scanner and 5 min out of the scanner)?*

There are multiple validated versions of the PVT with different lengths that can be used for different purposes. With regards to measurement of behavioral effects (e.g., the effects of sleep on vigilance), 3 to 5 minutes is usually considered to be sufficient (Basner et al., 2011), with the 5-min PVT being a reliable substitute for the 10-min PVT (Thompson et al., 2022). In the case of fMRI, a sufficient amount of brain volumes are required, and typically longer versions are used. 10-minutes is the standard PVT that has been widely used both in behavioral (Basner et al., 2011) and in brain imaging studies. Moreover, the subject's feedback on length of the study and effort are also taken into account, agreeing on a shorter task for the daily data collection. A shorter daily task will lower the risk of missing data, while retaining the reliability of the test.

We added two sentences explaining this rationale (page 9, lines 349-351) "In fact, Basner and colleagues have demonstrated that three to five minutes is usually sufficient to measure effects of sleep on vigilance." and (page 9, lines 353-356) "We use a longer PVT task in the scanner because usually in fMRI, longer versions are preferred to collect more volumes. Nevertheless, using a 5-min and a 10-min PVT task should not pose any problems given the 5-min PVT is a reliable substitute for the 10-min PVT".

> 5. *"MRI will be performed in a fixed schedule, Mondays and Fridays at 1400 hours, subject to the scanner availability." How likely are the experimenters to follow this schedule given the typical availability of the scanner?*

We will conduct our MRI measurements at the Advanced Magnetic Imaging Center, which maintains a research-dedicated 3T MRI scanner. It is possible to reserve the scanner weeks ahead and there is advance information on any possible maintenance or unavailability of the device. Therefore, because of predictable scanner availability, the likelihood of being able to follow the schedule is far greater compared to experiments that use hospital scanners. However, it is still possible that some unforeseen event prevents us from strictly following the schedule (e.g., ill subjects are not allowed to enter premises, or there is a sudden need for critical maintenance). In that case, we will reschedule the scanning time to the next available date and extend the data collection accordingly. Neither of these events will compromise the amount of data points, i.e., thirty scans will be collected in any case.

Due to scanner availability, we have changed the scanning time from 1400 to 1130, as the scanner was no longer available on all Fridays at 1400. This change is reflected in (page 12, line 436) "MRI will be performed in a fixed schedule, Mondays and Fridays at 1130 hours". We also anticipate that scanning during weeks 51 and 52 may be difficult because of Christmas holidays. Nevertheless we will not pause the behavioral data collection during the holiday period, and if necessary, we will extend the protocol for two more weeks.

It is good to note that all analysis can still be performed even if the planned schedule cannot be strictly followed. In this case, we will create a regressor for the time and day of the scan and use it. We have clarified this by adding the following sentence (page 15, lines 561-565) "In case of missingness in the MRI data, we will reschedule the session, schedule permitting; the length of the overall data collection will be extended accordingly. If more than 20% of the sessions need to be rescheduled to a different hour or day of the week, time and day of the scanning session will be taken into account in a regressor".

6. What is the reason for having a fixed order of scans rather than counterbalancing the order of the different scans?

The two alternative ways to order the scans that exist, fixing and counterbalancing, have different pros and cons. By fixing the order, our goal is to make the same scans comparable between different measurement sessions. By counterbalancing the order, we could possibly balance some effects changing over the whole scanning session, but with the trade-off that, the amount of “noise” especially in the comparisons of task-based sessions (PVT and n-back) would be greater than the effects associated with day-to-day variability in sleep and other factors of interest (e.g., the same task would be sometimes performed first when the participant is alert and sometimes last when the participant may be tired). The counterbalance approach could be a good choice for a larger number of participants, but for single participant analysis, we believe that fixed-design is a safer option. Since publications on this topic appear to be still lacking, we have considered, as a source of evidence, experiences of other researchers who have conducted or are currently running fMRI studies with multiple sessions for the same participants. The fixed-order design has also been previously used in massive data collection projects like the Human Connectome Project (initial release) and the Human Connectome Project across ages (Harms et al., 2018).

We have now clarified in the text the motivation for a fixed-order design (page 12, lines 460-462): “We selected to fix the order of scans to make sure that the same condition (PVT, resting state, movie, and n-back) would be comparable between different measurement sessions”.

7. If possible, collecting more data is always better, especially for resting state fMRI and movie watching. How long are the proposed movie scans? Can another resting state fMRI scan be added to each session? How long is the scan run for the N-back task?

The total acquisition time in the scanner is 43 minutes, divided into one localizer (1 minute), one PVT task (10 minutes), one resting state acquisition (8 minutes), one movie (9.5 minutes), one n-back task (6.5 minutes), and one T1-weighted imaging (6 minutes). In addition, the PVT and movie tasks include two 30-second washouts at the beginning and end. The total acquisition time does not take into account the subject’s preparation or any possible small breaks that may be needed due to the subject's tiredness. Information about the duration of the movie and n-back tasks has now been included in the text in (page 13, lines 477-481):

“Movie: The participant will watch part 2 from “The Grand Budapest Hotel” by Wes Anderson[43]. Similarly to the PVT stimuli, we introduce a 30-second washout before and after the 9.5-minute movie stimuli. Likewise, the washout is not included in the analysis.

N-back: The participant will perform the same task described in Methods, Behavioral data, Cognitive tasks with no modifications. This task lasts approximately 6.5 minutes.”

After each pilot session, the subject gave feedback on the engagement, tiredness, and general feeling in the scanner. In short, the subject did not feel claustrophobic inside the scanner, but reported tiredness, back and neck discomfort, and difficulty in keeping still after 30 minutes.

Based on these comments, we agreed on the 45-minute scan session as the maximum time the subject feels tolerable. Nevertheless, we do understand that more data is always better. In fact, Birn and colleagues (2013) indicate that increasing the scan length from 5 to 13 minutes improves the reliability of the rs-fMRI and previous literature has shown that 5-7 minutes of rs-fMRI data yields stable estimates of intrinsic connectivity networks (Fox et al., 2005, and Van Dijk et al., 2010). As a compromise between this and the subject's comfort, the subject has agreed on collecting two additional minutes of resting state data. This is recorded (page 13, line 473): "Rest: a 10-minute resting-state fMRI will be included at each session"

> 8. *For the task fMRI data, do the authors plan to analyze the data in the more standard event-related way as well? You may have more power to detect effects with such an approach than looking at functional connectivity during tasks.*

Yes, we will run a mass univariate statistics analysis for the PVT and n-back tasks as a supplementary analysis. We have now included the description of such analysis in the section *Additional Analysis* (page 21, lines 796-811):

"The richness of the collected data will allow several questions to be explored in different ways. Despite we intend to mainly focus on connectivity, we acknowledge some interesting analysis worth-exploring. Many of these additional analyses are checkpoints to further what could be driving our results or serve as sanity checks. Because of their nature, these analyses will therefore be reported in the supplementary results section.

Although connectivity analysis can be applied to all tasks, more traditional methods like standard mass univariate statistics can be used to analyze the PVT and n-back tasks. Consequently, we will run a supplementary analysis for H1 and H2. In this analysis, we will compute the activation maps for the PVT and n-back tasks using nilearn165. Task-related activation will be determined by identifying voxels showing a significant difference in BOLD signal, taking into account the average hemodynamic response. Event-related design matrices will be generated for the PVT tasks, where we will take into account the RT. Block-related design matrices will be built for the n-back task. Results from the first level analysis will be employed to regress the effects of behavioral and lifestyle factors according to each hypothesis. Mean FD will be included as a confound. Statistical validity will be assessed via FSL randomise over 10000 permutations."

> 9. *Pg. 17, where does the 256 x 256 number come from? Power et al. 2011 was 264 regions, and the authors propose 246 regions prior to this.*

We thank the reviewer for spotting this typo. The number has been corrected to 264 X 264. The sentence in (page 19, lines 691-692) now reads: "To estimate the participation coefficient, we will use the 264 x 264 connectivity matrices and a vector of network IDs based on Power and colleagues".

> 10. *Be consistent with the tense used in the text.*

We have reviewed the manuscript and changed the tense accordingly. Nevertheless, tense will be changed in the final version (e.g., most future tense in the introduction and methods will be changed to the past tense).

Reviewer #2:

> *This Initial Research Submission describes a planned dense phenotyping study in which a single individual will participate in 30 MRI scan sessions across 15 weeks as well as behavioral data collection sessions for 19 weeks. Rest, movie, and attention and working memory task data will be collected during fMRI. Smartphones and wearable devices will also record data about factors such as physical activity and sleep. Overall this is an exciting data collection effort and a rigorous preregistration plan. Although the hypotheses themselves are somewhat diffuse and varied, I am enthusiastic about the project and its potential to inform understanding of within-subject relationships between brain activity and functional connectivity, attention and working memory, and sleep and lifestyle factors. I have some questions and comments below that, if addressed, may strengthen the plan.*

We thank reviewer #2 for the positive feedback.

> *Major comments*

> *1. Hypotheses 1 and 2 refer to brain activity. How will brain activity be operationalized here? The methods provide detail about the planned functional connectivity and intersubject correlation analyses and would benefit from additional detail about planned activation analyses.*

We have replaced “activity” with “connectivity” across the manuscript to avoid confusion. In the previous version of the manuscript, we employed “brain activity” as a generic term and not as a term related to brain activation amplitudes. In our study, we focus on functional connectivity as the primary framework because it can be applied to all fMRI tasks. Based on this suggestion and comment by the reviewer #1 we will, however, run a mass univariate statistics analysis for the PVT and n-back tasks and report that as in the *Additional Analysis* section. However, to keep our analyses consistent and compact, we will only focus on connectivity in the main report and present these additional analyses in the supplementary results section.

To further clarify our focus on connectivity, we have added the sentence in (page 3, lines 90-92) “As an index of brain function, we selected functional connectivity (a pattern of statistical dependencies between brain areas), as this method can be applied across all selected experimental conditions.”

Specification in the additional analysis section reads (page 21, lines 796-811): “The richness of the collected data will allow several questions to be explored in different ways. Despite we intend to mainly focus on connectivity, we acknowledge some interesting analysis worth-exploring. Many of these additional analyses are checkpoints to further what could be driving our results or serve as sanity checks. Because of their nature, these analyses will therefore be reported in the supplementary results section.

Although connectivity analysis can be applied to all tasks, more traditional methods like standard mass univariate statistics can be used to analyze the PVT and n-back tasks. Consequently, we will run a supplementary analysis for H1 and H2. In this analysis, we will compute the activation maps for the PVT and n-back tasks using nilearn165. Task-related activation will be determined by identifying voxels showing a significant difference in BOLD signal, taking into account the average hemodynamic response. Event-related design matrices will be generated for the PVT tasks, where we will take into account the RT. Block-related design matrices will be built for the n-back task. Results from the first level analysis will be employed to regress the effects of behavioral and lifestyle factors according to each hypothesis. Mean FD will be included as a confound. Statistical validity will be assessed via FSL randomise over 10000 permutations."

> *2. The manuscript includes a well-thought-out scan plan and detailed description of the tasks. Although I understand the justification for including a working memory task, what is the benefit of using an adaptive design? Stimulus timing parameters that differ across days due to staircasing could introduce confounds in data analyses. In addition, recent work has found that adaptive tasks, such as the stop-signal task (SST) and monetary incentive delay (MID) task, have poor reliability within-subjects (e.g., Rapuano et al., 2022, NeuroImage, An open-access accelerated adult equivalent of the ABCD Study neuroimaging dataset (a-ABCD); Kennedy et al., 2021, Reliability and stability challenges in ABCD Task fMRI data). It would be helpful to include additional rationale for using an adaptive design or consider a non-adaptive version. Related to point (1), I also suggest considering block-contrasts for measuring session-specific brain activity (rather than event-related regressors); Rapuano et al. and Kennedy et al. both show very poor within-subject reliability of trial-evoked activation patterns in the SST and MID.*

Indeed, we selected to use two different designs for the task-based fMRI, a conventional static (non-adaptive) design for the PVT and a more novel adaptive design for the n-back task. In the PVT case, non-adaptive design is well motivated, as this task has high test-retest reliability (Thompson et al., 2022) and there is robust evidence that this task is suited for longitudinal studies examining how changes in environmental or intrinsic factors reflect to cognitive functions (see, e.g., Alfonsi et al., 2020, Plante et al., 2020, Kalanadhabhatta et al., 2021). Whereas PVT is a simple task mostly tapping lower level cognitive functions (i.e., attention), the n-back task taps higher level cognitive functions. In such tasks, there are often complex temporal effects that do not reflect day-to-day fluctuations, but rather some learning-related factors. These factors that probably exist for any working memory task, are well known for the n-back task, as it has been widely used in longitudinal studies. Unless the task is made adaptive, the performance in n-back tasks generally improves over time until it hits a ceiling. This can be explained, for instance, by self-generated strategies that are characteristic to any complex task (see, e.g., Laine et al., 2018, Fellman et al., 2020). What happens at the same time when this learning occurs in the brain is a complex re-organization of the prefrontal-striatal, parietal, and sensory activations that involves both activation increases as well as activation decreases (see Salmi et al., 2018 for a meta-analysis).

Making the task adaptive, is a general strategy to avoid the decrease in the loading of working memory (see Soveri et al., 2017 for a meta-analysis). As the actual target of typical longitudinal n-back studies is learning, the factor that is made adaptive in the task is working memory load (i.e., by increasing the n when the performance progresses). In this study, this would be problematic, as the task would not be comparable across different sessions in terms of working memory. What can be made adaptive, instead, is the perceptual load (see Lavie, 1995). By balancing the perceptual load across sessions, we want to make sure that the task remains challenging over time and that the participant's effort remains on one hand maximal, and on the other hand at a stable level across the multiple measurement points as well as within each session. We agree with the reviewer that the mean difference between the subsequent stimuli (i.e. threshold) could slightly change across the measurement sessions despite the initial learning effect for a new task will be accounted for. However, considering the trade-off that would result from using a non-adaptive working memory task, we think that this is a smaller caveat. More specifically, we want to include a low level feature (changes in grating rotation or sound pitch) as an adaptive factor, because we expect that it will not influence working memory related brain activity (Salo et al., 2017, Salmela et al., 2018, Salmi et al., 2018). To further avoid unfavorable learning effects and considerable changes in the perceptual threshold across measurement sessions, our participant will practice the tasks for two weeks prior to the beginning of the MRI data collection. Based on our pilot data, we can reliably estimate that these learning effects span over seven to ten days initially (see Supplementary Fig. 4). With regards to the task performance, the adaptive task gives us a robust indicator widely used in psychophysics (d') reflecting attentional performance that accounts for the perceptual load, besides other reaction time and accuracy measures.

We do agree that SST and MID tasks have poor test-retest reliability. However, in general, these tasks are structurally very different from the n-back task. This issue raised by the reviewer is very important and so far there are no previous precision functional mapping studies that would have carefully considered this issue. We have successfully used this adaptive n-back task in several studies (e.g., Salo et al. 2017, Salmela et al. 2018, Salmi et al. 2018) and carefully piloted the task for the purpose of this study. Based on these arguments, we think that it is the best option to complement our non-adaptive PVT task.

Finally, to include a measure that directly reflects the variability in the stimuli and perceptual load, we have now included an additional analysis that is based on the threshold. Like PVT, this is expected to reflect the level of attention rather than working memory load and thus it will be reported in the supplementary results section. A description of this analysis is now added to the manuscript (page 22, lines 812-819): "We chose an adaptive n-back test design where the perceptual load varies at each session. By balancing the perceptual load across sessions, we want to make sure that the task remains challenging over time and that the participant's effort remains maximal and at a stable level across both, between and within sessions. To assure our results will reflect the working memory load, we will examine the influence of perceptual load and attention in the n-back task. To this end, we will conduct a separate analysis, where the threshold value obtained with the adaptive n-back task is included as a confound (see Salmela and colleagues). Like the PVT, we expect these results to reflect the level of attention, instead of the working memory."

3. How will the analyses using PVT and n-back task data account for day-to-day differences in task performance? Will brain activity/connectivity changes be related to changing performance for the PVT and staircasing for the n-back task? Will performance be used as a confound regressor? It is not clear whether hypotheses 1 and 2 also include predictions about how task performance will relate to fluctuations in sleep and physical activity, and how this will be tested or taken into account in fMRI analyses.

Our hypothesis is that variations in behavioral factors will impact the task performance, which in turn, will be reflected in the brain connectivity (i.e. brain connectivity changes will be related to the changing performance in PVT and n-back tasks). For example, we expect that lack of sleep will be correlated with slower reaction times in the PVT task, and those will be reflected as a decrease in the global efficiency (see Table 1, H1).

Because we are interested in the variation of the performance visible from fMRI data, we will not use the task performance as a confound regressor. Relationships of task performance for our selected battery and external factors are implicit in the relationship of the BOLD signal and external factors. However, we understand that this is an important point to be checked. Therefore, to verify that performance and behavioral factors are indeed co-fluctuating, we will run GLM between the task performance and selected behavioral factors.

This has now been explained in the manuscript in (page 20, lines 729-735) “We will not include performance as a confound regressor in the PVT and n-back tasks models because our main focus is the variation of task performance as reflected in brain connectivity. Nevertheless, to verify the relationship between task performance and behavioral factors, we will run two standardized regression analysis between: (1) mean 1/RT and total sleep time, sleep efficiency, and sleep latency for H1, and (2) accuracy and total sleep time, sleep efficiency, sleep latency, steps, and inactive time for H2. Results from these analyses will be reported in the supplementary results section.”

4. The inclusion of naturalistic data is a strength of this study. Previous work, however, suggests that participants' engagement with naturalistic stimuli such as movies modulates ISC (e.g., Son et al., 2021, PNAS). Given that the participant will watch the same movie 30 times, they will probably be less and less engaged by the story over time, likely affecting ISC. Does this change the projected number of sessions needed to see stable ISC ("For naturalistic stimuli previous work(111) has established that 30 scans are within the optimal number of samples for the ISC statistics to converge."), since this previous work was based on participants' first viewing of a naturalistic stimulus, and here repeated viewing may significantly impact the individual's cognitive state dynamics during the movie? Is it feasible to measure the participant's engagement with the movie (e.g., with eye tracking physiological measures, and/or subjective report)?

These are excellent points. We added a questionnaire to be answered at the end of each scanning session to measure the participant’s engagement in each task. In the manuscript, this is explained in the following manner (pages 11-12, lines 431-434): “After each fMRI session, the subject will complete a questionnaire about the experience in the scanner. Using a 5-point Likert

scale, the subject will rate the engagement on each task. Additionally, the subject will also write short freeform accounts on the thoughts while performing rs-fMRI and movie-watching tasks”.

Based on our experience with eye tracking conducted during scanning, the subjective report is likely to be a better option here. The pupil size would probably vary, not only due to engagement, but also according to movements of the characters and camera view and brightness of the images in the movie stimulus, and also the data quality may be compromised in comparison to conventional pupil size measurements outside the scanner.

To our knowledge few studies report the engagement variation across different MRI scans when watching the same movie. Wang et al. (2017) report that engagement tends to improve the test retest reliability of functional measures of brain activity; however, they used only two movie sessions within months apart. Despite this, Wang et al. (2017) found that the test-retest reliability for naturalistic stimuli tends to be higher than for resting state, which, they argue, supports the use of these tasks even in clinical applications as longitudinal markers to track disease progression. Studies from other fields also support the idea of repetition, as repeated exposure allows for the need for security and control, two prerequisites for experiencing entertainment (Hoffmann, 2006) and thus, engagement.

So far, pilot data suggests that the ISC values are not greatly affected by the movie repetition (see the newly added Supplementary Figure 12). Moreover, within-subject ISCs are expected to be larger than between-subject ISCs, as between-subject variability is generally higher than within-subject variability in the fMRI data (Thirion et al., 2007) (see Supplementary Figure 11); this is an advantage because variations in the ISC will be easier to spot in our design. We have added an explanation about these topics in the supplementary material in (page 14, lines 1228-1235) “ This may be due to the low number of sessions, but the fact that we use data from the same subject may also play a role, as between-subject variability is generally higher than within-subject variability in fMRI data. Moreover, when examining ISC between different sessions, we observe little difference between them. For example, ISC between the first and second sessions is similar to the ISC between the first and third sessions (see Supplementary Fig. 12). Although there are still limited number of datapoints, we observe that the repetition of the movie does not significantly decrease ISCs, suggesting that the engagement of the subject remains equal across the three movie sessions”.

Regarding the movie, we chose to use “The Grand Budapest Hotel” because this movie has been previously employed in another large data collection effort (Visconti di Oleggio Castello et al., 2020), which allows us to have a point of comparison. Moreover, the film style highlights different faces and expressions, targeting brain activity related to social interactions and face processing. We expect that basic social experiences are well-suited for longitudinal studies as emotions are contagious. On the other hand, complex narratives may be felt less engaging after the plot has been revealed, or may influence higher cognitive functions, for example, moral judgements (Eden et al., 2014).

5. Did the Aalto University Research Ethics Committee approve the participant's participation without compensation? Compensation is often required by review boards.

Aalto University Research Ethics Committee does not require compensation for participants. The authors stated in the accepted ethics document that “Subjects who are part of the research team will not receive any monetary compensation prior, during, or after the experiment. No academic credits or other academic compensation are awarded and the participation is purely voluntary.”, which was accepted by the committee.

We have included this information in (page 7, line 275-278) “Two updates were required to include new collaborators and adjust the timeline and to include the privacy notice for the study. Informed consent will be obtained from the participant. As approved by the committee, the participant will not receive any compensation for this study.”

> *6. The data availability statement states that, "The data use agreement will restrict potential reuses of the data by the receiving party, as the only purpose for receiving the data is peer-review of this registered report." Will DUAs also restrict data re-use for groups that request these data when the final paper is published? The dataset will be a valuable contribution of this study, and clarity about when and how it will be available to the scientific community will be useful.*

The data collected in this experiment will be open to the scientific community for research purposes, for instance, to other research groups that request access to the dataset. So far, we have only collected pilot data which is available to the reviewers only for the purposes related to this registered-report. The sentence the reviewer highlights was indeed confusing and referring only to the pilot data. We have also clarified that the purpose of the pilot data transfer will only be the peer-review process in the sentences (page 23, line 843-846) “The pilot data use agreement will restrict potential reuses of the data by the receiving party, as the only purpose for receiving the data is peer-review of this registered report. Future data collected from the experiment will be shared in a similar fashion across the scientific community for research purposes.”

> *Minor comments*

> *1. It could increase readability to avoid non-standard acronyms such as "FPM".*

We have revised the manuscript, now avoiding the use of non-standard acronyms when possible or replacing them with their standard version throughout the document.

> *2. In the section, "Inter-daily Representational Similarity Analysis," the authors write that the: "behavior similarity matrix will be computed using the Anna-Karenina structure". Is there a reason to expect the AnnaK model to best represent cross-day similarity here? It would be useful to compare the AnnaK model to another model such as nearest neighbors to empirically test this hypothesis.*

We thank the reviewer for this suggestion. We have now added the nearest neighbors model to our set of analysis for the inter-subject representational similarity analysis. This is now included in (page 19, lines 709-713) “The behavior similarity matrix will be computed using two models, the nearest neighbors, and the Anna-Karenina structure based on the behavioral value extracted from the linear combinations of the daily answers to the selected PANAS

questionnaire, daily total sleep time, awake time, restless sleep, sleep efficiency, sleep latency, breathing rate, and heart rate variability".

> *3. Previous work has censored time-points in fMRI data collected during naturalistic tasks (e.g., Finn et al., 2018, Nat Commun), so this could be an option to reduce the influence of high-motion frames on movie-watching data analyses.*

This is an excellent point that was also raised by reviewer #1. We have modified the manuscript to reflect the use of volume censoring for the movie-watching condition. Briefly, we propose that we will censor high movement volumes (FD>0.2) across all sessions for the movie-watching task. This is clarified in (page 15, lines 550-553) "For movie-fMRI, we will detect the volumes with high movements (FD>0.2) in each session and censor them across the 30 sessions. Similar to resting state, if this exclusion leads to losing more than 20% of the data points, we will increase the threshold to FD>0.5". As reviewer #1 pointed out, our subject is an experienced participant who has already demonstrated in several scans that she can stay still in the scanner, it is unlikely that movement would cause as large problems here than it does in conventional cross-sectional studies.

Reviewer #3:

> *This is a pre-registered study that plans to collect and analyze richly sampled neuroimaging, physiological, and behavioral measures from a single individual over 133 days. As studies of this nature are rare, this work and the accompanying dataset are likely to generate valuable information about the links between brain connectivity measures and individual variability (though generalizability to other subjects will need further investigation). To my understanding, several of the current hypotheses are based on prior cross-sectional studies, but have not been shown to directly extend to day-to-day variations in an individual subject, which this work would address. Hypotheses 4 and 5 are more specific to the proposed design. Specific comments and questions are below:*

We thank the reviewer for the constructive comments.

> *1. With regard to one of the editors' previous comments, it seems that still more may be done in terms of formulating hypotheses that leverage the unique longitudinal design (for instance, looking at effects over different time-scales).*

This is a crucial point and we agree with the reviewer and the editor that some of the unique aspects in this design could be leveraged even more broadly. Consequently, we have now added an additional research question Q2 to investigate how functional connectivity is influenced by behavioral, physiological, and lifestyle factors over different time-scales. This new research question contains four hypotheses that expands on the timescales when we can track the co-fluctuation of external factors and brain connectivity.

The additional question and its hypotheses were added to Table 1 to match the content of the text in (page 5, line 173-216) "While the prespecification of the hypotheses H1-H4 and analyses

makes this registered report ideally suited for confirming and extending previous research results, the richness of the data collected in this study allows for further exploratory analyses, in particular for studying timescales. We approach the more exploratory question Q2 through four specific hypotheses:

H5: Fluctuations up to fifteen days of sleep are correlated with brain connectivity in the fronto-parietal, default mode, sensory, and subcortical networks during sustained attention tasks. We investigate how sleep affects brain connectivity on different timescales in terms of days and weeks based on four reasons. Firstly, previous precision functional mapping research shows that sleep duration during the previous week is related to brain cortical thickness, and these effects are stronger from the second to third night before. Secondly, it has been demonstrated that sustained attention networks are sensitive to within-subject variability at different timescales, including days and weeks4. Thirdly, effects of sleep on the PVT task have been studied longitudinally. Findings include significant influence of the duration of sleep on alertness, long-timescale modulating influence of sleep in vigilance performance, and in general, significant co-deterioration in sleep and cognitive performance across long periods of sleep instability. Finally, research on the longitudinal effects of sleep on brain connectivity highlight that poor sleep quality is associated with a decrease in functional connectivity. These findings lead us to hypothesize that sleep will co-fluctuate with vigilance performance, which modulates brain connectivity during attention tasks during days or weeks. Building on H1, we choose to investigate further time-lagged cross-correlations of sleep and brain connectivity.

H6: Fluctuations up to fifteen days of sleep and physical activity are correlated with later brain activity in the default mode network, fronto-parietal network, and motor areas during working memory tasks. Similarly to H5, we leverage on a previous hypothesis (H2) and investigate the influence of sleep and physical activity on brain connectivity across different timescales, taking into account three factors. Firstly, positive longitudinal effects of sleep and physical activity on improving working memory performance have already been investigated and there is evidence of a close relationship between motor and working memory development. Secondly, better n-back performance and increased sleep and physical activity are positively correlated. Finally, evidence from longitudinal effects of sleep and physical activity on brain connectivity suggest that flexibility of a set of brain regions increased when performing a working memory task following low sleep episodes.

H7: Fluctuations up to fifteen days of sleep, ANS activity, and mood are correlated with later brain connectivity in the default mode, frontoparietal, and subcortical networks during resting-state fMRI. Following the H5 and H6 rationale, we further investigate the effects of sleep, ANS activity, and mood on resting-state brain connectivity at different timescales. We leverage on previous longitudinal findings that have demonstrated that for rs-fMRI, increased sleep regularity is associated with a more efficient network structure, better sleep quality is associated with an increase in FC, and heart rate variability is associated with the functional connectivity trajectory over time when recovering from a head injury. Similarly, using data from my connectome, Mirchi and colleagues demonstrated that resting state brain connectivity patterns

track daily fluctuations in mood, with positive mood being marked by an integrated architecture of functional connectivity."

In addition, we have also added the section "Cross-correlation analysis" two paragraphs to explain the analysis method that will be used to test the new hypotheses (page 20, lines 743-761): "To understand on what timescales behavioral, physiological, and lifestyle factors have effects on functional brain connectivity, we will run a series of time-lagged cross-correlation analyses. This analysis will help us identify the relationship between functional brain connectivity estimates (participation coefficient and global efficiency) and past lags of the external factors. For each ROI, we will compute the lagged cross-correlation between pairs of brain connectivity estimates and external factors, according to the hypothesis being tested. This means that, e.g., for H5, we will compute the cross-correlation between total sleep duration and global efficiency for each of the DMN ROIs. Lagged-cross correlation coefficients will be organized in carpet plots where the x-axis will represent lags (in days) and y-axis will represent ROIs. This carpet plot will be organized by behavioral factor, so following the example, this visualization will yield a total sleep duration carpet plot for all ROIs in H5 across 15 days.

Statistical significance will be evaluated by estimating a null model with 10000 nonparametric permutations between brain connectivity estimates and surrogate behavioral data. For each permutation surrogate behavioral data is synthesized using spectral synthesis, by shuffling the phase of the Fourier transform of each behavioral predictor and applying the inverse Fourier transform. The surrogate behavioral data is then correlated with a randomly selected ROI for the estimation of the null model. . P-values are then obtained from the null-model probability density function and corrected for multiple comparisons with the same approach used in other analysis (FDR)"

Given Q2 has been added, we have also slightly modified Q1 (page 3, lines 98-99) as: "How is functional brain connectivity influenced by previous day behavioral, physiological, and lifestyle factors?"

> *2. The MRI tasks will be carried out in the same order for each scan. I wondered why the decision was not made to counterbalance MRI tasks, in light of potential systematic effects over the course of the scan (such as drowsiness)?*

This is an important question that was also raised by reviewer #1. We carefully balanced the pros and cons of each possible way to order the scans (fixed vs. counterbalanced). With the scans in a fixed order, we aim to make the same scans comparable between different measurement sessions. On the other hand, we could possibly balance some effects changing over the whole scanning session by counterbalancing; however, this option will have the trade off that, for instance, the amount of "noise" especially in the comparisons of task-based sessions (PVT and n-back) would be greater than the effects associated with day-to-day variability in sleep and other factors of interest (e.g., the same task would be sometimes performed first when the participant is alert and sometimes last when the participant may be tired). If the amount of participants would be larger, the additional noise could be better accounted for, but for single participant analysis, we believe that fixed-design is a safer option. We have been in

contact with researchers who have conducted or are currently running fMRI studies with multiple sessions for the same participants and need to consider that as a source of evidence as publications on this topic appear to be still lacking. The fixed-order design has also been used in other data collection projects like the Human Connectome Project (initial release) and the Human Connectome Project across ages (Harms et al., 2018).

We have now clarified in the text the motivation for a fixed-order design (page 12, lines 460-462): “We selected to fix the order of scans to make sure that the same condition (PVT, resting state, movie, and n-back) would be comparable between different measurement sessions”.

> *3. The motivation for selecting participation coefficient and global efficiency as the two graph metrics of interest wasn't clear to me, and it would help to provide the rationale.*

There are two reasons to use these measurements. Firstly, they have been previously used to successfully analyze another functional precision mapping dataset (Mirchi et al., 2019). Secondly, these measurements offer two complementary points of view from the network perspective, as they represent between and within network measurements, allowing us to understand the connectivity inside each network and its relationship with other networks. We have included a sentence to clarify this rationale in the subnetwork measurements in (page 19, lines 687-689) “We opt for these two graph metrics as they have been previously used to analyze a precision functional mapping dataset, and they include both between and within-network measurements.”.

> *4. Hypothesis 2 relates to changes in several brain networks during a working memory task. The working memory task proposed here is an adaptive n-back task, aimed at keeping overall performance constant. However, I would assume that since behavior will vary across days, so will the adaptive difficulty levels, which may be important to account for in the analyses. I didn't find this mentioned.*

Adaptivity in the n-back task is built at trial-level. The idea here is to constantly keep the difficulty at maximum level in terms of perceptual load, without varying the working memory load. With this, we want to avoid the task getting easier in every measurement due to learning and to make sure that the participant will not reach the ceiling level. The index that reflects task difficulty d’ indeed is the metric reflecting the performance that the participant is able to reach at any given measurement. Changes in the task across the study are expected to be minor, since the subject will practice the task for fifteen days prior to the MRI measurement (to avoid initial learning effects). We have now computed the task difficulty d’ for auditory and visual stimuli separately for the pilot data (see Supplementary Fig. 4). As seen in this plot, the difficulty levels decrease drastically and are kept stable for visual gratings (1-back and 2-back) and auditory stimuli for 1-back. Higher fluctuation is seen in the auditory stimuli for the 2-back. This is explained in (page 5, lines 1002-1005) in the supplementary file “The task difficulty threshold also shows the learning process and stability of the task difficulty over time. For changes in visual gratings, we see a stability in d’ after three days for the 1-back task, and seven days for the 2-back task. Similarly, for changes in the pitch changes is observed for 1-back tasks after five days.”

Nevertheless, it is a good idea to account for the effects of the threshold. We have now added an analysis where d’ is included as a regressor in the GLM model for H2 in the Additional Analysis section, (page 22, lines 812-819) “We chose an adaptive n-back test design where the perceptual load varies at each session. By balancing the perceptual load across sessions, we want to make sure that the task remains challenging over time and that the participant’s effort remains maximal and at a stable level across both, between and within sessions. To assure our results will reflect the working memory load, we will examine the influence of perceptual load and attention in the n-back task. To this end, we will conduct a separate analysis, where the threshold value obtained with the adaptive n-back task is included as a confound (see Salmela and colleagues). Like the PVT, we expect these results to reflect the level of attention, instead of the working memory”

5. In the section on quality control, it would be helpful to provide information about quality assurance of variables acquired in the scanner, such as the respiratory, pulse, and eye-tracking measures, which can be susceptible to artifacts.

We have added a description of quality controls for these data in (page 14, lines 508-511) “We will also check the integrity of the eye-tracking, heart rate, and respiration rate data collected in the scanner to look for possible artifacts. In case more than 20% of the data per session is corrupted (e.g., outliers, NaNs, etc.), we will investigate the causes and take corrective actions for the next session to avoid data loss.”

6. The subject will be instructed to keep eyes open during the tasks, but eye-closures might sill occur. Since eye-tracking data will be collected, including a regressor to model the eye opening/closing may benefit the connectivity analysis in this study.

This is a good point. Indeed, eye-closures may occur in the sessions. Here, we propose we use the eye-tracking data as a quality control step to verify that the subject has had the eyes open for at least 80% of the time. If this condition is not met, then a vector can be included in the regression analysis. This vector will label the days where the eyes have been closed for more than 20% of the time in a task. We have included this information in (page 20, lines 736-738) “Regarding H3, we will include the percentage of prolonged eyes closure as a covariate, i.e. the percentage of time when the eyes have been closed for longer than ten seconds (median brief microsleep episodes)”. Since the PVT and n-back tasks performance depend on vigilance, information about eyes closures will be implicit in the task performance.

7. In lines 596-598, it is mentioned that spatial smoothing will be applied only to the movie-watching task. Why apply this step only to the movie scans?

We thank the reviewer for spotting this mistake. No spatial smoothing will be applied for the connectivity analysis. Previous studies (Alakörkkö et al., 2017, Triana et al., 2020) have demonstrated that spatial smoothing affects connectivity measurements and that these changes are non-uniform and systematic. Consequently, we prefer to avoid spatial smoothing when conducting connectivity analysis. However, we will apply a 6mm FWHM kernel to the voxelwise standard analysis. This is now explained in (page 18, lines 662-667) “No spatial smoothing will be applied for the connectivity analysis, as previous research has shown that spatial smoothing

affects connectivity measurements in non-uniform and systematic ways. However, for task-related activation analysis (see Additional Analysis), we will apply a 6 mm full width at half maximum (FWHM) kernel to smooth the data spatially using the FSL software. ”

> *8. For the exploratory Hypothesis 5 (time-segment classification), I believe the results will be averaged across temporal segments to create one map per scan? If so, it would seem there is also an opportunity to examine variability in the processing of different scenes in the movie; the authors could consider expanding the analysis.*

This is a great suggestion and we thank the reviewer for it. As Uri Hasson has demonstrated, a movie is indeed a very useful stimulus to investigate temporal scales in the brain. However, we would like to keep the study focused (i.e., mostly include analyses that we can perform for each scan) and straightforward (we mainly focus on the timescales of days and weeks, not in hours or minutes). The suggested analysis would certainly be worth running separately for another report potentially prepared outside this work. It could also be performed by other researchers, as this dataset will be released.

> *9. Supplementary Fig 5(panel e) indicate a drop-off of correlation with global signal in anterior brain regions. A lower SNR in prefrontal regions is also mentioned by the authors, and while it is good that the tSNR values are comparable to some other studies, this may still be an overall limitation of the data acquisition.*

The coil being used in the Advanced Magnetic Imaging Center is originally a 32-channel head coil which has had two coils removed to avoid restrictions in the eye-field, thus becoming a 30-channel head coil. This is indeed a restriction in the data collection that is now mentioned in the supplementary files (page 9, lines 1135-1137) “In this particular head coil, two channels have been removed from the front to avoid restrictions to the visual field. Consequently, some drop-out to the signal at the prefrontal area is expected”.

> *Minor comments:*

> *1. In the Experimental overview, it is noted that the subject will undergo MRI sessions.. and "simultaneously", smartphones and wearables will be used. This could be misinterpreted by some readers, suggesting that the latter will be collected during the MRI scans themselves, so rephrasing may be helpful.*

We replaced the word “simultaneously”. To add more clarity, the sentence now reads (page 8, lines 295-297) “During the nineteen weeks, we will also employ smartphones and wearables to collect objective data outside the scanner about the subject’s behavior”.

> *2. line 624, "Shortly," (?) another word may be more suitable*

We have rewritten some sentences in that paragraph and the sentence and word in question is no longer in the text.

> *3. line 519, "menstrual cycle data will be binarized.." - in what way would this variable be binarized?*

The sentence is confusing and we apologize for it. We will not record all phases of the menstrual cycle, but instead we will record only the menstruation days via the smartphone app. We integrated an evening question regarding menstruation (see Supplementary Table 2, page 17). We also modified the text in page 16, lines 585-586 as: “Secondly, menstruation days will be recorded via free-text in the smartphone app.”

> *4. line 312, auditory and visual gratings -> auditory tones and visual gratings ?*

The word “tones” was added as suggested in page 10, lines 364-365. The sentence now reads “On each trial, auditory tones and visual gratings are presented, and either tone pitch or grating orientation is changed compared to the previous trial”.

> *5. line 313, trial -> trials*

The ongoing trial orientation or tone is changed in comparison with the previous trial only. Thus, we consider the word trial (in singular) should stay.

> *6. line 487, "..volumes.. with high movement will be discarded from the time series because the adjacency matrix does only depend on one run" - was not clear what was meant here.*

The paragraph in question has been modified and the sentence is no longer in the manuscript. We have also added new methods for scrubbing rs-fMRI and movie-fMRI. This is written in page 15, lines 547-554: “rs-fMRI volumes will be excluded because of high movement (i.e., volumes whose FD>0.2). If this exclusion leads to losing more than 20% of the data points, we will use a more flexible threshold (FD<0.5). Scrubbed volumes could be different for each session of the rs-fMRI data. For movie-fMRI, we will detect the volumes with high movements (FD>0.2) in each session and censor them across the 30 sessions. Similar to resting state, if this exclusion leads to losing more than 20% of the data points, we will increase the threshold to FD>0.5. Discarding volumes does not affect the sampling size (i.e. the number of scans to be taken).”

REFERENCES

Alakörkkö, T., Saarimäki, H., Glerean, E., Saramäki, J., & Korhonen, O. (2017). Effects of spatial smoothing on functional brain networks. European Journal of Neuroscience, 46(9), 2471-2480.

Alfonsi, V., Palmizio, R., Rubino, A., Scarpelli, S., Gorgoni, M., D'atri, A., ... & De Gennaro, L. (2020). The association between school start time and sleep duration, sustained attention, and academic performance. Nature and science of sleep, 12, 1161.

Basner, M., Mollicone, D., & Dinges, D. F. (2011). Validity and sensitivity of a brief psychomotor vigilance test (PVT-B) to total and partial sleep deprivation. Acta astronautica, 69(11-12), 949-959.

Birn, R. M. (2012). The role of physiological noise in resting-state functional connectivity. Neuroimage, 62(2), 864-870.

Birn, R. M., Molloy, E. K., Patriat, R., Parker, T., Meier, T. B., Kirk, G. R., ... & Prabhakaran, V. (2013). The effect of scan length on the reliability of resting-state fMRI connectivity estimates. Neuroimage, 83, 550-558.

Eden, A., Tamborini, R., Grizzard, M., Lewis, R., Weber, R., & Prabhu, S. (2014). Repeated exposure to narrative entertainment and the salience of moral intuitions. Journal of Communication, 64(3), 501-520.

Eickhoff, S. B., Milham, M., & Vanderwal, T. (2020). Towards clinical applications of movie fMRI. *NeuroImage*, *217*, 116860.

Fellman, D., Jylkkä, J., Waris, O., Soveri, A., Ritakallio, L., Haga, S., ... & Laine, M. (2020). The role of strategy use in working memory training outcomes. Journal of Memory and Language, 110, 104064.

Fox, M. D., Snyder, A. Z., Vincent, J. L., Corbetta, M., Van Essen, D. C., & Raichle, M. E. (2005). The human brain is intrinsically organized into dynamic, anticorrelated functional networks. Proceedings of the National Academy of Sciences, 102(27), 9673-9678.

Gordon, E. M., Laumann, T. O., Gilmore, A. W., Newbold, D. J., Greene, D. J., Berg, J. J., ... & Dosenbach, N. U. (2017). Precision functional mapping of individual human brains. Neuron, 95(4), 791-807.

Gratton, C., Laumann, T. O., Nielsen, A. N., Greene, D. J., Gordon, E. M., Gilmore, A. W., ... & Petersen, S. E. (2018). Functional brain networks are dominated by stable group and individual factors, not cognitive or daily variation. Neuron, 98(2), 439-452.

Harms, M. P., Somerville, L. H., Ances, B. M., Andersson, J., Barch, D. M., Bastiani, M., ... & Yacoub, E. (2018). Extending the Human Connectome Project across ages: Imaging protocols for the Lifespan Development and Aging projects. Neuroimage, 183, 972-984.

Hoffmann, J. (2006). "Play it again, Sam". A differentiating view on repeated exposure to narrative content in media.

Jääskeläinen, I. P., Sams, M., Glerean, E., & Ahveninen, J. (2021). Movies and narratives as naturalistic stimuli in neuroimaging. *NeuroImage*, *224*, 117445.

Kalanadhabhatta, M., Rahman, T., & Ganesan, D. (2021). Effect of sleep and biobehavioral patterns on multidimensional cognitive performance: longitudinal, in-the-wild study. Journal of Medical Internet Research, 23(2), e23936.

Karama, S., Armony, J., & Beauregard, M. (2011). Film excerpts shown to specifically elicit various affects lead to overlapping activation foci in a large set of symmetrical brain regions in males. *PloS one*, *6*(7), e22343.
Kragel, P. A., & LaBar, K. S. (2015). Multivariate neural biomarkers of emotional states are categorically distinct. *Social cognitive and affective neuroscience*, *10*(11), 1437-1448.
Laine, M., Fellman, D., Waris, O., & Nyman, T. J. (2018). The early effects of external and internal strategies on working memory updating training. Scientific reports, 8(1), 1-12.
Lavie, N. (1995). Perceptual load as a necessary condition for selective attention. Journal of Experimental Psychology: Human perception and performance, 21(3), 451.
Mirchi, N., Betzel, R. F., Bernhardt, B. C., Dagher, A., & Mišić, B. (2019). Tracking mood fluctuations with functional network patterns. Social cognitive and affective neuroscience, 14(1), 47-57.
Murphy, K., Birn, R. M., & Bandettini, P. A. (2013). Resting-state fMRI confounds and cleanup. Neuroimage, 80, 349-359.
Power, J. D., Cohen, A. L., Nelson, S. M., Wig, G. S., Barnes, K. A., Church, J. A., ... & Petersen, S. E. (2011). Functional network organization of the human brain. Neuron, 72(4), 665-678
Plante, D. T., Hagen, E. W., Ravelo, L. A., & Peppard, P. E. (2020). Impaired neurobehavioral alertness quantified by the psychomotor vigilance task is associated with depression in the Wisconsin Sleep Cohort study. Sleep medicine, 67, 66-70.
Salmela, V., Salo, E., Salmi, J., & Alho, K. (2018). Spatiotemporal dynamics of attention networks revealed by representational similarity analysis of EEG and fMRI. Cerebral Cortex, 28(2), 549-560.
Salmi, J., Nyberg, L., & Laine, M. (2018). Working memory training mostly engages general-purpose large-scale networks for learning. Neuroscience & Biobehavioral Reviews, 93, 108-122.
Salo, E., Salmela, V., Salmi, J., Numminen, J., & Alho, K. (2017). Brain activity associated with selective attention, divided attention and distraction. Brain research, 1664, 25-36.
Saarimäki, H., Gotsopoulos, A., Jääskeläinen, I. P., Lampinen, J., Vuilleumier, P., Hari, R., ... & Nummenmaa, L. (2016). Discrete neural signatures of basic emotions. *Cerebral cortex*, *26*(6), 2563-2573.
Saarimäki, H., Glerean, E., Smirnov, D., Mynttinen, H., Jääskeläinen, I. P., Sams, M., & Nummenmaa, L. (2022). Classification of emotion categories based on functional connectivity patterns of the human brain. *NeuroImage*, *247*, 118800.
Soveri, A., Antfolk, J., Karlsson, L., Salo, B., & Laine, M. (2017). Working memory training revisited: A multi-level meta-analysis of n-back training studies. Psychonomic bulletin & review, 24(4), 1077-1096.
Thompson, B. J., Shugart, C., Dennison, K., & Louder, T. J. (2022). Test-retest reliability of the 5-Minute Psychomotor Vigilance Task in working-aged females. Journal of neuroscience methods, 365, 109379.
Triana, A. M., Glerean, E., Saramäki, J., & Korhonen, O. (2020). Effects of spatial smoothing on group-level differences in functional brain networks. Network Neuroscience, 4(3), 556-574.

Van Dijk, K. R., Hedden, T., Venkataraman, A., Evans, K. C., Lazar, S. W., & Buckner, R. L. (2010). Intrinsic functional connectivity as a tool for human connectomics: theory, properties, and optimization. Journal of neurophysiology, 103(1), 297-321.
Visconti di Oleggio Castello, M., Chauhan, V., Jiahui, G., & Gobbini, M. I. (2020). An fMRI dataset in response to “The Grand Budapest Hotel”, a socially-rich, naturalistic movie. Scientific Data, 7(1), 1-9.
Wang, J., Ren, Y., Hu, X., Nguyen, V. T., Guo, L., Han, J., & Guo, C. C. (2017). Test–retest reliability of functional connectivity networks during naturalistic fMRI paradigms. Human brain mapping, 38(4), 2226-2241.
