# Supplementary material for: Longitudinal single-subject neuroimaging study reveals effects of daily environmental, physiological, and lifestyle factors on functional brain connectivity
Source: PLoS Biol. 2024 Oct 8;22(10):e3002797. doi: 10.1371/journal.pbio.3002797 (PMC11460715; doi:10.1371/journal.pbio.3002797)
Supplement: Attachment — Submitted filename: Reply_to_Reviewers_v4.pdf [file pbio.3002797.s077.pdf]

Ana María Triana, Juha Salmi, Nick Hayward, Jari Saramäki, Enrico Glerean

Effects of daily environmental, physiological, and lifestyle factors on functional brain connectivity

To the editorial staff and academic editors of PLOS Biology,

We would like to thank all reviewers and editors for their very positive review of our manuscript and for the reviewers' constructive feedback. Below, we address each of the reviewers' comments in detail. We quote the original email from the editors and reviewers in italics. Changes are highlighted in the manuscript in blue font. Deleted sentences are also highlighted in blue and in addition, strikethroughed.

> *Thank you for your patience while we considered your revised manuscript "Effects of daily environmental, physiological, and lifestyle factors in functional brain connectivity" for publication as a Preregistered Research Article at PLOS Biology. This revised version of your manuscript has been evaluated by the PLOS Biology editors, our two Academic Editors and the original reviewers. Based on the reviews, we are likely to accept this manuscript for publication, provided you satisfactorily address the remaining points raised by the reviewers.*
>
> *As you address these items, please take this last chance to review your reference list to ensure that it is complete and correct. If you have cited papers that have been retracted, please include the rationale for doing so in the manuscript text, or remove these references and replace them with relevant current references. Any changes to the reference list should be mentioned in the cover letter that accompanies your revised manuscript.*
>
> *We expect to receive your revised manuscript within two weeks.*

Reviewer #1:

> *The authors addressed all my comments. I only have 2 minor points remaining:*
>
> *Regarding point #2 about censoring at FD>0.2mm, the authors propose to use a threshold of 0.2mm (great!), but if more than 20% of the data exceeds that threshold, they will raise it to 0.5mm. I don't think this 20% criteria is necessary. Increasing the FD to 0.5mm will only lead to including submillimeter movements that cause artifact. We are in agreement that this is unlikely to occur anyway, but you should still have plenty of data even if there are some sessions with more than 20% data loss.*

We agree with the reviewer that having more than 20% of events where FD>0.2mm is unlikely. Consequently, we deleted the 20% criteria and will run the analysis with the FD<0.2mm quality criteria.

Regarding point #3, using the Power ROIs seems fair. Of course, note that the ROIs are not parcels, but spheres. Might I suggest proposing to also run analyses using the Gordon 333 parcels (Gordon et al., 2016 Cerebral Cortex) plus additional subcortical & cerebellar ROIs, which can be defined anatomically (e.g., Freesurfer) or functionally (as in Seitzman et al. 2020, A set of functionally-defined brain regions with improved representations of the subcortex and cerebellum. NeuroImage). This would provide an updated sampling of functional areas across the whole brain.

We thank the reviewer for this suggestion. In the 2020 paper, Seitzman et al. define subcortical and cerebellum areas that they later combine with previously generated parcellations, yielding two sets of ROIs. Set 1 is the combination of ROIs from Power et al., (2011) and an improved set of subcortical and cerebellum ROIs. Set 2 is the combination of Gordon 333 parcels (Gordon et al., 2016) and other subcortical and cerebellum ROIs (spheres). Taking into account reproducibility and reusability of the current work, rather than combining Gordon 333 with parcellations from other tools, we would prefer to use only one source for the ROI definition, i.e. use the two Seitzman ROIs sets to analyze the data. We will use Set 1 to derive the main results, as recommended to us in private communication with the author. Since Set 1 is a more recent atlas based on Power 264 ROIs, set 1 replaces Power 264 ROIs, while set 2 adds the suggested parcellation analysis. This change is visible in Page 16, Line 636 : ”Functional network nodes will be defined based on the sets of brain regions defined by Seitzman and colleagues. In their work, Seitzman and colleagues generated new subcortex and cerebellum regions, which they later combined with previous parcellations from Power and colleagues (set 1) and Gordon and colleagues (set2). Set 1 comprises 300 regions of interest (ROI) and set 2 comprises 394 ROIs. We will employ set 1 to derive the main results in the manuscript. Results using set 2 will be reported in the supplementary material.”

Because of this change in parcellation, we updated the list of networks for each hypothesis to match the naming scheme from Seitzman et al., 2020.

Page 3, Line 105: H1: Fluctuations in sleep patterns are correlated with functional connectivity in the fronto-parietal, default mode, somatomotor, and cingulo-opercular networks during sustained attention tasks.

Page 3, Line 120: H2: Fluctuations in sleep and physical activity patterns are correlated with functional connectivity in the default mode, fronto parietal, and somatomotor networks during working memory tasks.

Page 4, Line 130: H3: Fluctuations in sleep, autonomic nervous system activity, and mood patterns are correlated with functional connectivity in the default mode, frontoparietal, and cingulo opercular networks during resting-state fMRI.

Page 4, Line 170: H5: Sleep patterns experienced over the past fifteen days are correlated with functional connectivity in the fronto-parietal, default mode, somatomotor, and cingulo-opercular networks during sustained attention tasks.

Page 5, Line 187: H6: Sleep and physical activity patterns experienced over the past fifteen days are correlated with later functional connectivity in the default mode, fronto-parietal, and somatomotor networks during working memory tasks.

Page 5, Line 198: H7: Sleep, autonomic nervous system activity, and mood patterns experienced over the past fifteen days are correlated with functional connectivity in the default mode, frontoparietal, and cingulo-opercular networks during resting-state fMRI.

In addition, other minor modifications in the text were made. These changes are:

Page 16, Line 643: This yields a weighted adjacency matrix of size 300 x 300.
Page 16, Line 650: This yields a weighted adjacency matrix of size 300 x 300.
Page 16, Line 661: To estimate the participation coefficient, we will use the 300 x 300 connectivity matrices and a vector of network IDs defined by Seitzman and colleagues (Seitzman et al. 2020).
Page 16, Line 665: To calculate the global efficiency, the 300 x 300 adjacency matrices are subdivided into smaller network-specific matrices

<u>Reviewer #2:</u>

> *I thanks the authors for addressing my comments and am interested in seeing the outcome of this work. As a minor note, I suggest specifying "functional connectivity" throughout (rather than "brain connectivity", which could be interpreted as structural connectivity). I also find the wording "Fluctuations up to fifteen days..." somewhat confusing and suggest clarifying the wording. Does this mean the fluctuations are measured in a time series with 15 observations, or that the period of the fluctuations is 15 days?*

We replaced the term “brain connectivity” with “functional brain connectivity” across the whole manuscript.

We also rephrased Q2 and H5, H6, and H7 to make it clear that the cross correlations have lags ranging from 1 up to 15 previous days. The new hypotheses are:

Page 3, line 94: Q2: Can behavioral, physiological and lifestyle factors influence functional connectivity beyond the previous day, and up to the preceding fifteen days?

Page 4, Line 170: H5: Sleep patterns experienced over the past fifteen days are correlated with functional connectivity in the fronto-parietal, default mode, somatomotor, and cingulo-opercular networks during sustained attention tasks.

Page 5, Line 187: H6: Sleep and physical activity patterns experienced over the past fifteen days are correlated with later functional connectivity in the default mode, fronto-parietal, and somatomotor networks during working memory tasks.

Page 5, Line 198: H7: Sleep, autonomic nervous system activity, and mood patterns experienced over the past fifteen days are correlated with functional connectivity in the default mode, frontoparietal, and cingulo-opercular networks during resting-state fMRI.

All changes are also reflected in the Summary Table. In addition, we updated the text for H5, H6, and H7 in the summary table to clarify that we will run lagged-cross correlation analysis between the external factors from the past fifteen days and the functional brain connectivity estimates of the ROIs in the selected networks for each hypothesis.

Reviewer #3:

> *I believe the authors' revisions have helped to improve the clarity and quality of the manuscript. I have only a few additional minor suggestions on the revised material:*
>
> *As suggested, the authors have now included a potential analysis to examine timescales of relationships between behavioral and neuroimaging data. The modified Q1 states "how is functional brain connectivity influenced by previous day behavioral….?" However, since (to my understanding) a range of days will be queried in the cross-correlation analysis, this wording could be clarified to convey that the analysis considers past history longer than one day.*

We thank the reviewer for raising this question. We are interested in two separate questions. In the first question, our interest is to understand how external factors from the previous day affect today's connectivity patterns (Q1). In the second question, our interest is to understand whether external factors from several previous days still affect today's connectivity. For example, does the duration of sleep from five, seven, ten, etc. days ago still affect today's connectivity? Therefore, one question focuses on the very recent events and models this effect in detail (Q1), while the other question is more exploratory and is focused at understanding the timescales of these effects, up to fifteen days (Q2). To clarify this, we rephrased Q1 to (page 3, line 92): "How do behavioral, physiological, and lifestyle factors from the previous day affect today's functional brain connectivity patterns?". We also rephrased Q2 to (page 3, line 94): "Q2: Can behavioral, physiological and lifestyle factors influence functional connectivity beyond the previous day, up to the preceding fifteen days?".

> *I thank the authors for clarifying the rationale for not counterbalancing the scans. While the description in the reply to reviewers is more extensive, the explanation added to the manuscript was very brief. Expanding a bit on the rationale may be helpful for readers who may have the same question.*

We agree with the reviewer that our explanation is short in the manuscript. We expanded our rationale for not counterbalancing the scans in page 11, line 434, as:
"We opted to fix the order of scans to make sure that the same condition (PVT, resting state, movie, and n-back) would be comparable between different measurement sessions. Another option would have been to counterbalance the order of scans to account for potential effects changing over the whole session. However, a possible drawback of using counterbalancing

could be that the amount of variability brought by the same task sometimes being conducted first and sometimes last (reflecting, for instance, the level of arousal during the scanning session) could be greater than the variability of the external factors of interest. In addition, the fixed-order design has been previously used in massive data collection projects such as the Human Connectome Project. While counterbalancing could be used with a larger number of scanning sessions by treating it as an external confound, in single-participant studies, fixing the order of scans could increase the likelihood in detecting brain behavior interactions while keeping the number of scanning sessions reasonably low."

H6-H7: "leveraged on" -> "leveraged"

We deleted the word "on" in the suggested hypotheses.

Line 578: typo, "different hour or day of the day"

We thank the reviewer for spotting this typo. The words "of the day" are deleted. Page 14, line 540 now reads "If more than 20% of the sessions need to be rescheduled to a different hour or day, time and day of the scanning session will be taken into account in a regressor."

Line 818: "Despite we intend to" -> "Despite our intention to"

We accept the suggestion. Page 19, line 757 now reads "Despite our intention to mainly focus on connectivity, we acknowledge some interesting analysis worth exploring."

In addition to the changes related to the reviewers' comments, we also made a full language revision, including a minor change in the title, from "Effects of daily environmental, physiological, and lifestyle factors **in** functional brain connectivity" to "Effects of daily environmental, physiological, and lifestyle factors **on** functional brain connectivity". Changes about these revisions are also highlighted in blue in the main manuscript.

In addition, we updated the reference list. The changes are:

Anton-Erxleben, K., and Carrasco, M. (2013) was replaced by Kieble et al., 2008 as it was more relevant to the timescales in seconds.
Fedorenko, E. (2014) was deleted from the list as it was not relevant to the topic.
Henriksen et al. (2021) arXiv version was updated as the manuscript was published.
Hari et al., (2010), Harms et al., (2008), Van Essen et al., (2012), Seitzman et al., (2020), and Gordon et al., (2016) were added to the list.
