# Supplementary material for: Longitudinal single-subject neuroimaging study reveals effects of daily environmental, physiological, and lifestyle factors on functional brain connectivity
Source: PLoS Biol. 2024 Oct 8;22(10):e3002797. doi: 10.1371/journal.pbio.3002797 (PMC11460715; doi:10.1371/journal.pbio.3002797)
Supplement: Attachment — Submitted filename: Reply to Reviewers.pdf [file pbio.3002797.s078.pdf]

Ana María Triana, Juha Salmi, Nick Hayward, Jari Saramäki, Enrico Glerean

Effects of daily environmental, physiological, and lifestyle factors on functional brain connectivity

To the editorial staff and academic editors of PLOS Biology,

We would like to thank all reviewers and editors for their very positive review of our manuscript and for the reviewers' constructive feedback. Below, we address each of the reviewers' comments in detail. We quote the original email from the editors and reviewers in italics. Changes are highlighted in the manuscript in blue font. Deleted sentences are also highlighted in blue and in addition, strikethroughed.

> *Thank you for your patience while we considered your revised manuscript "Effects of daily environmental, physiological, and lifestyle factors on functional brain connectivity" for consideration as a Preregistered Research Article at PLOS Biology. Your revised study has now been evaluated by the PLOS Biology editors, the Academic Editors, and the original reviewers.*
>
> *In light of the reviews, which you will find at the end of this email, we are pleased to offer you the opportunity to address the comments from the reviewers in a revision that we anticipate should not take you very long. We will then assess your revised manuscript and your response to the reviewers' comments with our Academic Editor aiming to avoid further rounds of peer-review, although might need to consult with the reviewers, depending on the nature of the revisions.*
>
> *In particular, we would like to flag the departure from the Stage 1 manuscript and encourage you to clarify why so much was lost at the stringent cut-off, which seems a bit surprising. Please also address the other concerns mentioned by the reviewers.*

Thank you for your feedback and the opportunity to address the concerns raised by the reviewers.

Regarding the deviation from the Stage 1 manuscript and the stringent framewise displacement (FD) cut-off of 0.2 mm, it's important to clarify that the observed data loss was primarily associated with only one out of the eight hypotheses. On average, the mean FD for all sessions and tasks was below 0.2, as outlined in Supplementary Figure 14, which complies with the established cut-off criteria. While there were instances of FD exceeding this threshold, these were limited to a small number of volumes, as demonstrated in the violin plots of Supplementary Figure 15. Here, the median values consistently remain below FD < 0.2, with very few outliers.

For the majority of our tasks—PVT, resting-state, and n-back—data exclusion was minimal due to the computational approaches employed. Specifically, for the PVT and n-back tasks, we used adjacency matrices derived from beta series that were extracted using a first-level General

Linear Model (GLM). This model appropriately accounts for head movement by incorporating movement parameters as confounds, thereby obviating the need for volume exclusion in these analyses. In contrast, the resting-state tasks were processed using individual adjacency matrices with the framewise displacement (FD) cut-off of less than 0.2. This approach led to an average volume loss of only 4% per session, which we consider minimal and within acceptable limits for maintaining the integrity of our data set.

In the analysis of Hypothesis 4 which employs the movie-watching task, we implemented pairwise Inter-Subject Correlations (ISC) between sessions, assessing the correlation of time series from identical brain regions across different sessions. This analytical method requires that all sessions keep an equal number of volumes. Consequently, if excessive head motion in a specific volume occurs in any one session, this volume must be excluded across all sessions to maintain uniformity, regardless of its acceptability in other sessions. Such a stringent criterion inherently sets a high threshold for data inclusion, as movement in just one volume can render it unusable across all sessions.

On reviewing the movie-watching data, the average loss due to FD>0.2 was 3.7% per session. However, we noted variability among sessions, often influenced by unforeseeable factors. For example, movie data from session 21 shows high head movement due to an eyelash causing discomfort to the participant.

It is important to clarify that these losses were specific to the pairwise ISC method that we used. Alternative methods, such as those employed in our resting-state analyses which calculate individual adjacency matrices, might not experience the same level of data exclusion. This underscores that our findings and the resulting data loss are contingent upon the methodological choices tailored to each hypothesis's requirements.

In response to these observations, we decided to include a less strict scrubbing method censoring a volume only if its FD exceeded 0.2 for 5 or 10% of the sessions. Initially, we presented results with a 5% threshold in the main manuscript, keeping the stricter FD < 0.2 analysis in the supplementary material. However, to address potential concerns about our methodological rigor, and to ensure clarity and compliance with the Stage 1 manuscript, we have now included the 5% and 10% scrubbing analyses only in the supplementary material, reinstating the original analysis in the main manuscript.

This strategy reflects our commitment to methodological precision and our responsiveness to unforeseen challenges in data collection, particularly in longitudinal studies. Instances of undesired subject movement, for instance due to discomfort or pain during scanning, are comprehensively documented in the released data https://zenodo.org/records/10571956.

In conclusion, we removed the analysis that deviates from the registration and clarified our motivation to include an additional analysis reported in the supplementary section. We believe these revisions and explanations address the reviewers' concerns effectively and underscore our commitment to maintaining high research standards.

Page 31, H4: "To assess the relationship between brain connectivity and behavior (i.e., sleep, ANS, or mood) during movie-watching, we computed Mantel tests between the pairwise ISC for each ROI and the similarity matrix for each behavioral variable. It is important to keep in mind that in order to compute the pairwise ISC for each ROI, all sessions must have comparable time series. Therefore, any volumes scrubbed from one session due to high head movement must inevitably be scrubbed from the other sessions as well. To verify the quality of the data, we computed the total number of scrubbed volumes by session, following the exclusion criteria $FD<0.2$. Upon inspection, we noticed that following such rigorous scrubbing discarded 61% of the volumes for all sessions, leaving only a few untouched segments in the first third part of the film (Supplementary Figure 23).

Using this strict criterion, we found no significant relationship between behavioral factors and brain ISC for any of the parcellations. Nevertheless, when regressing the global signal, we identified a significant relationship between the brain ISC and the previous day's mean HRV ($\rho = -0.19$, $p < 0.05$) in the superior parietal sulcus (MNI coordinates x = -5.5, y = 29.3, z = 44) using the AK model. We also observed a significant relationship between the previous day's mean negative affect ($\rho = -0.19$, $p < 0.05$) in the left middle temporal gyrus (MNI coordinates x = -53.1, y = -11.4, z = -16) using the NN model. Both results were obtained using the alternative parcellation.

In an effort to preserve the high standards of data quality while also keeping more fMRI volumes, we adopted an additional percentage-based scrubbing approach for the movie watching task. This method involves discarding a particular volume from all sessions if it is flagged ($FD>0.2$) in at least a percentage of sessions. For example, for a thirty session sample, a 10% threshold excludes all volumes where $FD>0.2$ in at least three sessions. We applied this percentage-based scrubbing with two values 10% and 5% and we were able to improve the percentage of discarded volumes (5% discarded and 32% discarded respectively for the two percentage-based scrubbing strategies). The results of these scrubbings are reported in the Supplementary Information (Supplementary Figures 24 and 25).

Other external factors were found to be related to the daily-ISC when we analyzed the data with the additional scrubbing percentages and models (Supplementary Table 16). From these results, we highlight the negative relationship between the previous day's restless sleep and ISC in the superior parietal sulcus using the NN model, which achieved levels close to significance for all scrubbing strategies when the global signal regression is applied (Supplementary Table 16). Likewise, the positive relationship between the mean HRV and the ISC activity in the medial superior frontal gyrus, which is consistent with for two scrubbing strategies when the data is analyzed with a second parcellation."

Table 1, H4, Outcomes column: Hypothesis rejected. No associations between brain activity and sleep, autonomic nervous system activity or mood are found.

In addition, we would like you to address the following editorial comments:

** We would like to suggest a different title to improve readability/accuracy: "Longitudinal single-subject neuroimaging study reveals effects of environmental, physiological and lifestyle factors on functional brain connectivity"*

We agree with this suggestion and have changed the title accordingly.

** Please include information about the form of consent (written/oral) given for research involving human participants and if the study has been conducted according to the principles expressed in the Declaration of Helsinki.*

Information about the consent and how the study was conducted are included in the Ethics Information section.

Page 7: "This study was approved by the Aalto University Research Ethics Committee (28th of June, 2019, Helsinki, Finland) and was carried out in accordance with the principles expressed in the Declaration of Helsinki. Two updates were required to include new collaborators, adjust the timeline and to include the privacy notice for the study. Written informed consent was obtained from the participant. As approved by the Committee, the participant did not receive any compensation for this study."

Reviewer #1

Reviewer #1: It was a pleasure to see this previously pre-registered report carried out and to read the results in this manuscript. I commend the authors on an ambitious study that has great potential to move the field forward. I was also heartened that the authors took my previous comments into consideration when running their study. Overall, the manuscript is well written and thorough. I have several comments/questions for the authors to address.

1. Perhaps I am confused about the motion scrubbing threshold, so some clarification is warranted. If I am understanding correctly, an FD threshold of 0.5mm was chosen because a lower threshold (0.2mm) removed too much data. However, in the pilot study an FD threshold of 0.2mm removed very little data. Why would the percentage of data removed at this threshold change so significantly from the pilot study to this study on the same participant? Did something change? When examining the carpet plots as in Power et al., is there no observable motion artifact at timepoints during which there is a spike in FD above 0.2 but below 0.5?

We appreciate the reviewer's positive feedback and apologize for any confusion in our previous submission. To clarify, we did not use an FD threshold of 0.5mm in any of our analyses. The original FD threshold (FD<0.2mm) was kept for all analyses and hypotheses except for the movie-watching task in Hypothesis 4, where we employed the original FD threshold and two additional percentage-based thresholds. These percentage-based thresholds involve censoring a volume if its FD exceeds 0.2mm for a specified percentage of sessions.

In light of the feedback, we recognize that our previous explanation might have been unclear. In this revised submission, we have adhered to the originally proposed scrubbing strategy in the main manuscript, relegating the alternative scrubbing strategies to the Supplementary Information.

Regarding Hypothesis 4, our analysis involved pairwise Inter-Subject Correlations (ISC) between sessions for the movie-watching task. This analysis requires the same number of volumes across all sessions. Consequently, any volume showing excessive head motion in one session requires exclusion across all sessions, regardless of its status in other sessions. This strict inclusion criterion explains the higher data exclusion in this study compared to the pilot study.

Upon reviewing the movie-watching data, the average volume loss due to FD>0.2mm was 3.7% per session. Our protocol, measurement sites, and equipment remained consistent across studies, and the participant was reminded to minimize head movement in each session. However, variability in head motion was influenced by unforeseen factors, for example, discomfort from an eyelash in session 21, muscle activation in the scanner reported in session 16, and discomfort due to pain and sickness in sessions 25 and 26. These incidents likely contributed to the increased head motion observed in some sessions.

We have strived to be as rigorous and methodical as possible in our approach and reporting, ensuring that all pertinent details are transparent and systematically documented. We hope this explanation clarifies the data losses.

> *2. Given previous precision functional mapping papers showing individual variability in functional connectivity and network assignments in particular brain regions, do the authors think that their results are affected by the use of canonical, group-defined ROI's? Would you expect differences in the results if you used individually defined ROI's for your participant? Ideally, one would use individually-defined ROI's. However, identification of these ROI's is not trivial, and so it is understandable if running such analyses is outside the scope of this first study with this dataset. Still, it would be interesting to hear the authors thoughts on how their results may be affected by group-defined vs. individually-defined ROI's.*

We thank the reviewer for their insightful comments and questions. As the reviewer mentioned, previous work identified that group-based parcellations obscure individual differences (Seitzman et al., 2019). Furthermore, individual parcellations have been shown to vary with cognitive states (Salehi et al., 2020a; Salehi et al., 2020b), suggesting that our results were likely influenced by our choice of a group-based parcellation.

We agree that using individualized ROIs could potentially alter the spatial extent of the identified brain regions. For instance, two distinct parcels within the anterior cingulate cortex (ACC) that are separated in a group-based approach could be recognized as a single ACC parcel in an individualized approach. Such differences could mean that while we observe correlations between sleep and two specific links between these ACC parcels and the posterior cingulate cortex (PCC) under a group-based model, the same underlying relationship might manifest as a

single, possibly stronger, link in an individualized parcellation. This does not necessarily change the fundamental relationships observed but may refine our understanding of their spatial dynamics.

Despite the potential advantages of individualized parcellations, we chose a group-based approach for several reasons. It aligns our analysis more closely with the prevailing methodologies in the field, facilitating comparisons with existing literature by providing a common framework. This choice also addresses practical considerations, including the widespread availability of group-based parcellation data and established methods for their application.

We acknowledge the limitations of not using individualized ROIs, as well as their potential to offer more tailored insights into functional connectivity. As suggested, the identification of individual ROIs is complex and was beyond the scope of this initial study. However, we have expanded our discussion of this aspect in the revised manuscript, recognizing the importance of considering both group-defined and individual-defined ROIs in future research.

Page 47, Limitations and future studies: "Finally, we employed group-based atlases[158] to improve the generalizability of our findings because the atlases provide brain regions demonstrated to be reliable across hundreds of subjects. Additionally, group-based parcellations facilitate comparisons with existing literature by providing a common framework. However, recent work has shown that group-based parcellations obscure individual differences[26] and individual parcellations also change with cognitive states[208,209]. While reanalysis with individual parcellations is beyond the scope of this current study, we might speculate that such an approach could not only corroborate the relationships we have observed between external factors and brain connectivity but might also refine our insights into the spatial dynamics of the brain. Therefore, future analyses using individual parcellations per task are needed to understand whether external factors drive reconfigurations in the parcels. In addition, re-analyses of the data with individualized parcellations could provide further insights into the stability and reproducibility of the results."

3. *The authors should consistently report the precise statistics from their analyses throughout the results (i.e., statistic and p-value, not just that the result was significant or not). There are very few instances of actual statistics reported.*

We have thoroughly reviewed the manuscript and have now included the specific statistics and p-values for all reported findings to enhance transparency and detail. Additionally, more comprehensive data, such as the links from Q1 or results from Q2, have been detailed in the Supplementary Tables. We have also integrated clarifying statements within the main text to direct readers to these supplementary resources for further information.

Additionally, we have included a paragraph at the beginning of the results section to guide readers on where to find specific information.

Page 25, Data Validation: "The results presented in the subsequent sections have been corrected for multiple comparisons. Detailed statistics and p-values for these analyses are provided in the Supplementary Information. Additionally, all statistical results are available in the GitHub release. https://github.com/AnaTomomi/effects_externalfactors_on_functionalconnectivity/releases/tag/EF_BC_v1.1"

4. I suggest the authors discuss their results in the context of the findings from Gratton et al. (2018), which demonstrated that individual variability in functional connectivity was predominantly driven by individual-specific features that were largely stable over time. While contributions of day-to-day (session) and task state (e.g., rest vs. task) variation were measurable, the effects were much smaller than those of stable individual features. Thus, it is important for the authors discuss their findings of within-individual day-to-day variability in this context.

We appreciate the reviewer's suggestion to contextualize our findings with those of Gratton et al. (2018). In their study, Gratton and colleagues demonstrated that individual-specific features predominantly drive functional connectivity, showing stability over time. While they noted measurable contributions from day-to-day and task state variations, these effects were minor compared to stable individual features. However, other precision mapping studies, such as those by Mirchi et al. (2019) and Prischet et al. (2020), have observed day-to-day variability with frequent sampling across numerous sessions. This suggests that the minimal contributions of sessions on fMRI variability observed by Gratton may be influenced by factors such as limited session data or a focus on understanding variability sources, rather than day-to-day fluctuations. While connectivity remains predominantly stable, frequent sampling with sufficient power allows us to detect these subtle session-to-session changes. Our study does not primarily focus on comparing day-to-day and task-related variations, but we have now incorporated this discussion into our manuscript to provide a comprehensive context for our findings.

Page 45: "Day-to-day variability of functional connectivity

Recent studies have highlighted that functional brain networks show variability across individuals, sessions, and tasks. While some research suggests these networks remain stable over time[199], others indicate significant variability[200,201]. In particular, it has been demonstrated that although functional networks are fundamentally stable, they show moderate task-based changes that vary significantly among individuals, with minimal yet measurable session-based variability[23]. This minimal variation has been also measured by other researchers using larger frequent sampling datasets [8,27,28], indicating that although connectivity remains predominantly stable, frequent sampling with sufficient power enables the detection of subtle session-to-session changes.

Although our study does not primarily focus on comparing day-to-day and task-related variations, our results offer insights into the variability associated with both daily activities and

task states. We identified distinct factors influencing different tasks at various time points. With our sample size (n=30), we can detect subtle effects that might be missed in smaller datasets.

Scientists have highlighted the significance of stability in functional connectivity for biomarker discovery. Similarly, our findings suggest that while individual-based connectivity shows the most substantial variability, day-to-day variations may also play a crucial role, for example, in analyzing the progression of disorders in personalized medicine. This aspect might provide valuable insights for diagnostics and the development of targeted treatments."

5. *In the Discussion, when using words like "efficiency" and "integration", please operationalize what these terms mean, i.e., do they simply mean increased functional connectivity?*

We thank the reviewer for the feedback. We have defined the terms in the Discussion section.

Page 42, Discussion: "In this work, we used three measures of functional connectivity, (i) the links, which can be defined as correlations between the averaged BOLD signal between ROIs, (ii) the global efficiency, defined as the average inverse shortest path length of the brain regions in a network, and (iii) the participation coefficient, defined as the relation between the number of links connecting a node outside its community and the total number of links for that particular node. Consequently, in this work, increased link connectivity signifies increased correlation between BOLD signals of different brain regions, increased global efficiency (or efficiency) implies more efficient signal transmission from brain regions of a subnetwork due their short pathways, and increased participation coefficient (or integration) implies that on average, the subnetwork ROIs are more functionally connected with ROIs outside of their primary subnetwork."

6. *The Ethics Information section still has "will" statements from the pre-registered report.*

We thank the reviewer for highlighting this error. All relevant statements have been corrected in the revised manuscript.

Page 7: "This study was approved by the Aalto University Research Ethics Committee (28th of June, 2019, Helsinki, Finland) and was carried out in accordance with the approved guidelines. Two updates were required to include new collaborators, adjust the timeline and to include the privacy notice for the study. Written informed consent was obtained from the participant. As approved by the Committee, the participant did not receive any compensation for this study."

Reviewer #2:

Reviewer #2: I applaud the authors for an impressive data collection and analysis effort. The paper is dense with many analyses, but this is justified given the preregistration. Many open questions remain about the specificity of findings to the task types and networks tested here, but I do not recommend adding additional analyses to test them.

My evaluation of this stage 2 submission is below.

1) Whether the introduction, rationale and stated hypotheses are the same as the approved Stage 1 Protocol submission.

Yes

2) Whether the authors adhered precisely to the approved Stage 1 experimental procedures.

Yes. The minor changes to the frame-censoring approach and sleep variables used are sufficiently justified.

3) Whether the data are able to test the authors' proposed hypotheses by satisfying the approved outcome-neutral conditions (such as quality checks, positive controls).

Yes

4) Whether the authors' conclusions are justified given the data.

Partially. The tests of question 1 are generally convincing in showing that yesterday's experiences affect today's functional connectivity patterns. I was unsure exactly how permutation testing and multiple comparisons correction were performed and suggest adding this to the methods if appropriate at this stage of the submission process.

The tests of question 2 left me less convinced of robust relationships between experiences during specific days in the past and today's functional connectivity patterns. The effects appeared less replicable across brain atlas and preprocessing pipelines and the huge number of multiple comparisons raises concerns about false positive results. It would be useful to specify exactly how many tests are being performed.

We appreciate the reviewer's thorough evaluation of our study. To address the concerns regarding our methods, we have made additional clarifications in the "Regression Analysis" subsection regarding how permutation testing was conducted, including specifying the R package used for computation. Additionally, we have expanded on the multiple comparisons correction process in the "Multiple Comparisons Correction" section, and we have now explained how to calculate the total number of tests performed, given they vary in each hypothesis. To ensure a cohesive structure in the document, we relocated the "multiple comparisons correction" section to follow the "cross-correlation analysis" section.

We noted an ambiguity in the Methods section and have taken the opportunity to provide clarification. In the "subnetwork estimates" section, it was explicitly stated that summary participation coefficients are derived by averaging the participation coefficients of the selected network nodes. However, this clarity was lacking for the second network measure, global efficiency. To address this, we have added the same sentence used for participation coefficients to clarify the process for global efficiency, ensuring consistency in explanation.

This summary subnetwork measure is then utilized for all computations in H5, H6, and H7. However, in the "Cross-correlation analysis section," it was mentioned that "The surrogate

behavioral data were then correlated with a randomly selected ROI for the estimation of the null model." Given our prior definition of utilizing the summary of subnetwork measures, permuting the synthetic data with a randomly selected ROI appears inconsistent, as the computations are performed at the level of averages, not individual ROIs. This discrepancy may have led to confusion. Therefore, we have made a slight adjustment to Page 21 by stating, "The surrogate behavioral data were then correlated with the subnetwork's measure (average participation coefficient or average global efficiency) for the estimation of the null model."

Regarding the tests for question 2, we acknowledge the exploratory nature of our analysis, as there are no specific hypotheses about the relationships between past experiences and current connectivity patterns (i.e. which specific lags we should evaluate). As such, we conducted cross-correlation analyses for all possible lags. Nevertheless, these analyses were registered in the Stage 1 submission to avoid cherry picking or p-hacking.

Each hypothesis involves $m = l \times v$ models for a subnetwork, where m represents the number of models, l stands for the number of lags, and v indicates the number of variables. For instance, in Hypothesis 5 (H5), for each of the four subnetworks, the maximum number of models is 45 (15 lags, 3 variables). We treated each of these models as a separate hypothesis test, and we corrected for the comparisons corresponding to the four networks rather than the total of 45 comparisons. This approach assumes lag-variable independence, i.e. a variable v at lag l_1 is independent from a variable v at lag l_2 and variables are independent. However, two critical considerations warrant attention, which may impact the number of models: (i) autocorrelation of lags, which can alter the actual number of independent models, and (ii) potential lagged covariance among variables. Given the paper's scope and length, we have focused on correcting for lag-variable independence. Nevertheless, for transparency, all statistical values are documented in the GIT, enabling readers to reference these p-values and apply corrections as necessary.

To ensure the robustness of our findings, we performed analyses using different parcellations, thresholds, and denoising strategies and assessed the stability of results across these variations. We specifically noted consistent significant results across different analysis approaches in Figures 6, 7, and 8, signaling robustness beyond just statistical significance. This approach allows us to evaluate the consistency of results even when adjusting analysis parameters, providing a more reliable indication of true effects. While we acknowledge the reviewer's observation that certain effects fail to replicate across different analysis methods, we highlight in the Results section those that do replicate. This serves to guide the reader toward the findings that demonstrate consistency across various permutations, thresholds, or denoising strategies, guiding the formulation of new hypotheses for future studies.

Page 25, Data Validation section: "The results presented in the subsequent sections have been corrected for multiple comparisons. Detailed statistics and p-values for these analyses are provided in the Supplementary Information. Additionally, all statistical results are available in the GitHub release."

Page 19, Subnetwork measures section: “Summary global efficiency coefficients were obtained by computing the mean of the global efficiency coefficients of the network nodes.”

Page 20, Regression analysis section: “We used the lmPerm R package”

Page 21, Cross-correlation analysis section: “The surrogate behavioral data were then correlated with the subnetwork’s measure (average participation coefficient or average global efficiency) for the estimation of the null model.”

Page 21, Multiple comparisons correction section: “After conducting the permutation tests, we applied corrections for multiple comparisons. For the network estimates (global efficiency and participation coefficient) in H1, H2, and H3, we corrected for the number of networks for each variable in the model, namely four for H1 and three for H2 and H3. For the links in H1, H2, and H3, we corrected for the number of links of the chosen subnetworks, for each variable in the model.

After conducting permutation models with synthetic data for each subnetwork in H5, H6, and H7, we adjusted for the number of subnetworks, assuming independence for each variable-lag pair. This means we treated variables on consecutive days as independent; for example, variable v at lag l1 is independent from a variable v at lag l2 and variables are independent. However, it is important to note that this assumption may not hold true for all variables, as autocorrelation levels and covariance can vary. For example, sleep patterns may exhibit higher autocorrelation compared to mood patterns or sleep patterns may be correlated to activity patterns. Therefore, while corrections were made for the number of networks in H5 (four), H6 (three), and H7 (three), there are still fifteen lags to consider. We have provided all statistical values in the GIT (see code availability) for transparency. Finally, multiple comparisons corrections for H4 and H8 were performed using FSL’s Randomise tool.”

> *There are also a number of unexplored explanations for the results. For example, is there autocorrelation in the sleep, mood, and activity data? Could what appears to be an effect of past sleep on current FC be the result of, for example, slowly accruing sleep debt over time? Could what looks like an effect of last Monday's mood on this Monday's FC arise because mood tends to be consistent on Mondays? My concrete suggestion is to tone down the interpretation of these effects given their instability and the range of possible explanations (other than the one the authors focus on, which is a direct effect of variable x on day y on day z's FC).*

We appreciate the reviewer's insightful comments and agree that there are several unexplored factors that could contribute to the observed results. In response to the reviewer's concrete suggestion, we have taken steps to tone down the interpretation of these effects in light of the diverse range of possible explanations beyond the direct effect of variable x on day y on day z's functional connectivity. In addition, we have added a paragraph about these possible interpretations in the Discussion section.

Page 47, Discussion: “Sixth, our primary focus was on examining the direct dynamic impact of external variables on brain connectivity. However, it is important to acknowledge several unexplored factors that may contribute to the observed results. For example, we need to consider whether there is autocorrelation in the sleep, mood, and activity data, and how it might influence the models, particularly the lagged-cross correlations. Moreover, what might initially appear as an effect of past sleep on current functional connectivity could potentially stem from factors like gradually accumulating sleep debt over time. Similarly, some effects could be influenced by the differences of the week vs. weekend behavioral patterns. Therefore, while our primary aim has been to establish and demonstrate the existence and measurability of these relationships, it is important to recognize possible unknown influencers, and that many other questions remain open.”

> *5) Whether any post-hoc analyses added by the authors are justified, methodologically sound, and informative.*
>
> *N/A*
>
> *6) Whether, in accordance with the PLOS' data availability policy, data has been made freely available in a public repository. Data files should be appropriately time stamped to show that data was collected after Stage 1 Protocol approval and not before.*
>
> *Yes*
>
> *Other comments:*
>
> *There are a few places in the text where the future tense of the stage 1 submission was not changed to past tense (e.g., "Wearables and smartphone data were collected two weeks before the MRI sessions [began] and two weeks after the MRI sessions end[ed].")*

We revised the manuscript and changed the tenses accordingly.

> *Consider replacing the phrase "sanity check" with something such as "data check".*

We have replaced “sanity check” with “data check” as suggested.

> *I believe that "COP" and "CON" are used at different times to refer to the same functional network.*

We have updated the document to ensure consistent use of acronyms.

> *In H4, please unpack the direction of the observed Anna Karenina effect. Do sessions with higher or lower PAF scores show higher/lower ISC?*

We appreciate the reviewer's insight. In our previous submission, we reported results based on a percentage-based scrubbing strategy, which was different from the protocol. However, prompted by the academic editor's feedback, we carefully reassessed our approach. As a result, in the current submission, we've aligned our methodology with the originally proposed scrubbing

strategy. Consequently, the observed Anna Karenina effect is not present in the reported findings.

> *In the Q2 results section, the text slips into describing functional connectivity as "communication" or "information transfer" between nodes. This is not justified as functional connectivity does not necessarily reflect communication or shared information between regions; it could reflect shared noise for example.*

Thank you for bringing this concern to our attention. While our intention was to provide a simplified explanation of functional connectivity, we acknowledge that it can also reflect shared noise or other factors. We have revised these terms and modified them accordingly.

References

Seitzman, B.A., Gratton, C., Laumann, T.O., Gordon, E.M., Adeyemo, B., Dworetsky, A., Kraus, B.T., Gilmore, A.W., Berg, J.J., Ortega, M., et al. (2019). Trait-like variants in human functional brain networks. Proc. Natl. Acad. Sci. U. S. A. 116, 22851–22861.

Salehi, M., Greene, A.S., Karbasi, A., Shen, X., Scheinost, D., and Constable, R.T. (2020a). There is no single functional atlas even for a single individual: Functional parcel definitions change with task. Neuroimage 208, 116366. 10.1016/j.neuroimage.2019.116366.

Salehi, M., Karbasi, A., Barron, D.S., Scheinost, D., and Constable, R.T. (2020b). Individualized functional networks reconfigure with cognitive state. Neuroimage 206, 116233. 10.1016/j.neuroimage.2019.116233.

Mirchi, N., Betzel, R. F., Bernhardt, B. C., Dagher, A., & Mišić, B. (2019). Tracking mood fluctuations with functional network patterns. Social cognitive and affective neuroscience, 14(1), 47-57.

Pritschet, L., Santander, T., Taylor, C. M., Layher, E., Yu, S., Miller, M. B., ... & Jacobs, E. G. (2020). Functional reorganization of brain networks across the human menstrual cycle. Neuroimage, 220, 117091.
