# Supplementary material for: Longitudinal single-subject neuroimaging study reveals effects of daily environmental, physiological, and lifestyle factors on functional brain connectivity
Source: PLoS Biol. 2024 Oct 8;22(10):e3002797. doi: 10.1371/journal.pbio.3002797 (PMC11460715; doi:10.1371/journal.pbio.3002797)
Supplement: Attachment — Submitted filename: Reply_to_reviewers.pdf [file pbio.3002797.s079.pdf]

Ana María Triana, Juha Salmi, Nick Hayward, Jari Saramäki, Enrico Glerean

Longitudinal single-subject neuroimaging study reveals effects of daily environmental, physiological and lifestyle factors on functional brain connectivity

To the editorial staff and academic editors of PLOS Biology,

We would like to thank all reviewers and editors for their very positive review of our manuscript and for the reviewers' constructive feedback. Below, we address each of the reviewers' comments in detail. We quote the original email from the editors and reviewers in italics. Changes are highlighted in the manuscript in blue font. Deleted sentences are also highlighted in blue and in addition, strikethroughed.

> *Thank you for your patience while we considered your revised manuscript "Longitudinal single-subject neuroimaging study reveals effects of daily environmental, physiological, and lifestyle factors on functional brain connectivity" for publication as a Preregistered Research Article at PLOS Biology. This revised version of your manuscript has been evaluated by the PLOS Biology editors, the Academic Editor and the original reviewers.*
>
> *Based on the reviews and on our Academic Editors' assessment of your revision, we are likely to accept this manuscript for publication, provided you satisfactorily address the remaining points raised by the reviewers. Please also make sure to address the following data and other policy-related requests:*

Thank you for your positive feedback.

> ** I could not find a reference/link to the deposited Stage 1 manuscript anywhere in the paper. Can you please add this reference?*

The deposited Stage 1 manuscript is referenced on page 8. For clarity, we have added a more direct reference in the manuscript:

Page 8: The Stage 1 protocol is available in the Open Science Framework https://osf.io/5hu9c/.

> ** I realized there is one ',' too much in the title. Can you please delete the last ',' after 'physiological'?*

We deleted the last comma.

> ** DATA POLICY:*
>
> *You may be aware of the PLOS Data Policy, which requires that all data be made available without restriction: http://journals.plos.org/plosbiology/s/data-availability. For more information, please also see this editorial: http://dx.doi.org/10.1371/journal.pbio.1001797*

Note that we do not require all raw data. Rather, we ask that all individual quantitative observations that underlie the data summarized in the figures and results of your paper be made available in one of the following forms:

1) Supplementary files (e.g., excel). Please ensure that all data files are uploaded as 'Supporting Information' and are invariably referred to (in the manuscript, figure legends, and the Description field when uploading your files) using the following format verbatim: S1 Data, S2 Data, etc. Multiple panels of a single or even several figures can be included as multiple sheets in one excel file that is saved using exactly the following convention: S1_Data.xlsx (using an underscore).

2) Deposition in a publicly available repository. Please also provide the accession code or a reviewer link so that we may view your data before publication.

Regardless of the method selected, please ensure that you provide the individual numerical values that underlie the summary data displayed in the following figure panels as they are essential for readers to assess your analysis and to reproduce it: S6E, S10, S15 and S41.

NOTE: the numerical data provided should include all replicates AND the way in which the plotted mean and errors were derived (it should not present only the mean/average values).

Please also ensure that figure legends in your manuscript include information on where the underlying data can be found, and ensure your supplemental data file/s has a legend.

Please ensure that your Data Statement in the submission system accurately describes where your data can be found.

Thank you for your guidance regarding the data availability policy. In compliance with the PLOS Data Policy, we have ensured that all individual quantitative observations supporting the findings in our paper are openly accessible.

We have organized the data into two distinct Zenodo releases: (i) Pilot Study Data: Available at Zenodo https://doi.org/10.5281/zenodo.13208496 under the data folder. This dataset includes all preliminary observations and data needed to replicate Supplementary Figures 2-11. (ii) Experimental Data: Available at Zenodo https://zenodo.org/records/10571956. Since access to this dataset is restricted due to GDPR regulations, we have also released the data needed to replicate all analysis plots and the rest of the supplementary figures at https://zenodo.org/doi/10.5281/zenodo.13208811. These data include uncorrected results from statistical tests and .nii.gz files plotted in the figures.

Each repository contains detailed instructions for navigating the files, understanding the organizational structure of the data and code, and includes contact email addresses for any questions. Additionally, we have included information about each figure's data in their legends.

All individual numerical values that underlie the summary data displayed in figure panels S6E, S10, S15, and S41 are provided.

In our manuscript, we have addressed data availability comprehensively and included the necessary references in the figure legends and supplemental files.

Page 24, Data availability: All individual quantitative observations underlying the data summarized in the figures and results are available in two public repositories. Pilot data is available in the Zenodo release[174] at https://doi.org/10.5281/zenodo.13208496. Unprocessed study data can be found in the Zenodo dataset release[175] at https://zenodo.org/records/10571956. Processed results derived from the study data are accessible in the GIT repository[176] at https://zenodo.org/doi/10.5281/zenodo.13208811.

We have referenced the Zenodo releases in the relevant sections of the manuscript and the supplementary information text. During the verification process, we corrected a few typos in the Supplementary Information file, which are highlighted in blue for easy identification. Additionally, we verified and updated Supplementary Figures 8 and 10. The changes to these figures were minimal (e.g., adjustments to axes limits), ensuring that the pilot findings and conclusions remain unchanged.

> *CODE POLICY*
>
> *Per journal policy, if you have generated any custom code during the course of this investigation, please make it available without restrictions. Please ensure that the code is sufficiently well documented and reusable, and that your Data Statement in the Editorial Manager submission system accurately describes where your code can be found.*
>
> *Please note that we cannot accept sole deposition of code in GitHub, as this could be changed after publication. However, you can archive this version of your publicly available GitHub code to Zenodo. Once you do this, it will generate a DOI number, which you will need to provide in the Data Accessibility Statement (you are welcome to also provide the GitHub access information). See the process for doing this here: https://docs.github.com/en/repositories/archiving-a-github-repository/referencing-and-citing-content*

Thank you for your guidance regarding the code availability policy. In compliance with the PLOS Code Policy, we have archived our GitHub repositories to Zenodo and obtained DOIs for the code used in the analysis of both the Pilot and Experimental Data.

We have ensured that the code is well-documented and reusable. The DOI links for the archived code are included in the manuscript and the Data Accessibility Statement.

In our manuscript, we have addressed data and code availability comprehensively, providing clear references to the Zenodo archives.

Page 24, Code availability: All code for the Stage 1 protocol is publicly available at Zenodo https://doi.org/10.5281/zenodo.13208496. The code for the final preprocessing, analyses, and visualization is accessible at Zenodo https://zenodo.org/doi/10.5281/zenodo.13208811. The code is released under the Apache 2.0 license.

> *Reviewer remarks:*
> *Reviewer #1: The authors have addressed all comments.*

We thank the reviewer for their time and commitment.

> *Reviewer #2: The authors have address my comments. I leave the decision to the authors and editor, but personally prefer removing the word "study" from the updated title.*

We thank the reviewer for their time and feedback.

> *I have two minor suggestions to the text.*
>
> *1) "To understand the effect of the previous day's sleep, mood, and ANS activity patterns on resting-state functional connectivity, we run a series of regression models on the unthresholded link-weights within the DMN, FPN, and CON."*
>
> *I believe "run" should be changed to "ran".*

We have replaced the word “run” for “ran”.

> *2) "The majority of the findings showed a positive correlation, indicating that more hours of sleep, awake time in bed, and interruptions during sleep are associated with an increase in brain networks estimates." Please specify which brain network estimates. Does this refer to estimates of global efficiency?*

In this case, brain network estimates refer to both, global efficiency and participation coefficient. We have changed the manuscript to clarify this.

Page 33: The majority of the findings showed a positive correlation, indicating that more hours of sleep, awake time in bed, and interruptions during sleep are associated with an increase in global efficiency and participation coefficient.
